# Effects of Biguanide-PROTACs in Pancreatic Cancer Cells

**DOI:** 10.3390/molecules29225329

**Published:** 2024-11-12

**Authors:** Julie Vatté, Véronique Bourdeau, Gerardo Ferbeyre, Andreea R. Schmitzer

**Affiliations:** 1 Département de Chimie, Faculté des Arts et des Sciences, Université de Montréal, 1375 a. Thérèse Lavoie-Roux, Montréal, QC H2V 0B3, Canada; 2 Département de Biochimie et Médecine Moléculaire, Université de Montréal, Montréal, QC H2V 0B3, Canadag.ferbeyre@umontreal.ca (G.F.); 3 Montréal Cancer Institute, CR-CHUM, Université de Montréal, Montréal, QC H2V 0B3, Canada

**Keywords:** PROTAC, biguanide, pancreatic cancer, mitochondria

## Abstract

This study focuses on the synthesis of Biguanide-PROTACs, formed by conjugating the biguanide motif with a spacer and a ligand for recognition subunits of two E3 ubiquitin ligases. Evaluation of their activity on pancreatic cancer cell (KP4) proliferation established a correlation between membrane permeability and median effective concentration. Mechanistic insights revealed that only two compounds exhibited biguanide-like AMPK activation, while only one hydrophobic compound uniquely altered mitochondrial protein levels. The prospect of developing and expanding the Biguanide-PROTAC library holds several promises, offering potential insights into biguanide mechanisms and the creation of more potent anticancer agents. This study contributes to understanding the intricate interplay between compound structure, permeability, and anticancer activity, paving the way for targeted drug development in pancreatic cancer treatment.

## 1. Introduction

**Figure 1:**
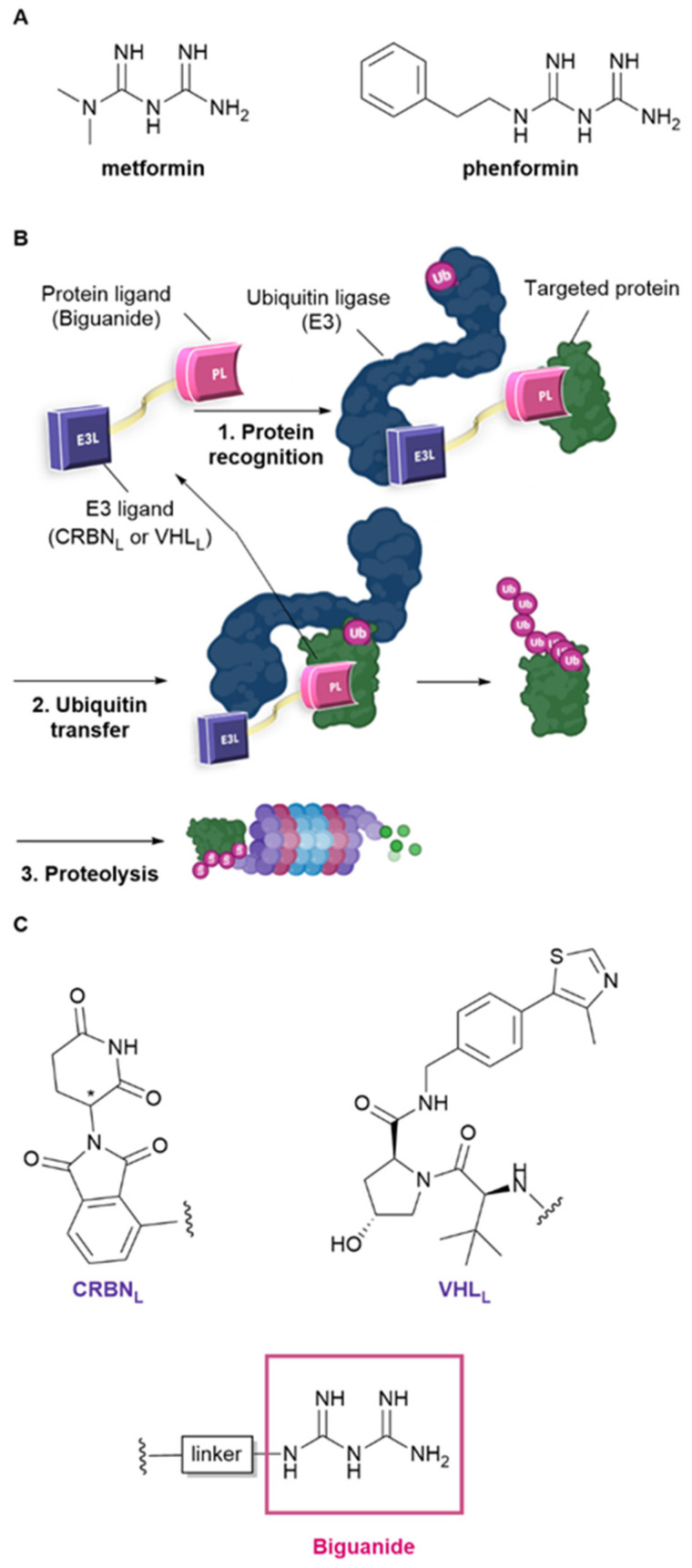
Design and mechanism of Biguanide-PROTAC. (**A**) Structures of metformin and phenformin. (**B**) Mode of action of the PROTAC compound leading to proteolysis. (**C**) Design and structure of Biguanide-PROTACs derivatives targeting the enzyme CRBN or VHL.

Numerous studies have investigated the impact of biguanidium salts, often exemplified by metformin and phenformin (Figure 1A), on the proliferation of various cancer cell types, with a particular focus on their potential in treating pancreatic cancer [1,2,3,4,5,6,7,8]. The challenges posed by late-stage detection have rendered this cancer difficult to manage due to its advanced progression [9]. Furthermore, surgical or radiation interventions are constrained by the intricate anatomical proximity of the tumor to surrounding organs, limiting treatment options primarily to chemotherapy. Recognizing the imperative for novel therapeutic approaches targeting pancreatic cancer, derivatives of biguanides have emerged as promising candidates. These compounds have demonstrated efficacy across diverse pancreatic cell lines and in animal models, exhibiting a notable absence of toxicity or adverse effects [10,11,12,13,14,15]. Nevertheless, a comprehensive understanding of their mechanism of action is an ongoing area of intensive investigation. Unravelling the correlation between their anticancer properties and chemical structure is crucial for refining the design of more potent agents to enhance their therapeutic potential [16,17,18,19]. The antiproliferative activity of biguanidium salts has been attributed to their ability to selectively target mitochondria, owing to their cationic nature. This targeting mechanism culminates in the inhibition of oxidative phosphorylation (OXPHOS) and activation of the AMPK pathway, a key cellular signaling pathway [20,21,22,23,24,25,26,27,28]. It is usually assumed that the activation of AMPK by metformin is a consequence of the inhibition of complex I and OXPHOS [29,30,31]. However recent results show that metformin can activate AMPK via a lysosomal pathway that involves PEN-2 and is independent of OXPHOS [32]. In addition, metformin can directly inhibit the AMP degrading enzyme AMP desaminase in the cytosol [33] and certain studies posit that biguanides exhibit no discernible activity on isolated mitochondria [34].

To delve deeper into the mechanisms of biguanides and pave the way for the development of novel anticancer agents, we have synthesized Biguanide-PROTACs (Proteolysis Targeting Chimeras) (Figure 1B) [35]. These innovative molecules possess a bifunctional structure, comprising two distinct pharmacophores linked by a spacer. The first pharmacophore is designed to target a specific protein of interest, while the second binds to the recognition subunit of an E3 ubiquitin ligase, which could trigger the ubiquitination and degradation of the targeted protein [36]. This unique compound is expected to form a ternary complex, bringing its two binding proteins in close proximity. Subsequently, the transfer of ubiquitin from the E3 enzyme to the protein of interest facilitates its recognition and degradation by the proteasome. This process liberates the PROTAC molecule, allowing it to bind to another protein and initiate a new cycle of degradation [37]. Unlike conventional inhibitors, which require a stoichiometric quantity of ligand to inhibit a protein’s action, PROTACs can be introduced in catalytic quantities. A single molecule of PROTAC has the potential to induce the degradation of multiple proteins. The development of such molecules holds the promise of enhancing the efficacy of biguanide derivatives by facilitating the targeted degradation of their specific protein targets. This approach represents a significant departure from traditional inhibition strategies. Moreover, it is noteworthy that ubiquitin ligase inhibitors, also referred to as E3 ligands, have established themselves as effective anticancer agents in their own right [38]. This underscores the potential synergy between the targeted protein degradation facilitated by Biguanide-PROTACs and the established efficacy of E3 ligand-based anticancer therapies, presenting a multifaceted avenue for advancing cancer treatment strategies.

In addition to potentials in cancer treatments, PROTACs serve as valuable tools for investigating the interaction between a pharmacophore and a targeted protein, as highlighted in the literature [39]. In this context, the development of Biguanide-PROTACs presents an opportunity to experimentally validate the reported interaction between the biguanide moiety and candidate targets. This validation is achieved through the observation of the targeted protein degradation. In pursuit of this goal, we meticulously designed six Biguanide-PROTACs, connecting a biguanide moiety to two distinct E3 ligands via various linkers. These ligands were named CRBN_L_ (ligand for the Cereblon protein) and VHL_L_ (ligand for the Von Hippel Lindau protein) (Figure 1C). The design of these bifunctional molecules was guided by considerations of the linker’s nature and size, as these factors play a pivotal role in facilitating ternary complex formation and determining the efficacy of PROTACs [40].

## 2. Results and Discussion

### 2.1. Design and Synthesis of Biguanide-PROTAC Derivatives

**Scheme 1:**
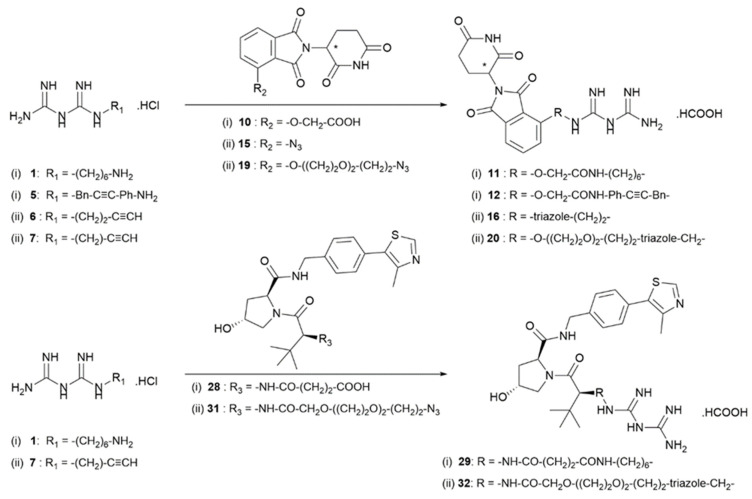
Synthesis of PROTAC-Biguanide compounds based on the CRBN or the VHL ligand. (i) HATU, DIPEA, DMF, r.t, 5–12 h, (**11**: 34%; **12**: 6%; **29**: 7%); (ii) CuI, Na-L-Ascorbate, DMF, r.t, 3–16 h (**16**: 13%; **20**: 14%; **32**: 29%). (* denotes a chiral center and the fact that the compound was used as a racemic mixture).

A range of primary amines underwent condensation with dicyandiamide in the presence of trimethylsilyl chloride, resulting in the synthesis of corresponding biguanide derivatives with yields ranging from 70% to 89% (see Supplementary Information). Compounds **1**, **5**, **6**, and **7** were subsequently engaged in diverse reactions, such as peptide coupling and copper-catalyzed coupling to incorporate the biguanide motif into a library of final compounds. Incorporation of a phenylethynylbenzyle biguanide motif, previously investigated within our research group, further enriched the compound library [15]. This particular motif, known for its intriguing biological properties, especially its ability to traverse phospholipid membranes, served as a pivotal building block (compound **5**) for the synthesis of the desired PROTACs. The synthetic routes for developing Biguanide-PROTACs recruiting CRBN (**11**, **12**, **16**, and **20**) and VHL (**29** and **32**) are depicted in Scheme 1, with detailed information on the preparation of reactants available in the Supplementary Information. The exploration of diverse synthetic pathways involves the independent synthesis of E3 ligands and biguanide derivatives. Each fragment, distinguished by functionalities such as amine, carboxylic acid, azide, or alkyne, exhibits specific roles, allowing them to react synergistically, ultimately yielding the final compounds. Crucially, the linker within these structures can be subject to modification by introducing additional units with complementary functions. This strategic design enables the easy extension of the Biguanide-PROTACs library, fostering versatility and adaptability and also facilitating the fine-tuning of these molecules to optimize their pharmacological properties.

### 2.2. In Vitro Antiproliferative Activity

**Figure 2:**
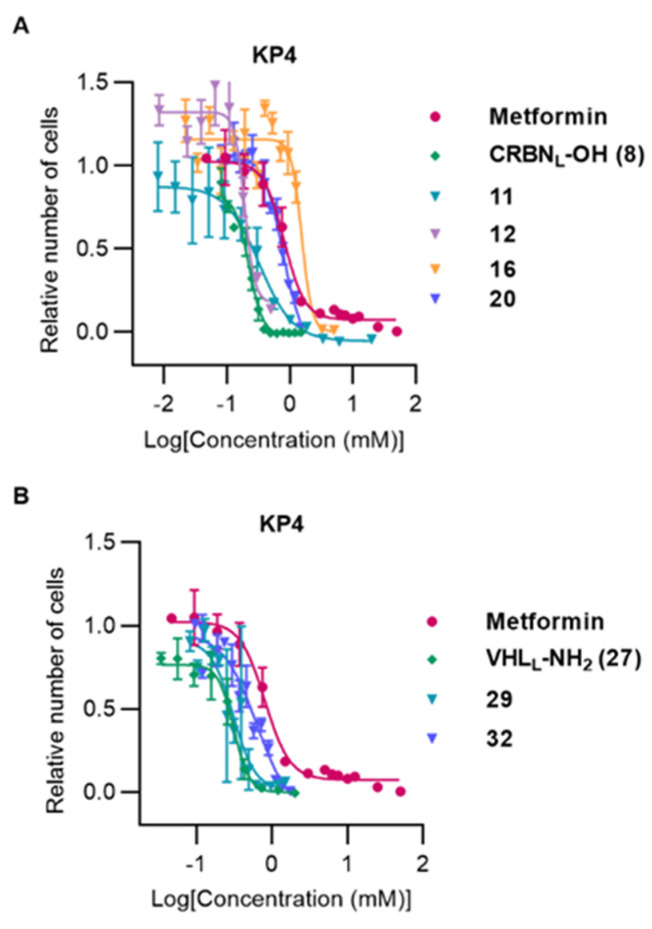
Dose-response curves of compounds Metformin, (**A**) CRBN, and (**B**) VHL ligand derivatives in KP4 cells. Relative number of cells (%) was determined with crystal violet cytotoxicity assay over 72 h.

To evaluate the antiproliferative efficacy of the synthesized Biguanide-PROTACs, a 72-h incubation with various concentrations of these molecules was conducted using a pancreatic cancer cell line (KP4). The concentration required to reach the half maximal concentration affecting growth and viability (EC_50_) was determined for each compound and compared with that of metformin, a widely recognized antiproliferative agent. The antiproliferative properties of all tested compounds were evident, as manifested by their characteristic sigmoidal growth inhibition curves. Interestingly, while compounds **16** and **20** exhibited comparable efficacy to metformin, the majority of the designed PROTACs demonstrated a superior performance, necessitating a lower concentration than metformin to achieve a significant reduction in cell growth and viability (Figure 2).

Prior to conjugation with the biguanide moiety, the CRBN and VHL ligands (designated as **8** and **27**, respectively; see Supplementary Information) underwent testing to assess their individual antiproliferative activities. These ligands, represented by the green curves (Figure 2), exhibited superior antiproliferative effects compared to the CRBN_L_-Biguanide and VHL_L_-Biguanide conjugates. This intriguing observation suggests that the hybridization of E3 ligands with biguanides might influence their binding with the respective enzymes, impacting pharmacodynamics. Additionally, the alteration in physicochemical properties resulting from this conjugation could play a role in affecting pharmacokinetics. The net effect is an intermediate activity observed in the PROTACs, positioning them between the antiproliferative activities of the two parent pharmacophores. The observed relative activity was systematically correlated with the chemical structure of each compound to elucidate a structure-activity relationship. In this endeavor, partition coefficients (cLogP) were calculated to compare the hydrophobicity of the synthesized PROTACs and their respective controls, aiming to establish a discernible trend between their permeability in biological membranes and EC_50_ values (Table 1).

Metformin is the most hydrophilic compound, with a cLogP of −1.63. However, this hydrophilicity poses a limitation, hindering its diffusion across biological membranes. Metformin’s transport relies on organic cationic transporters, ultimately impacting its activity (EC_50_~0.85 mM). In contrast, E3 ligands **8** and **27**, with cLogP values of 0.40 and 2.10, respectively, depend predominantly on their permeability and diffusion across membranes. This heightened membrane permeability likely contributes to their higher antiproliferative activity, with the E3 ligand **8** exhibiting an EC_50_ of approximately 0.17 mM and the E3 ligand **27** displaying an EC_50_ of around 0.34 mM. Variations of hydrophobicity and permeability characteristics underscore the intricate balance between these factors and their consequential impact on the observed biological activities. In most instances, the conjugation of E3 ligands to the biguanide motif resulted in a higher partition coefficient compared to metformin, except for PROTAC **20**, which, due to its polar chain, exhibited increased hydrophilicity. Similarly, PROTAC **16** demonstrated a cLogP value quite similar to that of metformin. Consequently, PROTACs **20** and **16**, being either more hydrophilic or comparably membrane-permeable to metformin, exhibited similar or restricted membrane permeability, reflected by EC_50_ values greater than or equal to that of metformin. Correspondingly, compounds with cLogP values less than 0 displayed the highest EC_50_ values, surpassing 0.34 mM. Conversely, the most apolar PROTACs, namely **11**, **12**, and **29**, exhibited the highest activity against KP4, aligning with the trend that increased permeability can enhance compound efficacy. Although the study highlighted a relationship between cytotoxic activity and permeability, the median inhibitory concentration ranges of the synthesized PROTAC-Biguanides were not significantly distinct from those of their controls, namely metformin and the ligands CRBN_L_ and VHL_L_. Given these observations, it becomes imperative to measure cellular and mitochondrial permeability as well as to delve into the mechanism of action of these compounds to discern whether their activity stems from the biguanide moiety, the E3 enzyme ligand, or if additional mechanisms, such as ternary complex formation, are at play.

### 2.3. Mechanism of Action

**Figure 3:**
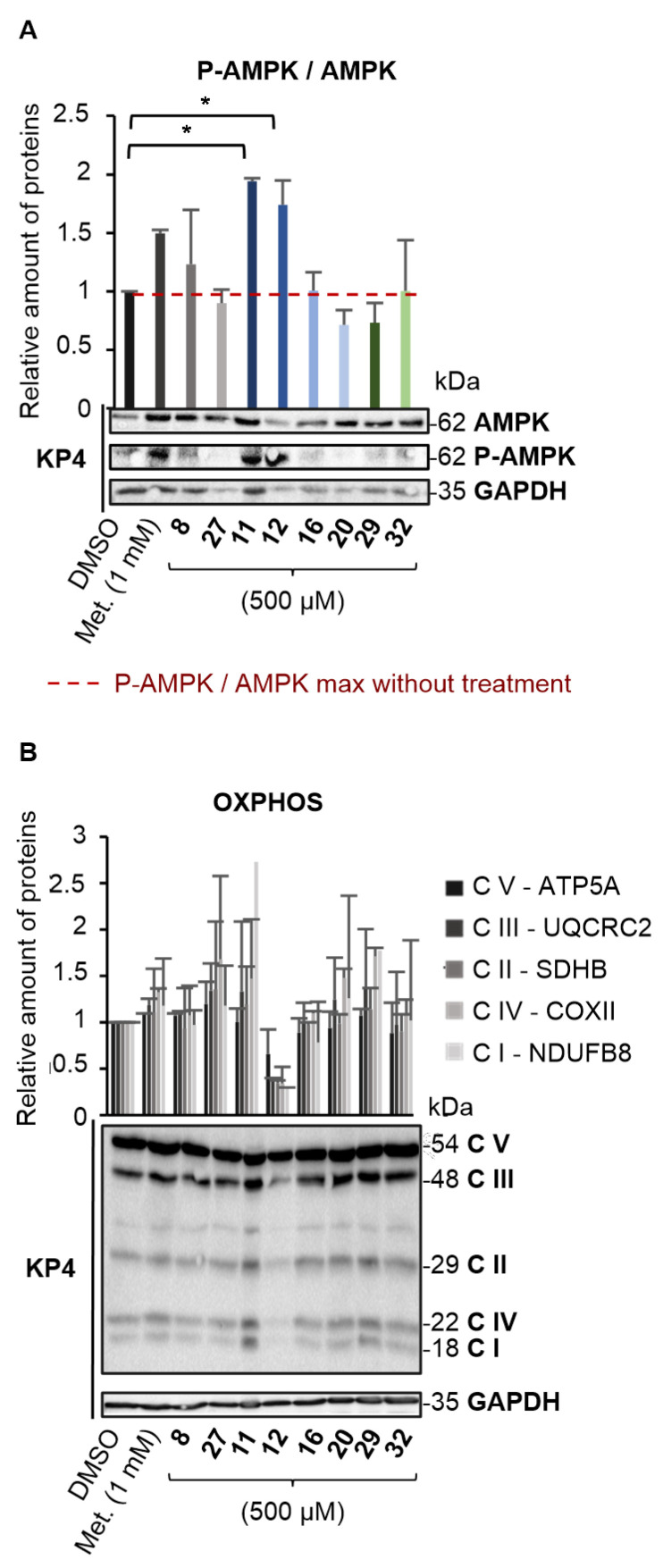
Biguanide-PROTACs effect on P-AMPK and OXPHOS proteins levels. (**A**) Immunoblot and relative quantification of P-AMPK and AMPK levels. (**B**) Immunoblot and relative quantification of OXPHOS proteins levels; both in KP4 after 24 h of treatment. * *p* ≤ 0.05 (ANOVA) (for statistical relevance see Supplementary Information).

The conjugation of the biguanide moiety with an E3 ligand introduces a duality in function, where the compound can either operate as a PROTAC, inducing the formation of a ternary complex leading to target protein degradation, or operate as one of its ligands, binding solely to a single target. To ascertain whether the synthesized compounds could effectively engage with the biguanide target, their capacity to activate the AMPK pathway was investigated. This pathway is known to be activated by phosphorylation in response to the administration of biguanidium salts. In KP4 cells after a 24-h treatment with all Biguanide-PROTACs and their controls, it was observed that compounds **11** and **12** could significatively induce AMPK phosphorylation at the concentrations studied (Figure 3A).

This suggests that these particular compounds operate via their biguanide function, triggering AMPK activation. On the other hand, the activity of the remaining compounds may stem from their exclusive interaction with the E3 enzyme, reinforcing the hypothesis that their mode of action differs from that of metformin and compounds **11** and **12**.

To delve deeper into the mode of action, the levels of OXPHOS proteins from complexes I to V were measured by immunoblot analysis. Notably, the relative quantities of these proteins did not exhibit a significant decrease after treatment with the various compounds, with the exception of compound **12** (Figure 3B). This observation hints at the complexity of the compounds’ interactions within the cellular machinery, prompting further exploration into the specific molecular pathways influenced by each compound.

**Figure 4:**
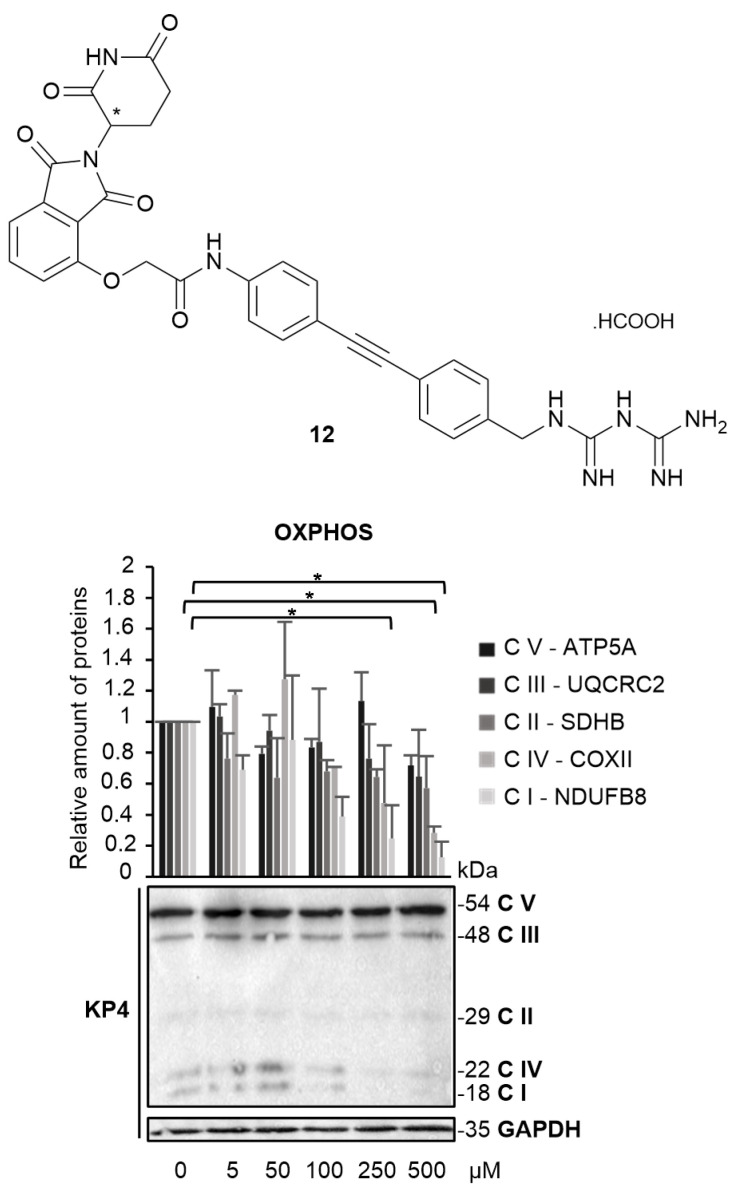
Effect of compound **12** on OXPHOS proteins levels. Immunoblot of OXPHOS proteins in KP4 after 24 h of treatment and relative quantification of protein levels. * *p* ≤ 0.05 (ANOVA) (for statistical relevance see Supplementary Information).

The experiment was subsequently repeated with varying concentrations of these compounds, revealing a subtle yet noteworthy disturbance in proteins COXII (Complex IV) and NDUFB8 (Complex I) at concentrations of 250 µM and above (Figure 4). This observed alteration in protein levels could potentially be attributed to the degradation induced by Biguanide-PROTAC **12**. In this scenario, the proximity between the recognition subunit of the ubiquitin E3 ligase and mitochondrial proteins may have triggered its degradation. Given that compound **12** was the sole compound exhibiting activity, it is plausible to hypothesize that its hydrophobic linker might contribute to its accumulation in the mitochondria. This accumulation could then lead to a degradation of proximal proteins, providing a potential explanation for the observed alteration in the levels of COXII and NDUFB8.

Nevertheless, it is difficult to identify with certainty a specific target for biguanides. Indeed, several studies have shown that mitochondrial respiratory chain complexes can be arranged in different ways, either in in an isolated form or as supercomplexes [41]. In the latter case, the interaction of PROTAC **12** with one of these supercomplexes could lead to protein degradation of several assembled complexes. This insight raises intriguing questions about the specificity and selectivity of these compounds in engaging with cellular targets, underscoring the need for a comprehensive understanding of their mechanism of action for further therapeutic development. Moreover, the motif incorporated into compound **12**, known as phenylethynylbenzylebiguanide or PEB-Biguanide, has been characterized as an amphiphilic cation with the ability to diffuse through phospholipidic membranes, as previously described [15]. The heightened antiproliferative properties of this motif, when compared to metformin, were attributed to its facilitated insertion and accumulation into mitochondria. Compound **8** can act as CRBN inhibitor in the cytosol and results in antiproliferative activity. However, it cannot target mitochondria itself due to the lack of cationic unit; instead, it appears that its conjugation with the PEB-biguanide moiety results in a specific accumulation within mitochondria and shows mitochondrial proteins levels perturbation. At this stage it is impossible to know if there is an additional effect of the biguanide function to the CRBN inhibition, but the decreased level of complex I and IV proteins is only observed for compound **12**. It is important to note that AMPK activation does not correlate with the efficacy of the compounds. AMPK can be activated either by elevated AMP levels following OXPHOS inhibition or in the cytosol through metformin-dependent inhibition of the vacuolar ATPase via PEN2 [32]. Overall, the results suggest that the antiproliferative activity of these compounds is more closely linked to their mitochondrial effects rather than AMPK activation.

## 3. Materials and Methods

### 3.1. General Information for the Synthesis

All chemicals were purchased from Sigma Aldrich (St. Louis, MO, USA), Oakwood Chemicals (West Columbia, SC, USA), and Combi-Blocks (San Diego, CA, USA) in their highest purity and used without further purification. NMR spectra were recorded on AVANCE II 400, Bruker AVANCE NEO 400, Bruker AVANCE 500, and Bruker AVANCE 700 spectrometers (Billerica, MA, USA). Purifications of final compounds were performed on a prep LC-MS (Quadrupole) from Waters (Milford, MA, USA). High-resolution mass spectra (HRMS) and LCMS purity analysis were performed on TOF Agilent instruments (Montréal, QC, Canada) by the regional mass spectrometry center (Université de Montréal).

### 3.2. Synthetic Procedures of Compounds ***1*** to ***32***

6-Aminohexylbiguanide hydrochloride (**1**)

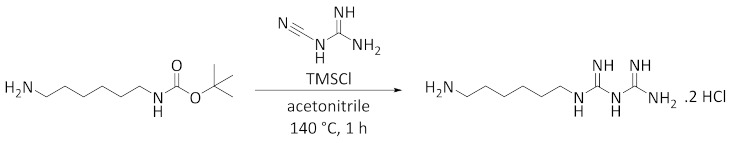



*tert*-Butyl (6-aminohexyl)carbamate (1.22 g, 5.64 mmol) was dissolved in 15 mL of acetonitrile. Dicyandiamide (474 mg, 5.64 mmol) and chlorotrimethylsilane (1.43 mL, 11.30 mmol) were added and the mixture was heated to 140 °C in a sealed tube for 1 h. The solution was cooled to room temperature and 1.4 mL of HCl in dioxane (4 M) was added. The suspension was stirred for 15 min. The precipitate was filtered and washed with ethyl acetate to obtain 1.20 g of a white powder (Yield 78%).

^1^H NMR (500 MHz, DMSO-*d_6_*) δ 9.50 (bs, 1H), 9.09 (bs, 2H), 8.51 (bs, 4H), 8.16 (bs, 2H), 7.71 (bs, 1H), 3.25 (s, 2H), 2.74 (q, *J* = 6.8 Hz, 2H), 1.58–1.52 (m, 4H), 1.35–1.32 (m, 4H)

^13^C NMR (126 MHz, DMSO-*d_6_*) δ 155.5, 152.4, 39.0, 27.2, 26.1, 26.0, 25.9

HRMS: *m*/*z* [M+H]^+^ calcd for C_8_H_20_N_6_, 201.18222; found, 201.18152, [M+Na]^+^ calcd 223.16417; found, 223.16232.

4-Bromobenzylbiguanide hydrochloride (**2**)

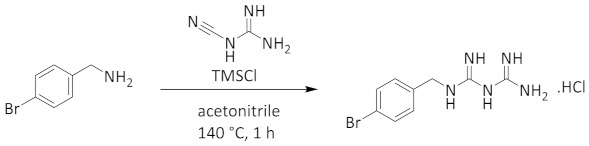



4-Bromobenzylamine (1.48 mL, 11.7 mmol) was dissolved in 15 mL of acetonitrile. Dicyandiamide (983 mg, 11.7 mmol) and chlorotrimethylsilane (2.97 mL, 23.40 mmol) were added and the mixture was heated to 140 °C in a sealed tube for 1 h. The solution was cooled to room temperature and 1 mL of HCl in dioxane (4 M) was added. The suspension was stirred for 15 min. The precipitate was filtered, washed with ethyl acetate, and dried under reduced pressure to obtain 2.52 g of a white powder (Yield 70%).

^1^H NMR (400 MHz, DMSO-*d_6_*) δ 9.89 (bs, 1H), 9.30 (bs, 2H), 8.58 (bs, 4H), 7.57 (d, *J* = 7.9 Hz, 2H), 7.38 (d, *J* = 7.9 Hz, 2H), 4.51 (s, 2H)

^13^C NMR (101 MHz, DMSO-*d_6_*) δ 155.1, 152.4, 131.8, 130.8, 119.2, 66.8, 45.4

LC-MS (ESI): (95% H_2_O + 0.2% FA to 80% MeOH + 0.2% FA in 18 min, DAD 254 nm), t_R_ = 8.69 min, 95% purity

HRMS: *m*/*z* [M+H]^+^ calcd for C_9_H_12_BrN_5_, 270.03488; found, 270.03574

*tert*-Butyl (4-ethynylphenyl)carbamate (**3**)

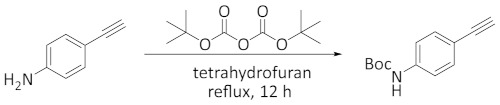



4-Ethynylaniline (2.00 g, 17.1 mmol) and di-*tert*-butyl dicarbonate (4.80 mL, 21.0 mmol) were dissolved in tetrahydrofuran (10 mL) and the solution was cooled to 0 °C in an ice bath. Triethylamine (4.8 mL, 30.4 mmol) was added dropwise and the mixture was heated to reflux for 12 h. The reaction mixture was concentrated under reduced pressure, dissolved in dichloromethane, then washed twice with water and once with brine. The organic layer was dried over MgSO_4_ and then filtered and concentrated under reduced pressure to obtain an orange oil. Hexanes were added and the precipitate was filtered off to obtain 1.81 g of a white powder (Yield 49%).

^1^H NMR (400 MHz, chloroform-*d*) δ 7.48–7.40 (m, 2H), 7.39–7.32 (m, 2H), 6.63 (bs, 1H), 3.04 (s, 1H), 1.54 (s, 9H)

^13^C NMR (101 MHz, chloroform-*d*) δ 152.4, 138.9, 133.0, 118.0, 116.3, 83.6, 81.0, 76.3, 28.3.

*tert*-Butyl(4-((4-((3-carbamimidoylguanidino)methyl)phenyl)ethynyl)phenyl) carbamate (**4**)

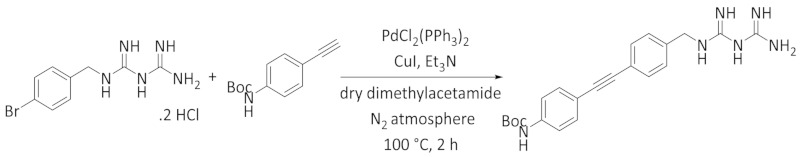



Bromobenzylbiguanide (2.00 g, 6.52 mmol), *tert*-Butyl (4-ethynylphenyl)carbamate (1.45 g, 6.65 mmol), bis(triphenyl-phosphine)palladium(II)chloride (458 mg, 652 µmol), and triphenylphosphine (406 mg, 1.55 mmol) were dissolved in dry dimethylacetamide (43.4 mL). Triethylamine (3.64 mL, 26.1 mmol) was added and the mixture was flushed with N_2_ then heated to 100 °C for 2 h. The solution was cooled to room temperature and filtered through a celite pad, then concentrated under reduced pressure to obtain a dark brown oil. The crude product was purified by chromatography on silica gel with eluent 0 to 40% methanol in dichloromethane to obtain 3.29 g of the product as a yellow powder (Yield 88%).

^1^H NMR (500 MHz, DMSO-*d_6_*) δ 9.61 (bs, 1H), 7.87 (bs, 1H), 7.53–7.47 (m, 4H), 7.45–7.40 (m, 2H), 7.34 (d, *J* = 8.0 Hz, 2H), 7.02 (bs, 5H), 4.38 (d, *J* = 6.0 Hz, 2H), 1.48 (s, 9H)

^13^C NMR (126 MHz, DMSO-*d_6_*) δ 158.5, 152.6, 140.1, 132.0, 131.2, 127.5, 121.2, 117.9, 115.3, 89.5, 88.1, 79.5, 45.3, 43.9, 28.1

LC-MS (ESI): (85% H_2_O + 0.1% FA to 90% MeOH + 0.1% FA in 20 min, +ESI TIC Scan Frag = 150.0 V), t_R_ = 11.75 min, 89% purity

HRMS: *m*/*z* [M+H]^+^ calcd for C_22_H_26_N_6_O_2_, 407.21900; found, 407.21913

4-Aminophenylethynylbenzylbiguanide hydrochloride (**5**)

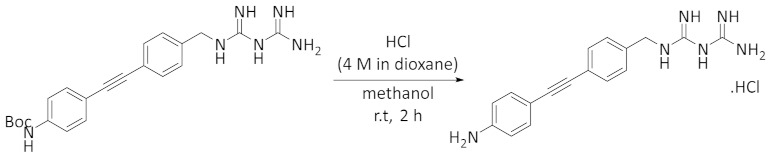



*tert*-Butyl (4-((4-((3-carbamimidoylguanidino)methyl)phenyl)ethynyl)phenyl)carbamate (1.70 g, 4.18 mmol) was dissolved in methanol (5 mL) and hydrochloric acid (10.5 mL, 41.8 mmol, 4 M in dioxane) was added. The mixture was stirred at room temperature for 2 h then concentrated under reduced pressure. Diethyl ether was added and the precipitate was filtered and washed with ethanol and diethyl ether until a clear filtrate is obtained. The solid was dried in vacuo to obtain 1.30 g of a pale brown powder (Yield 90%).

^1^H NMR (500 MHz, DMSO-*d_6_*) δ 9.85 (bs, 1H), 9.23 (bs, 2H), 8.50 (bs, 4H), 7.67–7.49 (m, 4H), 7.46 (d, *J* = 7.9 Hz, 2H), 7.26 (d, *J* = 8.1 Hz, 2H), 4.55 (s, 2H)

^13^C NMR (101 MHz, DMSO-*d_6_*) δ 133.1, 131.8, 131.3, 130.6, 130.1, 129.1, 121.6, 89.4, 45.7, 19.0

LC-MS (ESI): (95% H_2_O + 0.2% FA to 80% MeOH + 0.2% FA in 18 min, DAD 254 nm), t_R_ = 8.41 min, 98% purity

HRMS: *m*/*z* [M+H]^+^ calcd for C_17_H_18_N_6_, 307.16657; found, 307.16712

But-3-yn-1-biguanide hydrochloride (**6**)

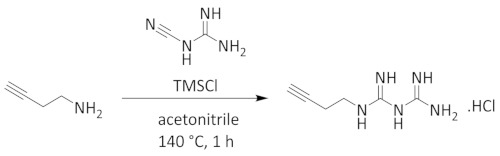



1-Amino-3 butyne (500 mg, 7.24 mmol) was dissolved in 15 mL of acetonitrile. Dicyandiamide (608 mg, 7.24 mmol) and chlorotrimethylsilane (1.84 mL, 14.50 mmol) were added and the mixture was heated to 140 °C in a sealed tube for 1 h. The solution was cooled to room temperature and 1 mL of HCl in dioxane (4 M) was added. The suspension was stirred for 15 min. The precipitate was filtered and washed with ethyl acetate and diethyl ether to obtain 1.37 g of a white powder (Yield 89%).

^1^H NMR (400 MHz, DMSO-*d*_6_) δ 9.58–7.10 (m, 7H), 3.38 (bs, 2H), 2.94 (s, 1H), 2.46 (bs, 2H)

^13^C NMR (126 MHz, DMSO-*d*_6_) δ 155.7, 153.0, 81.7, 73.4, 41.6, 18.2

LC-MS (ESI): (100% H_2_O to 50% MeOH in 15 min, DAD 254 nm), t_R_ = 2.82 min, 91% purity

HRMS: *m*/*z* [M+H]^+^ calcd for C_6_H_11_N_5_, 154.10872; found, 154.10806, [M+2Ag]^2+^ calcd 184.4551; found, 184.45394

Propargyl-biguanide hydrochloride (**7**)

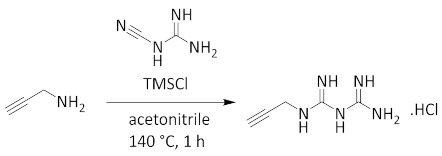



Propargylamine (655 µL, 10.2 mmol) was dissolved in acetonitrile (20 mL). Dicyandiamide (859 mg, 10.2 mmol) and chlorotrimethylsilane (2.59 mL, 20.4 mmol) were added and the mixture was heated to 140 °C in a sealed tube for 1 h. The mixture was cooled down to room temperature. The white precipitate was filtered and washed with acetonitrile and diethyl ether to obtain 1.72 g of the product as a hydrochloride salt (Yield 80%).

^1^H NMR (500 MHz, DMSO-*d_6_*) δ 7.71 (t, *J* = 5.7 Hz, 1H), 7.10 (m, *J* = 58.4 Hz, 6H), 3.94 (dd, *J* = 5.6, 2.5 Hz, 2H), 3.21 (s, 1H)

^13^C NMR (126 MHz, DMSO-*d_6_*) δ 161.0, 158.2, 81.1, 74.3, 30.7

HRMS: *m*/*z* [M+H]+ calcd for C_5_H_9_N_5_, 140.09307; found, 140.09318

2-(2,6-Dioxopiperidin-3-yl)-4-hydroxyisoindoline-1,3-dione (**8**)

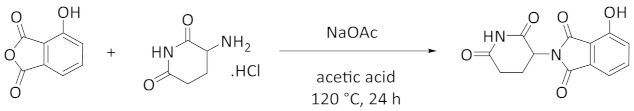



In a round bottom flask, 3-hydroxyphtalic anhydride (2.00 g, 12.20 mmol) was dissolved in 60 mL of acetic acid. Then, 3-aminopiperidine-2,6-dione hydrochloride (2.21 g, 13.40 mmol) and sodium acetate (4 g, 48.70 mmol) were added, and the mixture was heated to 120 °C for 24 h. The solid was filtered off and washed with water to give 2.36 g of a grey powder (Yield 71%).

^1^H NMR (300 MHz, DMSO-*d_6_*) δ 11.17 (bs, 1H), 11.08 (s, 1H), 7.65 (dd, *J* = 8.4, *J* = 8.4, 1H), 7.31 (d, *J* = 7.1 Hz, 1H), 7.24 (d, *J* = 8.3 Hz, 1H), 5.07 (dd, *J* = 12.9, 5.4 Hz, 1H), 2.94–2.82 (m, 1H), 2.65–2.50 (m, 2H), 2.10–1.94 (m, 1H)

^13^C NMR (101 MHz, DMSO-*d_6_*) δ 173.3, 170.5, 167.5, 166.3, 156.1, 136.8, 133.6, 124.1, 114.8, 114.7, 49.1, 31.4, 22.5

*tert*-Butyl 2-((2-(2,6-dioxopiperidin-3-yl)-1,3-dioxoisoindolin-4-yl)oxy)acetate (**9**)

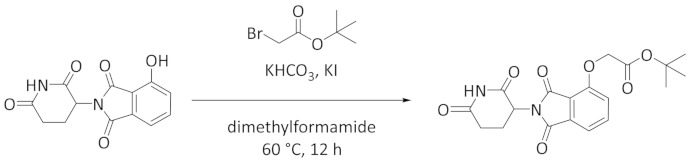



In a round-bottom flask, 2-(2,6-dioxopiperidin-3-yl)-4-hydroxyisoindoline-1,3-dione (1.00 g, 3.65 mmol) was dissolved in dimethylformamide (8 mL). Potassium iodide (80.5 mg, 485 µmol) and potassium hydrogen carbonate (646 mg, 6.45 mmol) were added to the stirred solution. Then *tert*-butyl bromoacetate (808 µL, 5.47 mmol) was added dropwise. The resulting mixture was stirred at 60 °C for 12 h. The solution was brought back to room temperature, diluted with ethyl acetate, and washed three times with brine. The organic layer was dried over MgSO_4_ and concentrated under reduced pressure. The crude product was purified by chromatography on silica gel with eluent 0 to 10% methanol in dichloromethane to obtain 1 g of the O-substituted product as a white powder (Yield 71%).

^1^H NMR (400 MHz, DMSO-*d_6_*) δ 11.11 (s, 1H), 7.80 (dd, *J* = 8.5, 7.3 Hz, 1H), 7.48 (d, *J* = 7.2 Hz, 1H), 7.38 (d, *J* = 8.6 Hz, 1H), 5.10 (dd, *J* = 12.9, 5.4 Hz, 1H), 4.97 (s, 2H), 2.96–2.83 (m, 1H), 2.65–2.51 (m, 2H), 2.05 (ddt, *J* = 12.4, 5.6, 3.5 Hz, 1H), 1.43 (s, 9H)

^13^C NMR (101 MHz, DMSO-*d_6_*) δ 173.3, 170.4, 167.6, 167.2, 165.6, 155.5, 137.2, 133.7, 120.4, 116.9, 116.4, 82.4, 66.0, 49.3, 31.4, 28.2, 22.4

2-((2-(2,6-Dioxopiperidin-3-yl)-1,3-dioxoisoindolin-4-yl)oxy)acetic acid (**10**)

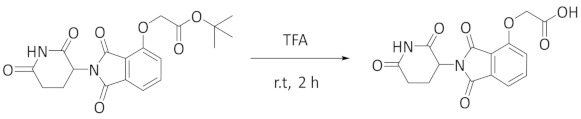



*tert*-Butyl 2-((2-(2,6-dioxopiperidin-3-yl)-1,3-dioxoisoindolin-4-yl)oxy)acetate (1.11 g, 2.85 mmol) was dissolved in trifluoroacetic acid (8 mL). The mixture was stirred at room temperature for 2 h, then concentrated under reduced pressure to give 950 mg of a white solid (Quantitative yield).

^1^H NMR (400 MHz, DMSO-*d_6_*) δ 13.25 (bs, 1H), 11.12 (s, 1H), 7.80 (dd, *J* = 8.5, 7.3 Hz, 1H), 7.49 (d, *J* = 7.3 Hz, 1H), 7.40 (d, *J* = 8.5 Hz, 1H), 5.11 (dd, *J* = 12.9, 5.4 Hz, 1H), 5.00 (s, 2H), 2.97–2.83 (m, 1H), 2.73–2.52 (m, 2H), 2.06 (ddd, *J* = 12.7, 7.2, 3.2 Hz, 1H)

^13^C NMR (101 MHz, DMSO-*d_6_*) δ 173.3, 170.4, 170.0, 167.2, 165.6, 155.6, 137.2, 133.7, 120.3, 116.8, 116.2, 65.5, 49.3, 31.4, 22.4.

*N*-(6-Aminohexylbiguanide)-2-((2-(2,6-dioxopiperidin-3-yl)-1,3-dioxoisoindolin-4-yl)oxy)acetamide formate (**11**).

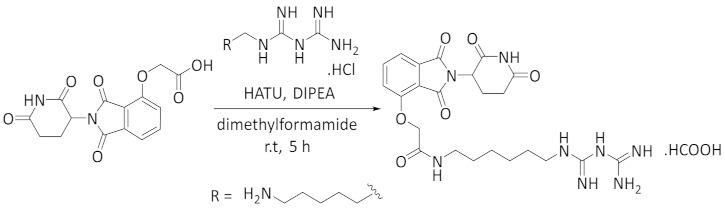



In a round-bottom flask, 2-((2-(2,6-dioxopiperidin-3-yl)-1,3-dioxoisoindolin-4-yl)oxy)acetic acid (628 µL, 1.82 mmol) was dissolved in dimethylformamide (10 mL). HATU (1.04 g, 2.74 mmol) and *N,N*-diisopropylethylamine (1.27 mL, 7.30 mmol) were added and the mixture was stirred for 15 min at room temperature. A solution of 6-aminohexylbiguanide hydrochloride (704 mg, 2.97 mmol) dissolved in dimethylformamide (2 mL) was added and the resulting mixture was stirred at room temperature for 5 h, then purified by HPLC prep and lyophilized to obtain 350 mg of the title compound as an eggshell solid (Yield 34%).

^1^H NMR (400 MHz, DMSO-*d_6_*) δ 11.16 (s, 1H), 8.38 (s, 1H, HCOOH), 8.03 (t, *J* = 5.7 Hz, 1H), 7.82 (dd, *J* = 8.5, 7.3 Hz, 1H), 7.51 (d, *J* = 7.2 Hz, 1H), 7.40 (d, *J* = 8.5 Hz, 1H), 7.01 (bs, 5H), 5.13 (dd, *J* = 12.8, 5.4 Hz, 1H), 4.79 (s, 2H), 4.41 (s, 1H), 3.18 (s, 4H), 3.07 (t, *J* = 7.0 Hz, 2H), 2.91 (ddd, *J* = 16.7, 13.7, 5.3 Hz, 1H), 2.67–2.53 (m, 2H), 2.12–2.00 (m, 1H), 1.44 (t, *J* = 7.1 Hz, 3H), 1.27 (td, *J* = 9.2, 8.0, 4.2 Hz, 4H)

^13^C NMR (101 MHz, DMSO-*d_6_*) δ 173.3, 170.4, 167.2, 167.1, 166.0, 159.2, 155.5, 137.4, 133.5, 127.4, 120.8, 119.0, 117.3, 116.5, 68.1, 49.3, 38.8, 31.4, 29.4, 26.5, 26.4, 22.5

LC-MS (ESI): (95% H_2_O 0.1% FA to 60% ACN in 15 min, DAD 254 nm), t_R_ = 3.84 min, 100% purity.

HRMS: *m*/*z* [M+H]^+^ calcd for C_23_H_30_N_8_O_6_, 515.23611; found, 515.23613.

*N*-(4-((4-((3-Biguanidemethyl)phenyl)ethynyl)phenyl)-2-((2-(2,6-dioxopiperidin-3-yl)-1,3-dioxoisoindolin-4-yl)oxy)acetamide formate (**12**).

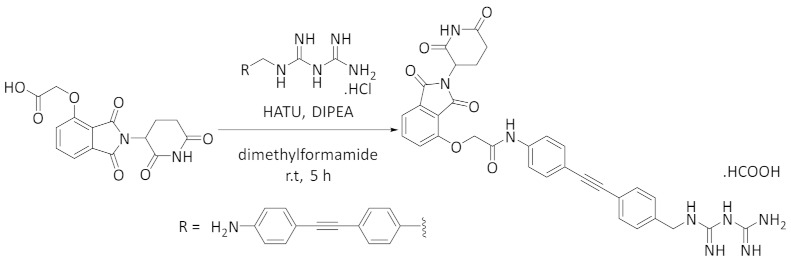



In a round-bottom flask, 2-((2-(2,6-dioxopiperidin-3-yl)-1,3-dioxoisoindolin-4-yl)oxy)acetic acid (330 mg, 993 µmol) was dissolved in dimethylformamide (4 mL). HATU (378 mg, 993 µmol) and *N,N*-diisopropylethylamine (1.00 mL, 5.74 mmol) were added and the mixture was stirred for 15 min at room temperature. A solution of 4-aminophenylethynylbenzylbiguanide hydrochloride (245 mg, 715 µmol) dissolved in dimethylformamide (2 mL) was added and the resulting mixture was stirred at room temperature for 5 h, purified by HPLC prep (despite its poor solubility), and lyophilized to obtain 30 mg of the product as a white solid (Yield 6%).

^1^H NMR (400 MHz, DMSO-*d_6_*) δ 11.14 (s, 1H), 10.36 (bs, 1H), 8.48 (s, 1H, HCOOH), 7.89–7.80 (m, 2H), 7.70 (d, *J* = 8.6 Hz, 2H), 7.62–7.42 (m, 6H), 7.36 (t, *J* = 7.8 Hz, 2H), 7.00 (bs, 5H), 5.15 (dd, *J* = 13.1, 5.2 Hz, 1H), 5.05 (s, 2H), 4.38 (d, *J* = 5.4 Hz, 2H), 2.92–2.87 (m, 1H), 2.62 (d, *J* = 15.2 Hz, 2H), 2.03 (m, 1H).

^13^C NMR (126 MHz, DMSO-*d_6_*) δ 172.8, 169.9, 166.8, 166.0, 165.6, 155.2, 137.0, 133.1, 132.2, 131.2, 127.5, 120.9, 120.5, 119.3, 116.7, 116.1, 67.6, 48.8, 31.0, 22.0.

LC-MS (ESI): (95% H_2_O 0.1% FA to 60% ACN in 15 min, DAD 254 nm), t_R_ = 6.68 min, 95% purity.

HRMS: *m*/*z* [M+H]^+^ calcd for C_32_H_28_N_8_O_6_, 621.22046; found, 621.22043.

2-(2,6-Dioxopiperidin-3-yl)-4-nitroisoindoline-1,3-dione (**13**).

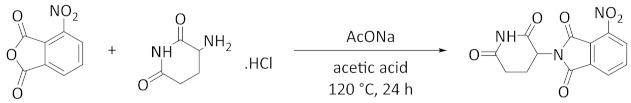



To a round bottom flask were added 3-nitrophtalic anhydride (1.00 g, 5.2 mmol), 3-aminopiperidine-2;6-dione hydrochloride (0.94 g, 5.7 mmol), and sodium acetate (1.70 g, 20.7 mmol). Then, 25 mL of acetic acid was added, and the mixture was heated to 120 °C for 24 h. The solid was filtered off and washed with water to give 1.48 g of a grey powder (Yield 94%).

^1^H NMR (400 MHz, DMSO-*d_6_*) δ 11.18 (s, 1H), 8.37 (d, *J* = 8.1 Hz, 1H), 8.26 (d, *J* = 7.5 Hz, 1H), 8.13 (dd, *J* = 7.8 Hz, *J* = 7.8 Hz, 1H), 5.22 (dd, *J* = 12.9, 5.4 Hz, 1H), 2.91 (m, 1H), 2.63–2.54 (m, 2H), 2.18–2.00 (m, 1H).

^13^C NMR (101 MHz, DMSO-*d_6_*) δ 173.2, 170.0, 165.6, 163.0, 144.9, 137.3, 133.5, 129.3, 127.8, 123.0, 49.9, 31.3, 22.2

4-Amino-2-(2,6-dioxopiperidin-3-yl)isoindoline-1,3-dione (**14**).

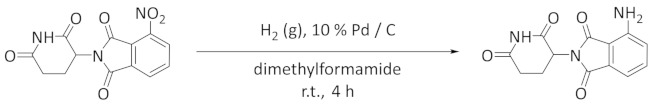



2-(2,6-Dioxopiperidin-3-yl)-4-nitroisoindoline-1,3-dione (533 mg, 1.76 mmol) was dissolved in dimethylformamide (10 mL) and palladium 10% (60 mg, 564 µmol) was added. The mixture was stirred under H_2_ (g) at room temperature for 4 h. After filtration over a pad of celite to remove the palladium, the filtrate was concentrated under reduced pressure. The residue was suspended in water and filtered to give 183 mg of a green powder (Quantitative yield).

^1^H NMR (400 MHz, DMSO-*d_6_*) δ 11.09 (s, 1H), 7.48 (dd, *J* = 8.4, 7.0 Hz, 1H), 7.02 (d, *J* = 7.1 Hz, 2H), 6.53 (s, 2H), 5.05 (dd, *J* = 12.9, 5.4 Hz, 1H), 2.89 (ddd, *J* = 17.4, 14.5, 5.4 Hz, 1H), 2.65–2.52 (m, 2H), 2.03 (ddt, *J* = 10.9, 5.7, 3.2 Hz, 1H).

^13^C NMR (101 MHz, DMSO-*d_6_*) δ 173.3, 170.6, 169.1, 167.9, 147.2, 136.0, 132.5, 122.2, 111.5, 109.0, 49.0, 31.5, 22.6.

4-Azido-2-(2,6-dioxopiperidin-3-yl)isoindoline-1,3-dione (**15**).

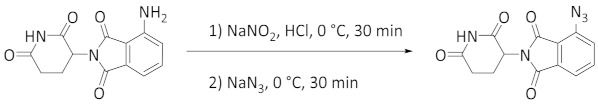



Sodium nitrite (1.03 mL, 2.06 mmol, 2 M in water) was added dropwise at 0 °C to a stirred solution of 4-amino-2-(2,6-dioxopiperidin-3-yl)isoindoline-1,3-dione (470 mg, 1.72 mmol) in concentrated HCl (7 mL). The mixture was placed in an ice bath and stirred for 30 min, then sodium azide (2.36 mL, 2.36 mmol, 1 M in water) was added slowly at 0 °C. The mixture was stirred for an additional 30 min. The precipitate was then filtered off and washed with water to give 470 mg of a pale-yellow solid (Yield 84%).

^1^H NMR (400 MHz, DMSO-*d_6_*) δ 11.14 (s, 1H), 7.89 (dd, *J* = 8.3, 7.2 Hz, 1H), 7.71 (ddd, *J* = 7.2, 4.0, 0.8 Hz, 2H), 5.15 (dd, *J* = 12.8, 5.4 Hz, 1H), 2.90 (ddd, *J* = 16.9, 13.8, 5.4 Hz, 1H), 2.67–2.52 (m, 2H), 2.06 (dtd, *J* = 12.9, 5.2, 2.1 Hz, 1H).

^13^C NMR (101 MHz, DMSO-*d_6_*) δ 173.2, 170.2, 166.9, 165.4, 138.3, 136.8, 133.6, 127.2, 120.3, 120.1, 49.4, 31.4, 22.4

4-(4-(2-Biguanide-ethyl)-1*H*-1,2,3-triazol-1-yl)-2-(2,6-dioxopiperidin-3-yl)isoindoline-1,3-dione formate (**16**).

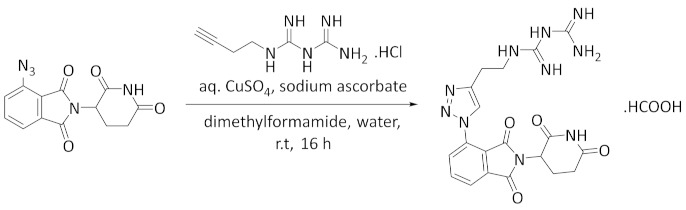



A mixture of but-3-yn-1-biguanide hydrochloride (195 mg, 1.03 mmol) and 4-azido-2-(2,6-dioxopiperidin-3-yl)isoindoline-1,3-dione (237 mg, 792 µmol) was stirred in dimethylformamide (3.60 mL) under a nitrogen atmosphere. Copper(II) sulfate (37.9 mg, 238 µmol) in 0.2 mL of water and (+)-sodium L-ascorbate (157 mg, 792 µmol) in 0.2 mL of water were successively added and the reaction mixture was stirred for 16 h at room temperature. The mixture was diluted in dichloromethane and the precipitate was filtered off and washed with dichloromethane. The solid was then dissolved in methanol and filtered over a pad of celite, then concentrated under reduced pressure to give an orange foam. The crude product was purified by HPLC prep and lyophilized to obtain 50 mg of a white foam (Yield 13%).

^1^H NMR (700 MHz, DMSO-*d_6_*) δ 11.20 (s, 1H), 8.58 (s, 1H, HCOOH), 8.40 (bs, 1H), 8.22–8.12 (m, 1H), 8.10 (d, *J* = 5.5 Hz, 2H), 7.20 (bs, 5H), 5.19 (dd, *J* = 12.9, 5.5 Hz, 1H), 3.46 (t, *J* = 6.8 Hz, 2H), 3.04–2.89 (m, 2H), 2.87 (q, *J* = 7.4, 6.7 Hz, 1H), 2.62 (dt, *J* = 17.1, 3.5 Hz, 1H), 2.56–2.51 (m, 1H), 2.12–1.97 (m, 1H)

^13^C NMR (176 MHz, DMSO-*d_6_*) δ 173.3, 170.1, 166.7, 166.4, 165.1, 144.8, 137.0, 133.5, 133.3, 131.3, 125.6, 124.5, 122.8, 49.6, 31.3, 22.3.

LC-MS (ESI): (90% H_2_O to 50% MeOH in 15 min, DAD 254 nm), t_R_ = 8.10 min, 85% purity.

HRMS: *m*/*z* [M+H]^+^ calcd for C_19_H_20_N_10_O_4_, 453.17418; found 453.17436; [M+Na]^+^ calcd 475.15612; found 475.16078.

2-(2-(2-Azidoethoxy)ethoxy)ethan-1-ol (**17**).

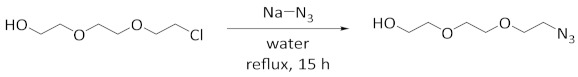



2-(2-(2-Chloroethoxy)-ethoxy)ethanol (1.72 mL, 11.9 mmol) was added to a solution of sodium azide (1.54 g, 23.7 mmol) in water (80.0 mL) and the mixture was heated to reflux overnight. The solution was allowed to return to room temperature and was then extracted three times with dichloromethane. Combined organic layers were washed with brine, dried over MgSO_4_, and concentrated under reduced pressure to obtain 2.02 g of a colorless liquid (Yield 97%).

^1^H NMR (400 MHz, chloroform-*d*) δ 3.80–3.73 (m, 2H), 3.73–3.67 (m, 6H), 3.67–3.62 (m, 2H), 3.42 (t, *J* = 5.0 Hz, 2H), 2.27 (s, 1H).

^13^C NMR (101 MHz, chloroform-*d*) δ 72.5, 70.7, 70.4, 70.0, 61.7, 50.7.

2-(2-(2-Azidoethoxy)ethoxy)ethyl methanesulfonate (**18**).

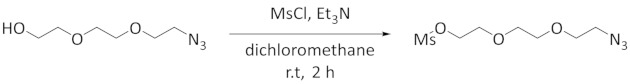



To a solution of 2-(2-(2-azidoethoxy)ethoxy)ethan-1-ol (311 mg, 1.78 mmol) and triethylamine (495 µL, 3.55 mmol) in dichloromethane (8 mL) was added dropwise methanesulfonyl chloride (275 µL, 3.55 mmol) at 0 °C. The reaction mixture was stirred at room temperature for 2 h and diluted with ethyl acetate (6 mL) and saturated aqueous NaHCO_3_ (6 mL). The organic layer was separated and washed once with brine (10 mL). The resulting aqueous layer was extracted once with ethyl acetate (10 mL). The combined organic layers were dried over Na_2_SO_4_ and then concentrated under reduced pressure to obtain 454 mg of a yellow oil (Quantitative yield).

^1^H NMR (400 MHz, chloroform-*d*) δ 4.44–4.37 (m, 2H), 3.85–3.78 (m, 2H), 3.76–3.64 (m, 7H), 3.42 (t, *J* = 5.0 Hz, 2H).

^13^C NMR (101 MHz, chloroform-*d*) δ 70.7, 70.7, 70.1, 69.2, 69.1, 50.7, 37.7, 8.6.

4-(2-(2-(2-Azidoethoxy)ethoxy)ethoxy)-2-(2,6-dioxopiperidin-3-yl)isoindoline-1,3-dione (**19**).

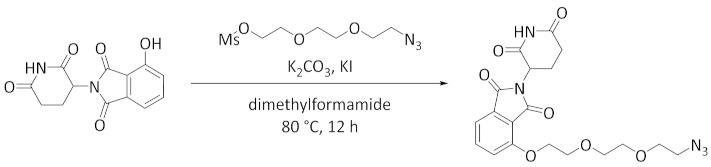



In a round-bottom flask, 2-(2,6-dioxopiperidin-3-yl)-4-hydroxyisoindoline-1,3-dione (577 mg, 2.10 mmol) was dissolved in dimethylformamide (10.2 mL) with potassium iodide (185 mg, 1.12 mmol) and potassium carbonate anhydrous (739 mg, 5.34 mmol). Then, 2-(2-(2-azidoethoxy)ethoxy)ethyl methanesulfonate (645 mg, 2.55 mmol) was then added dropwise to the stirred solution. The mixture was heated to 80 °C for 12 h. Water was added, and the mixture was extracted three times with ethyl acetate. The combined organic layers were washed three times with saturated brine, dried over MgSO_4_, and concentrated under reduced pressure to give 836 mg of a brown oil. This crude product was purified by chromatography on silica gel with eluent 0 to 10% methanol in dichloromethane to obtain 669 mg of an oil that turned into an eggshell solid (Yield 82%).

^1^H NMR (400 MHz, DMSO-*d_6_*) δ 11.11 (s, 1H), 7.82 (dd, *J* = 8.5, 7.3 Hz, 1H), 7.54 (d, *J* = 8.5 Hz, 1H), 7.47 (d, *J* = 7.2 Hz, 1H), 5.10 (dd, *J* = 12.8, 5.4 Hz, 1H), 4.44–4.31 (m, 2H), 3.89–3.79 (m, 2H), 3.72–3.66 (m, 2H), 3.64–3.58 (m, 4H), 3.38 (dd, *J* = 5.6, 4.3 Hz, 2H), 2.97–2.83 (m, 1H), 2.66–2.52 (m, 2H), 2.11–1.98 (m, 1H).

^13^C NMR (101 MHz, DMSO-*d_6_*) δ 173.3, 170.4, 167.3, 165.7, 156.3, 137.5, 133.7, 120.5, 116.8, 115.9, 70.7, 70.2, 69.7, 69.3, 69.2, 50.5, 49.2, 31.4, 22.5.

4-(2-(2-(2-(4-(Biguanidemethyl)-1*H*-1,2,3-triazol-1-yl)ethoxy)ethoxy)ethoxy)-2-(2,6-dioxopiperidin-3-yl)isoindoline-1,3-dione formate (**20**).

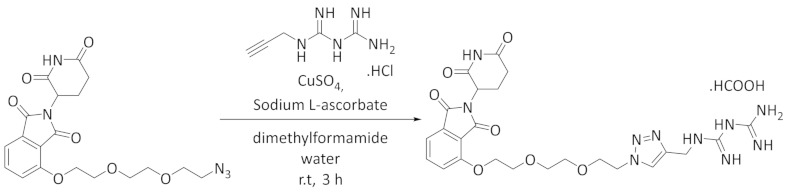



1-Propargyl-biguanide hydrochloride (280 mg, 1.60 mmol) and 4-(2-(2-(2-azidoethoxy)ethoxy)ethoxy)-2-(2,6-dioxopiperidin-3-yl)isoindoline-1,3-dione (462 mg, 1.07 mmol) were dissolved in dimethylformamide (5 mL) and the mixture was flushed with N_2_. A solution of copper(II) sulfate (51.3 mg, 321 µmol) in water (250 µL) and a solution of L-ascorbic acid sodium salt (240 mg, 1.21 mmol) in water (250 µL) were added successively and the resulting solution was stirred for 3 h under N_2_ atmosphere. Dichloromethane was added to the mixture and the precipitate was filtered off, purified by HPLC prep, and lyophilized to obtain 85 mg of a white foam (Yield 14%).

^1^H NMR (500 MHz, DMSO-*d_6_*) δ 11.13 (s, 1H), 8.42 (s, 1H, HCOOH), 8.31–8.05 (m, 1H), 7.99 (bs, 1H), 7.82 (dd, *J* = 8.5, 7.2 Hz, 1H), 7.53 (d, *J* = 8.5 Hz, 1H), 7.47 (d, *J* = 7.2 Hz, 1H), 7.35 (bs, 3H), 7.10 (bs, 2H), 5.09 (dd, *J* = 12.8, 5.5 Hz, 1H), 4.49 (t, *J* = 5.2 Hz, 2H), 4.34 (dd, *J* = 8.2, 3.3 Hz, 4H), 3.82 (t, *J* = 5.2 Hz, 2H), 3.80–3.75 (m, 2H), 3.63 (dd, *J* = 5.9, 3.5 Hz, 2H), 3.56 (dd, *J* = 5.9, 3.4 Hz, 2H), 2.89 (ddd, *J* = 17.0, 13.9, 5.4 Hz, 1H), 2.69–2.52 (m, 2H), 2.04 (dtd, *J* = 10.6, 5.4, 2.7 Hz, 1H).

^13^C NMR (126 MHz, DMSO-*d_6_*) δ 173.3, 170.5, 167.3, 165.8, 156.3, 137.5, 133.7, 123.8, 120.5, 116.7, 115.9, 70.5, 70.0, 69.3, 69.2, 69.1, 49.9, 49.2, 36.9, 31.4, 22.5.

LC-MS (ESI): (95% H_2_O + 0.1% FA to 50% ACN + 0.1% FA in 15 min, +ESI TIC Scan Frag = 70 V), t_R_ = 5.121 min, 100% purity.

HRMS: *m*/*z* [M+H]^+^ calcd for C_24_H_30_N_10_O_7_, 571.23717; found 571.24013.

*tert*-Butyl (4-bromobenzyl)carbamate (**21**).

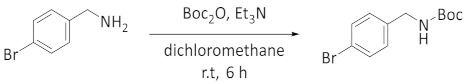



4-Bromobenzylamine (2.33 mL, 18.4 mmol) was dissolved in dichloromethane (35 mL) and placed in an ice bath. Di-*tert*-butyl dicarbonate (6.29 mL, 27.4 mmol) and triethylamine (5.09 mL, 36.5 mmol) were added slowly at 0 °C and the mixture was stirred at room temperature for 6 h. The organic mixture was washed twice with water and once with brine, then dried over MgSO_4_ and concentrated under reduced pressure to give a pale orange oil. Hexanes were added to enhance the precipitation and the mixture was concentrated under reduced pressure. The white solid was washed with hexanes and the precipitate was dried under reduced pressure to obtain 3.20 g of a white powder (Yield 61%).

^1^H NMR (400 MHz, DMSO-*d_6_*) δ 7.51 (d, *J* = 8.3 Hz, 2H), 7.40 (t, *J* = 6.3 Hz, 1H), 7.20 (d, *J* = 8.4 Hz, 2H), 4.09 (d, *J* = 6.2 Hz, 2H), 1.39 (s, 9H).

^13^C NMR (101 MHz, DMSO-*d_6_*) δ 156.2, 140.2, 131.6, 129.6, 120.1, 78.4, 43.3, 28.7

*tert*-Butyl (4-(4-methylthiazol-5-yl)benzyl)carbamate (**22**).

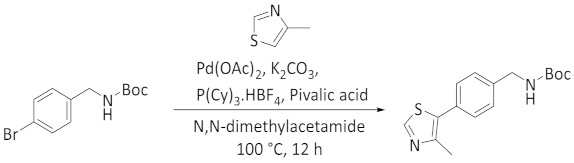



*tert*-Butyl (4-bromobenzyl)carbamate (2.63 g, 9.19 mmol), pivalic acid (282 mg, 2.76 mmol), tricyclohexylphosphine tetrafluoroborate (135 mg, 368 µmol), palladium(II) acetate (54.7 mg, 244 µmol), and potassium carbonate anhydrous (1.91 g, 13.8 mmol) were dissolved in *N,N*-dimethylacetamide (12.3 mL) in a sealed tube and 4-methylthiazole (1.25 mL, 13.8 mmol) was added. The tube was sealed and flushed with N_2_ for 5 min then heated to 100 °C for 12 h. Water was added, and the mixture was then extracted three times with diethyl ether. Combined organic layers were washed with brine, dried over MgSO_4_, filtered over celite and concentrated under reduced pressure to give a yellow solid. The crude product was purified by chromatography on silica gel with gradient 0 to 60% ethyl acetate in hexanes to obtain 2.30 g of a yellow powder (Yield 82%).

^1^H NMR (400 MHz, DMSO-*d_6_*) δ 8.99 (s, 1H), 7.49–7.42 (m, 3H), 7.34 (d, *J* = 8.2 Hz, 2H), 4.18 (d, *J* = 6.2 Hz, 2H), 2.46 (s, 3H), 1.42 (s, 9H).

^13^C NMR (101 MHz, DMSO-*d_6_*) δ 156.3, 151.9, 148.3, 140.6, 131.6, 130.3, 129.3, 127.9, 78.3, 43.5, 28.7, 16.4.

(4-(4-Methylthiazol-5-yl)phenyl)methanamine hydrochloride (**23**).

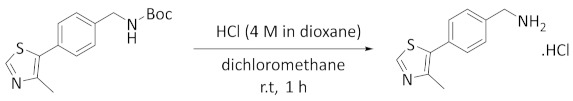



*tert*-Butyl (4-(4-methylthiazol-5-yl)benzyl)carbamate (2.22 g, 7.28 mmol) was dissolved in dichloromethane (10 mL). Hydrochloric acid (18.2 mL, 72.8 mmol, 4 M in dioxane) was added slowly and the mixture was stirred at room temperature for 2 h. The mixture was concentrated under reduced pressure to obtain the hydrochloric salt as a yellow powder (Quantitative yield). No further purification was required.

^1^H NMR (400 MHz, DMSO-*d_6_*) δ 9.22 (s, 1H), 8.71 (s, 3H), 7.65 (d, *J* = 7.8 Hz, 2H), 7.56 (d, *J* = 7.9 Hz, 2H), 4.06 (q, *J* = 5.8 Hz, 2H).

^13^C NMR (101 MHz, DMSO-*d_6_*) δ 153.1, 147.5, 134.7, 131.8, 131.5, 130.1, 129.5, 42.2, 16.0.

*tert*-Butyl(2R)-4-hydroxy-2-((4-(4-methylthiazol-5-yl)benzyl)carbamoyl)pyrrolidine-1-carboxylate (**24**).

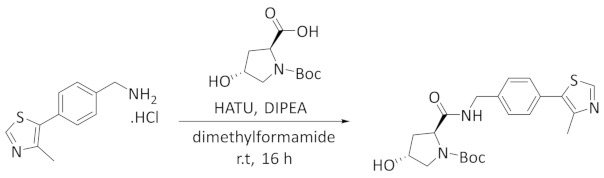



(2R,3R)-1-(*tert*-Butoxycarbonyl)-4-hydroxypyrrolidine-2-carboxylic acid (2.90 g, 12.5 mmol) and HATU (4.77 g, 12.5 mmol) were dissolved in dimethylformamide (50 mL). *N,N*-diisopropylethylamine (4.00 mL, 23.0 mmol) was added and the mixture was stirred at room temperature for 15 min. A solution of (4-(4-methylthiazol-5-yl)phenyl)methanamine hydrochloride (2.00 g, 8.31 mmol) in dimethylformamide (2 mL) was added and the mixture was stirred at room temperature for 16 h. Dichloromethane was added. The solution was washed successively with H_2_O, saturated aqueous NaHCO_3_, aqueous HCl (0.1 M), and brine. The organic layer was dried over MgSO_4_ and concentrated under reduced pressure to give a yellow oil. The crude product was purified by chromatography on silica gel with eluent 0 to 10% methanol in dichloromethane to obtain 1.60 g of a yellow powder (Yield 46%).

^1^H NMR (400 MHz, DMSO-*d_6_*) δ 9.00 (s, 1H), 8.49 (dt, *J* = 10.7, 6.0 Hz, 1H), 7.46–7.36 (m, 4H), 5.02 (dd, *J* = 5.6, 3.5 Hz, 1H), 4.38 (dt, *J* = 14.4, 6.9 Hz, 1H), 4.25 (tt, *J* = 9.0, 4.2 Hz, 2H), 4.20 (d, *J* = 10.8 Hz, 1H), 3.44 (ddd, *J* = 19.5, 11.0, 4.3 Hz, 1H), 3.34–3.25 (m, 1H), 2.45 (s, 3H), 2.16–2.01 (m, 1H), 1.88 (qt, *J* = 7.7, 4.8 Hz, 1H), 1.42 (s, 3H), 1.27 (s, 6H).

^13^C NMR (101 MHz, DMSO-*d_6_*) δ 173.0, 154.0, 152.0, 148.3, 139.9, 131.6, 130.4, 129.3, 128.6, 79.0, 68.3, 59.4, 55.4, 55.3, 42.3, 28.4, 16.4.

(2S)-4-Hydroxy-*N*-(4-(4-methylthiazol-5-yl)benzyl)pyrrolidine-2-carboxamide hydrochloride (**25**).

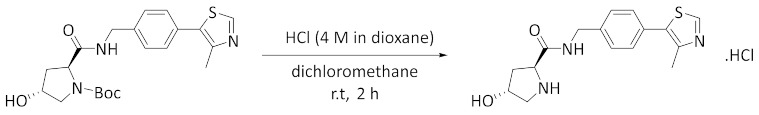



(9*H*-fluoren-9-yl)methyl (2S)-4-hydroxy-2-((4-(4-methylthiazol-5-yl) benzyl) carbamoyl) pyrrolidine-1-carboxylate (1.59 g, 3.80 mmol) was dissolved in dichloromethane (5 mL). Hydrochloric acid (9.50 mL, 38.0 mmol, 4 M in dioxane) was added and the mixture was stirred at room temperature for 2 h. The precipitate was filtered off and washed with dichloromethane to obtain a yellow powder (Quantitative yield).

^1^H NMR (400 MHz, DMSO-*d_6_*) δ 10.25 (q, *J* = 5.1, 4.3 Hz, 1H), 9.37 (t, *J* = 5.9 Hz, 1H), 9.21 (s, 1H), 8.70 (s, 1H), 7.50 (d, *J* = 8.2 Hz, 2H), 7.41 (d, *J* = 8.3 Hz, 2H), 4.43 (m, 4H), 3.45–3.24 (m, 1H), 3.10 (dd, *J* = 12.8, 6.1 Hz, 1H), 2.48 (s, 3H), 2.40–2.32 (m, 1H), 1.93 (ddd, *J* = 13.1, 10.8, 4.4 Hz, 1H).

^13^C NMR (101 MHz, DMSO-*d_6_*) δ 168.5, 152.8, 147.2, 139.2, 132.1, 130.1, 129.5, 128.4, 69.5, 58.5, 53.7, 42.5, 39.2, 15.9.

*tert*-Butyl (1-((2R)-4-hydroxy-2-((4-(4-methylthiazol-5-yl) benzyl) carbamoyl) pyrrolidin-1-yl)-3,3-dimethyl-1-oxobutan-2-yl)carbamate (**26**).

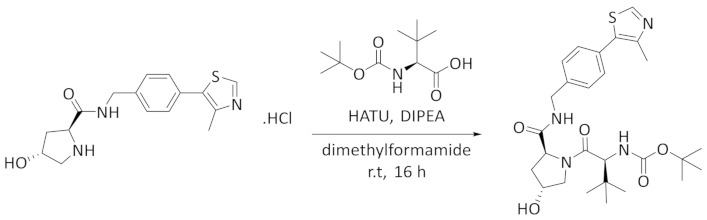



HATU (418 mg, 1.10 mmol) and (S)-2-((*tert*-butoxycarbonyl)amino)-3,3-dimethylbutanoic acid (254 mg, 1.10 mmol) were dissolved in dimethylformamide (5 mL) and *N,N*-diisopropylethylamine (0.5 mL, 2.87 mmol) was added. The mixture was stirred for 5 min. (2S)-4-hydroxy-*N*-(4-(4-methylthiazol-5-yl)benzyl)pyrrolidine-2-carboxamide (324 mg, 0.92 mmol) was dissolved in dimethylformamide (2 mL) and added dropwise to the stirred solution. The mixture was stirred at room temperature for 16 h. Dichloromethane was added, and the mixture was then washed twice with NaHCO_3_, twice with aqueous HCl (0.1 M), once with aqueous LiCl, and finally with brine. The organic layer was dried over MgSO_4_ and concentrated under reduced pressure and purified by chromatography on silica gel with eluent 0 to 10% methanol in dichloromethane to obtain 398 mg of a yellow powder (Yield 82%).

^1^H NMR (500 MHz, DMSO-*d*_6_) δ 8.99 (s, 1H), 8.58 (t, *J* = 6.1 Hz, 1H), 7.43–7.37 (m, 4H), 6.47 (d, *J* = 9.3 Hz, 1H), 5.15 (d, *J* = 3.5 Hz, 1H), 4.48–4.38 (m, 2H), 4.24 (dd, *J* = 15.8, 5.6 Hz, 1H), 4.10 (q, *J* = 5.2 Hz, 1H), 3.67 (m, 1H), 2.45 (s, 3H), 2.10–1.97 (m, 1H), 1.91 (ddd, *J* = 13.1, 8.6, 4.6 Hz, 1H), 1.39 (s, 9H), 0.94 (s, 9H).

^13^C NMR (126 MHz, DMSO-*d_6_*) δ 172.4, 170.3, 155.8, 151.9, 148.2, 139.9, 131.6, 130.1, 129.2, 127.9, 78.5, 69.4, 59.2, 56.8, 49.1, 42.1, 38.4, 35.9, 28.7, 26.8, 16.4.

(2R)-1-(2-Amino-3,3-dimethylbutanoyl)-4-hydroxy-*N*-(4-(4-methylthiazol-5-yl)benzyl) pyrrolidine-2-carboxamide hydrochloride (**27**).

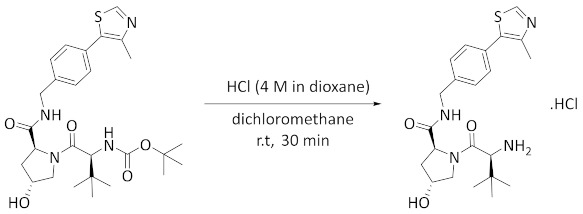



*tert*-Butyl(1-((2R)-4-hydroxy-2-((4-(4-methylthiazol-5-yl)benzyl)carbamoyl)pyrrolidin-1-yl)-3,3-dimethyl-1-oxobutan-2-yl)carbamate (162 mg, 305 µmol) was dissolved in dichloromethane (1.5 mL). Hydrochloric acid (380 µL, 1.5 mmol, 4 M in dioxane) was added and the mixture was stirred at room temperature for 30 min. The precipitate was filtered off and washed with dichloromethane to obtain 160 mg of a yellow powder (Quantitative yield).

^1^H NMR (400 MHz, DMSO-*d_6_*) δ 9.09 (s, 1H), 8.76 (dd, *J* = 6.6, 5.3 Hz, 1H), 8.32–8.06 (m, 3H), 7.42 (s, 4H), 4.56 (dd, *J* = 9.1, 7.6 Hz, 1H), 4.44 (dd, *J* = 15.9, 6.5 Hz, 1H), 4.39 (s, 1H), 4.25 (dd, *J* = 15.8, 5.5 Hz, 1H), 3.91 (d, *J* = 5.5 Hz, 1H), 3.80 (d, *J* = 11.0 Hz, 1H), 3.55 (d, *J* = 3.8 Hz, 1H), 2.46 (s, 3H), 2.13 (dd, *J* = 12.9, 7.9 Hz, 1H), 1.90 (ddd, *J* = 13.1, 9.1, 4.3 Hz, 1H), 1.04 (s, 9H).

^13^C NMR (101 MHz, DMSO-*d_6_*) δ 172.0, 167.2, 152.3, 147.7, 140.0, 131.9, 129.9, 129.2, 127.9, 69.5, 66.8, 59.5, 58.5, 57.0, 55.4, 42.1, 38.6, 34.9, 26.5, 16.2.

4-((1-((2R)-4-Hydroxy-2-((4-(4-methylthiazol-5-yl)benzyl)carbamoyl)pyrrolidin-1-yl)-3,3-dimethyl-1-oxobutan-2-yl)amino)-4-oxobutanoic acid (**28**).

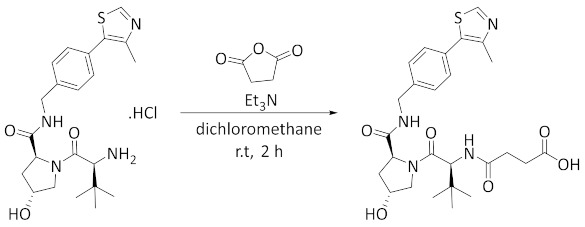



(2R)-1-(2-Amino-3,3-dimethylbutanoyl)-4-hydroxy-*N*-(4-(4-methylthiazol-5-yl)benzyl)pyrrolidine-2-carboxamide hydrochloride (94.0 mg, 218 µmol) and succinic anhydride (50.0 mg, 500 µmol) were dissolved in dichloromethane (3.00 mL). Triethylamine (40 µL, 287 µmol) was added dropwise and the solution was stirred at room temperature for 2 h. The mixture was washed twice with 0.1 M aqueous HCl, once with water, and finally with brine. The organic layer was dried over MgSO_4_ and concentrated under reduced pressure to obtain 116 mg of the crude product. This compound was used without further purification.

*N*^1^-(6-(3-Biguanidehexyl)-*N*^4^-(1-((2S)-4-hydroxy-2-((4-(4-methylthiazol-5-yl)benzyl)carbamoyl) pyrrolidin-1-yl)-3,3-dimethyl-1-oxobutan-2-yl)succinamide formate (**29**).

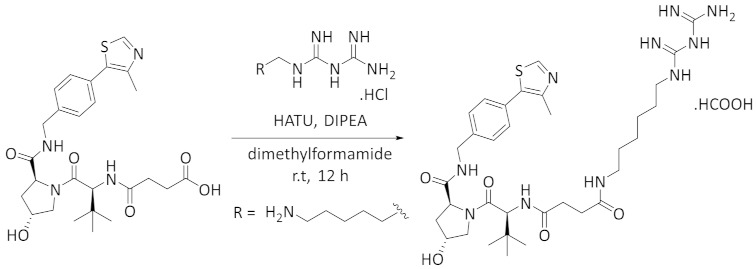



4-((1-((2S)-4-Hydroxy-2-((4-(4-methylthiazol-5-yl)benzyl)carbamoyl)pyrrolidin-1-yl)-3,3-dimethyl-1-oxobutan-2-yl)amino)-4-oxobutanoic acid (116 mg, 219 µmol) and HATU (91.4 mg, 240 µmol) were dissolved in dimethylformamide (1.09 mL) and *N*,*N*-diisopropylethylamine (152 µL, 874 µmol) was then added. The solution was stirred for 10 min and then 1-aminohexylbiguanide hydrochloride (80.2 mg, 339 µmol) was added. The reaction mixture was stirred at room temperature for 12 h. Dichloromethane was added and the precipitate was filtered off, purified by HPLC prep, and lyophilized to give 12 mg of a white cloudy solid (Yield 7%).

^1^H NMR (500 MHz, DMSO-*d_6_*) δ 8.99 (s, 1H), 8.59 (t, *J* = 6.1 Hz, 1H), 8.35 (s, 1H, HCOOH), 7.92 (d, *J* = 9.5 Hz, 1H), 7.85–7.77 (m, 1H), 7.41 (m, 4H), 6.97 (bs, 4H), 4.53 (d, *J* = 9.3 Hz, 1H), 4.47–4.39 (m, 2H), 4.35 (d, *J* = 4.1 Hz, 1H), 4.23 (dd, *J* = 15.9, 5.5 Hz, 1H), 3.70–3.59 (m, 3H), 3.57 (s, 1H), 3.09–2.97 (m, 4H), 2.45 (s, 3H), 2.40–2.22 (m, 4H), 2.08–2.00 (m, 1H), 1.91 (ddd, *J* = 12.8, 8.6, 4.6 Hz, 1H), 1.47–1.40 (m, 2H), 1.37 (d, *J* = 6.4 Hz, 2H), 1.28–1.23 (m, 4H), 0.94 (s, 9H).

^13^C NMR (126 MHz, DMSO-*d_6_*) δ 172.0, 171.3, 171.2, 170.4, 169.6, 151.5, 147.7, 139.5, 131.2, 129.7, 128.7, 127.4, 68.9, 58.7, 56.4, 56.3, 51.3, 41.7, 38.4, 38.0, 35.4, 31.0, 30.6, 29.8, 29.1, 28.9, 26.4, 26.1, 16.0.

LC-MS (ESI): (95% H_2_O + 0.1% FA to 40% ACN + 0.1% FA in 18 min, +ESI TIC Scan Frag = 150 V), t_R_ = 10.50 min, 79% purity.

HRMS: *m*/*z* [M+H]^+^ calcd for C_34_H_52_N_10_O_5_S, 713.391567; found 713.39488.

2-(2-(2-(2-Azidoethoxy)ethoxy)ethoxy)acetic acid (**30**).

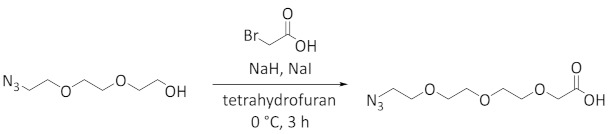



A 60% dispersion of sodium hydride in mineral oil (740 mg, 30.9 mmol) was dissolved in dry tetrahydrofuran (20 mL) then cooled down to 0 °C. Then, 2-[2-(2-Azidoethoxy)ethoxy]ethanol (770 mg, 4.40 mmol) was dissolved in dry tetrahydrofuran (5 mL) and added slowly to the previous mixture under N_2_ at 0 °C. The reaction was stirred for 40 min then cooled on an ice bath followed by the addition of bromoacetic acid (693 µL, 6.59 mmol) and sodium iodide (600 mg, 4.00 mmol) previously dissolved in dry tetrahydrofuran (5 mL). The reaction was stirred for 3 h at room temperature. Water (20 mL) was added and the mixture was stirred overnight. The solution was concentrated in vacuo to remove the tetrahydrofuran and then partitioned between dichloromethane and water. The aqueous layer was washed twice with dichloromethane, acidified with HCl (1 M), and then extracted three times with dichloromethane. Combined organic layers were dried over MgSO_4_, and then filtered and concentrated under reduced pressure to obtain 594 mg of the product (Yield 58%).

^1^H NMR (400 MHz, DMSO-*d*_6_) δ 12.57 (bs, 1H), 4.03 (s, 2H), 3.68–3.50 (m, 10H), 3.44–3.37 (m, 2H).

^13^C NMR (101 MHz, DMSO-*d*_6_) δ 172.1, 70.3, 70.3, 70.22, 70.1, 69.7, 68.0, 50.5.

(2S)-1-(14-Azido-2-(*tert*-butyl)-4-oxo-6,9,12-trioxa-3-azatetradecanoyl)-4-hydroxy-*N*-

(4-(4-methylthiazol-5-yl)benzyl)pyrrolidine-2-carboxamide (**31**).

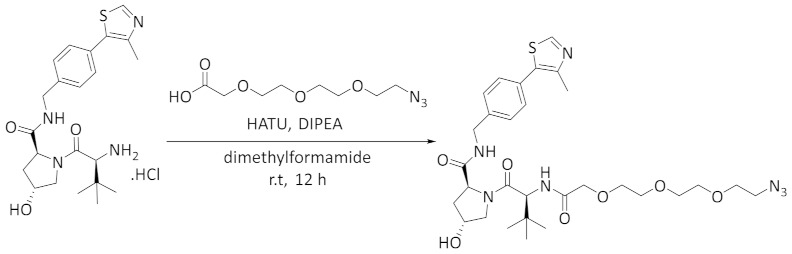



2-(2-(2-(2-Azidoethoxy)ethoxy)ethoxy)acetic acid (188 mg, 804 µmol) and HATU (306 mg, 804 µmol) were dissolved in dimethylformamide (3.09 mL) and *N,N*-diisopropylethylamine (431 µL, 2.48 mmol) was added. The solution was stirred for 5 min and (2R)-1-(2-amino-3,3-dimethylbutanoyl)-4-hydroxy-*N*-(4-(4-methylthiazol-5-yl)benzyl)pyrrolidine-2-carboxamide hydrochloride (289 mg, 619 µmol) was added. The mixture was stirred for 12 h at room temperature. Dichloromethane was added and the solution was washed successively once with water, twice with saturated aqueous NaHCO_3_, three times with aqueous HCl (0.1 M), and finally with brine. The organic layer was dried over MgSO_4_ and concentrated under reduced pressure. The crude product was purified by chromatography on silica gel with eluent 0 to 10% methanol in dichloromethane to obtain 288 mg of the title compound as a yellow oil (Yield 72%).

^1^H NMR (400 MHz, DMSO-*d_6_*) δ 9.00 (s, 1H), 8.60 (t, *J* = 6.0 Hz, 1H), 7.41 (s, 4H), 5.16 (d, *J* = 3.5 Hz, 1H), 4.58 (d, *J* = 9.6 Hz, 1H), 4.48–4.41 (m, 1H), 4.37 (d, *J* = 7.5 Hz, 1H), 4.26 (dd, *J* = 15.7, 5.6 Hz, 1H), 3.98 (s, 2H), 3.64–3.56 (m, 12H), 3.37 (dd, *J* = 5.6, 4.3 Hz, 2H), 2.46 (s, 3H), 2.12–2.00 (m, 1H), 1.91 (ddd, *J* = 13.1, 8.8, 4.5 Hz, 1H), 0.96 (s, 9H)

^13^C NMR (126 MHz, DMSO-*d_6_*) δ 171.8, 169.1, 168.6, 151.5, 147.8, 139.5, 131.1, 129.7, 128.7, 127.5, 70.5, 69.9, 69.7, 69.6, 69.6, 69.3, 68.9, 58.74 56.6, 55.7, 50.0, 41.7, 37.9, 35.7, 26.2, 15.9

(2S)-1-(2-(*tert*-Butyl)-14-(4-((3-biguanidemethyl)-1*H*-1,2,3-triazol-1-yl)-4-oxo-6,9,12-trioxa-3-azatetradecanoyl)-4-hydroxy-*N*-(4-(4-methylthiazol-5-yl)benzyl)pyrrolidine-2-carboxamide formate (**32**)

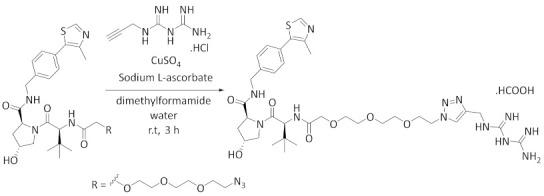



1-Propargylbiguanide hydrochloride (53.8 mg, 307 µmol) and (2R)-1-(14-azido-2-(*tert*-butyl)-4-oxo-6,9,12-trioxa-3-azatetradecanoyl)-4-hydroxy-*N*-(4-(4-methylthiazol-5-yl)benzyl)pyrrolidine-2-carboxamide (132 mg, 204 µmol) were dissolved in dimethylformamide (2.00 mL) and the mixture was flushed with N_2_. A solution of Copper(II) sulfate (9.79 mg, 61.3 µmol) in water (10 µL) and a solution of L-ascorbic acid sodium salt (40.5 mg, 204 µmol) in water (10 µL) were added successively and the resulting solution was stirred at room temperature for 3 h under an N_2_ atmosphere. Dichloromethane was added and the precipitate was filtered off, purified by HPLC prep, and lyophilized to obtain 46 mg of a white cloudy solid (Yield 29%).

^1^H NMR (500 MHz, DMSO-*d_6_*) δ 8.99 (d, *J* = 2.0 Hz, 1H), 8.70–8.52 (m, 1H), 8.06 (bs, 1H), 7.98 (bs, 1H), 7.45 (dd, *J* = 9.6, 3.2 Hz, 1H), 7.40 (bs, 4H), 7.25 (d, *J* = 36.7 Hz, 3H), 7.06 (bs, 2H), 4.58 (d, *J* = 9.6 Hz, 1H), 4.49 (t, *J* = 5.3 Hz, 2H), 4.45 (t, *J* = 8.1 Hz, 2H), 4.36 (t, *J* = 3.2 Hz, 2H), 4.27 (dd, *J* = 15.8, 5.6 Hz, 2H), 3.98 (d, *J* = 2.8 Hz, 2H), 3.80 (t, *J* = 5.3 Hz, 2H), 3.71–3.64 (m, 2H), 3.65–3.57 (m, 4H), 3.57–3.49 (m, 6H), 2.45 (s, 3H), 2.07 (dd, *J* = 12.7, 7.6 Hz, 1H), 1.91 (ddd, *J* = 12.9, 8.8, 4.4 Hz, 1H), 0.95 (s, 9H)

^13^C NMR (126 MHz, DMSO-*d_6_*) δ 172.2, 169.6, 169.1, 152.0, 148.2, 139.9, 131.6, 130.2, 129.2, 127.9, 123.8, 70.9, 70.2, 70.1, 69.3, 69.2, 59.2, 57.1, 56.2, 49.8, 42.1, 38.4, 36.2, 26.7, 16.4

LC-MS (ESI): (90% H_2_O + 0.1% FA to 90% ACN + 0.1% FA in 15 min, DAD 254 nm), t_R_ = 4.32 min, 95% purity

HRMS: *m*/*z* [M+H]^+^ calcd for C_35_H_52_N_12_O_7_S, 785.38754; found, 785.39126

### 3.3. Cell Proliferation Assay and Determination of the EC_50_

Pancreatic cells (KP-4: human pancreatic ductal cell carcinoma cell line derived from human ascites) were obtained from ATCC or Massachusetts General Hospital Center for Molecular Therapeutics (CMT).

KP4 cells were grown in DMEM supplemented with 10% Fetal Bovine Serum (FBS) and Penicillin/Streptomycin at 37% with 5% CO_2_. Cells were seeded at a density of 1 × 103 cells per well in 96 well plates overnight then treated with vehicle and compounds for 72 h. The culture medium was removed. Cells were washed twice with cold PBS and 1% glutaraldehyde was added into the wells for fixation for 10 min. The supernatant was removed. Cells were washed with water, stained with 0.2% crystal violet for 30 min at room temperature, then washed again with water and dried. The adherent crystal violet was solubilized in 10% acetic acid and the absorbance was read at a wavelength of 590 nm for the calculation of the cell viability inhibition rate.

Absorbance measurements for EC50 determination were performed using a SpectraMax 190 microplates reader from Molecular Devices. Resulting data were analyzed with the PRISM software using a fit a curve with non-linear regression (log(inhibitor) vs. response—Variable slope (four parameters)). Statistical analyses were also performed by the PRISM 10 software using a One-way ANOVA test.

### 3.4. Immunoblot Analysis

For the protein degradation assay, cells were seeded at a density of 1 × 105 cells per well in 6-well plates overnight then treated with vehicle and compounds for 24 h. The culture medium was removed. Cells were washed twice with cold PBS and lysed with LAEMMLI 2X (4% SDS, 20% Glycerol, 0.125M Tris-HCl (pH 6.8)) (BioRad, Hercules, CA, USA). Protein concentrations were determined using a Nanodrop 2000c spectrophotometer from Thermo Scientific (Waltham, MA, USA). Equal amounts of protein were separated via sodium dodecyl sulfate-polyacrylamide gel electrophoresis (SDS-PAGE) and transferred to nitrocellulose membranes. Proteins were briefly revealed by Ponceau Red dye and membranes were washed with PBS-Tween 20 (TBST) before blocking with 5% milk proteins for 1 h. Membranes were washed again with TBST and incubated with the primary antibody overnight at 4 °C. After being washed with TBST three times, the membranes were incubated with the secondary antibody for 1 h. After being washed with TBST three times, membranes were visualized by chemiluminescence using a Western Lightning™ Plus Chemiluminescence Reagent. A ChemiDoc MP Imaging System from BioRad (Hercules, CA, USA) was used for imaging and analyzing Western blots. Resulting data were treated with the Image Lab 6.1 software.

Primary Antibodies:

- AMPKα Rabbit pAb #2532 (Cell Signaling, Danvers, MA, USA); 1:1000, 5% BSA, 0.1% Tween^®^ 20 in 1X PBS
- Phospho-AMPKα Rabbit pAb #2531 (Cell Signaling); 1:1000, 5% BSA, 0.1% Tween^®^ 20 in 1X PBS
- GAPDH (basbte3) Goat pAb #NB300-320 (Novus Bio, Toronto, ON, Canada); 1:10,000, 5% BSA, 0.1% Tween^®^ 20 in 1X PBS
- OXPHOS WB cocktail human Mouse mAbs (5) #ab110411 (Abcam, Cambridge, UK); 1:1000, 5% BSA, 0.1% Tween^®^ 20 in 1X PBS

Secondary Antibodies:

- Goat Anti-Rabbit IgG (H + L)-HRP Conjugate #1706515 (BioRad); 1:1000, 5% non fat dry milk, 0.1% Tween^®^ 20 in 1X PBS
- Goat Anti-Mouse IgG (H + L)-HRP Conjugate #1706516 (BioRad); 1:1000, 5% non fat dry milk, 0.1% Tween^®^ 20 in 1X PBS
- Rabbit Anti-Goat IgG (H + L)-HRP Conjugate #1721034 (BioRad); 1:3000, 5% non fat dry milk, 0.1% Tween^®^ 20 in 1X PBS

## 4. Conclusions

The conjugation of the biguanide motif with various ligands of the E3 enzyme, incorporating distinct spacers between the two motifs, yielded a series of synthesized compounds. The impact of these compounds on the proliferation and viability of pancreatic cancer cells (KP4) facilitated the establishment of a correlation between the permeability of the active ingredient and its median inhibitory concentration. This foundational work laid the groundwork for exploring a structure-activity relationship, providing valuable insights into the factors influencing the compounds’ efficacy.

Further investigations into the mechanism of action of these Biguanide-PROTACs revealed that compounds **11** and **12** behaved akin to biguanides concerning the activation of AMPK. However, only compound **12** demonstrated the ability to alter levels of proteins in complexes I and IV. This distinctive observation suggests that the hydrophobic structure of compound **12** might facilitate its accumulation within mitochondria, thereby enhancing its antiproliferative properties. Of note, both compounds are based on the cereblon ligand and cereblon can localize into mitochondria where it can act as a Lon-type protease [42]. This suggests that biguanides-cereblon PROTACs may engage a novel protein inactivation mechanism that does not require ubiquitination and the proteasome. Looking ahead, the development and expansion of the library of Biguanide-PROTACs present an enticing prospect. This extension has the potential to yield new insights into the mechanism of action of PROTAC-biguanides, contributing to a deeper understanding of their targets, while concurrently enabling the creation of more potent anticancer agents. The ongoing exploration of this compound library by exploring broader cell line panels or in vivo models will provide a more comprehensive evaluation of their therapeutic potential and off-target effects, making significant advances in the quest for effective cancer treatments.

## Figures and Tables

**Table 1:** EC_50_ of Biguanide-PROTAC derivatives in KP4 cells after 72 h of treatment (mean ± SD).

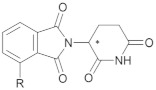			
ID	R ^a^	cLogP ^b^	EC_50_ (mM) ^c^
Met. ^d^	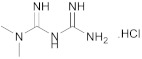	−1.63	0.85 ± 0.06
**8**	-OH	0.4	0.17 ± 0.08
**11**	-O-CH_2_-CONH-(CH_2_)_6_-Big	−0.76	0.34 ± 0.14
**12**	-O-CH_2_-CONH-Ph-C≡C-Bn-Big	1.93	0.15 ± 0.03
**16**	-[1,2,3-triazole]-(CH_2_)_2_-Big	−1.47	0.74 ± 0.10
**20**	-O-((CH_2_)_2_O)_2_-(CH_2_)_2_-[1,2,3-triazole]-CH_2_-Big	−2.06	0.64 ± 0.19
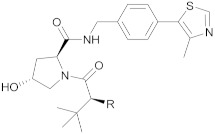			
ID	R ^a^	cLogP ^b^	EC_50_ (mM)
**27**	-NH_2_	2.1	0.34 ± 0.04
**29**	-NH-CO-(CH_2_)_2_-CONH-(CH_2_)_6_-Big	1.4	0.21 ± 0.13
**32**	-NH-CO-CH_2_O-((CH_2_)_2_O)_2_-(CH_2_)_2_-[1,2,3-triazole]-CH_2_-Big	−0.67	0.68 ± 0.06

^a^ Big = Biguanide; ^b^ cLogP were predicted by ChemDraw Professional 22.2 Software (molecules were represented in their monoprotonated state to mimic physiological conditions); ^c^ The data are plotted as mean ± SD of 3 independent replicates; ^d^ Met. = Metformin. (* denotes a chiral center and the fact that the compound was used as a racemic mixture).

## Data Availability

The original contributions presented in the study are included in the article/Supplementary Materials, further inquiries can be directed to the corresponding author/s.

## Supplementary Materials

The following supporting information can be downloaded at: https://www.mdpi.com/article/10.3390/molecules29225329/s1: Figures: ^1^H; ^13^C spectra for compounds 1 to 32.
