# Supplementary material for: Effects of Biguanide-PROTACs in Pancreatic Cancer Cells
Source: Molecules. 2024 Nov 12;29(22):5329. doi: 10.3390/molecules29225329 (PMC11596947; doi:10.3390/molecules29225329)

Supporting information for

Biguanide-PROTACs: Modulating Mitochondrial Proteins in Pancreatic Cancer Cells

Contents of Supporting Information

1. **Characterization data of products 1 to 32……………………………………………………… S2-70**
2. **Statistical analysis…………………………………………………………………………………………….S71**

1. Characterization data of compounds 1 to 32

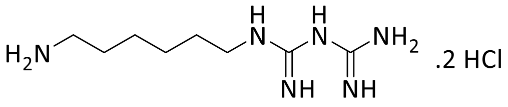

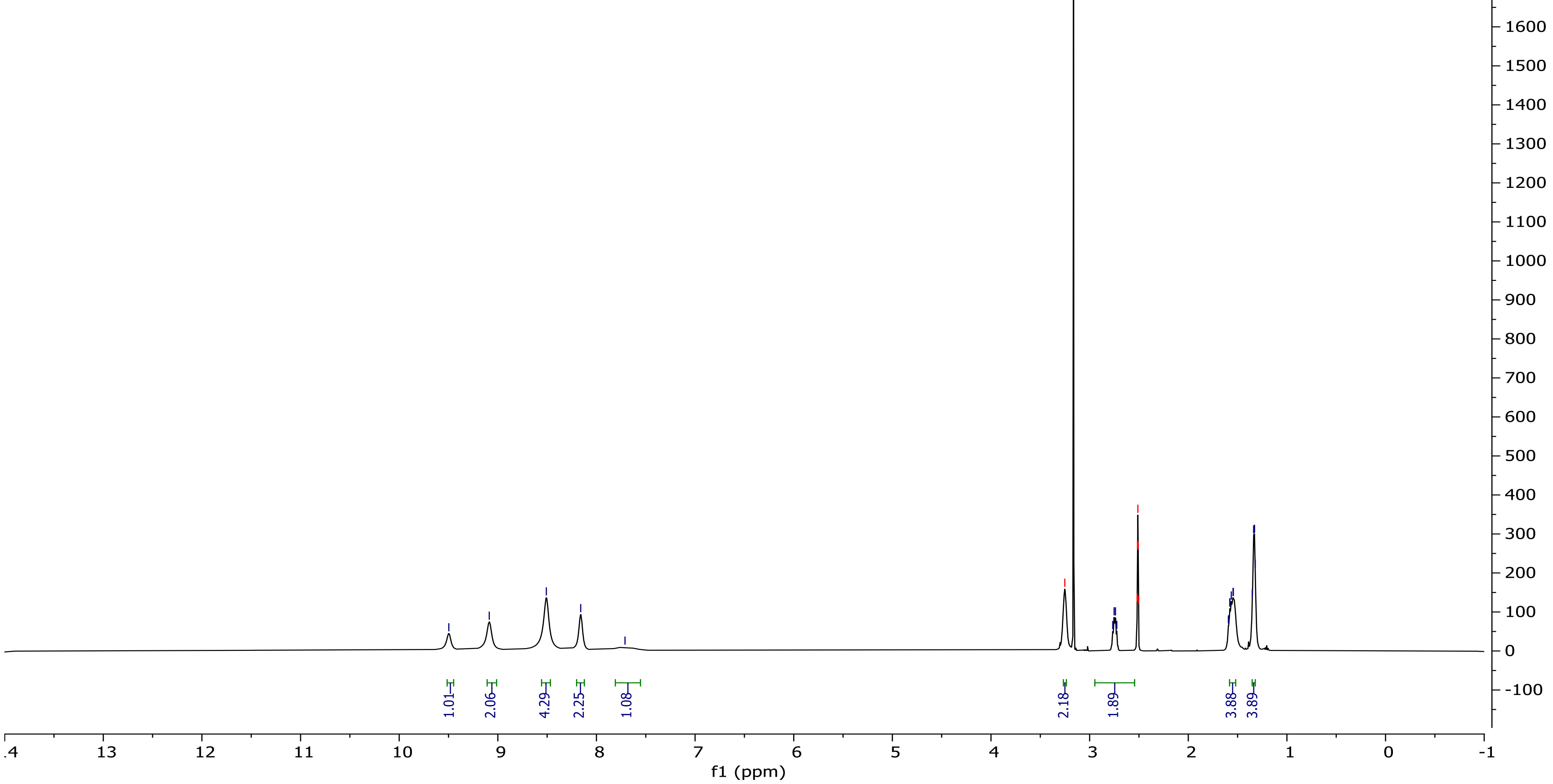

Figure S1. ^{1}H NMR (500 MHz) spectrum of 6-aminohexylbiguanide hydrochloride (1) in DMSO-d_6

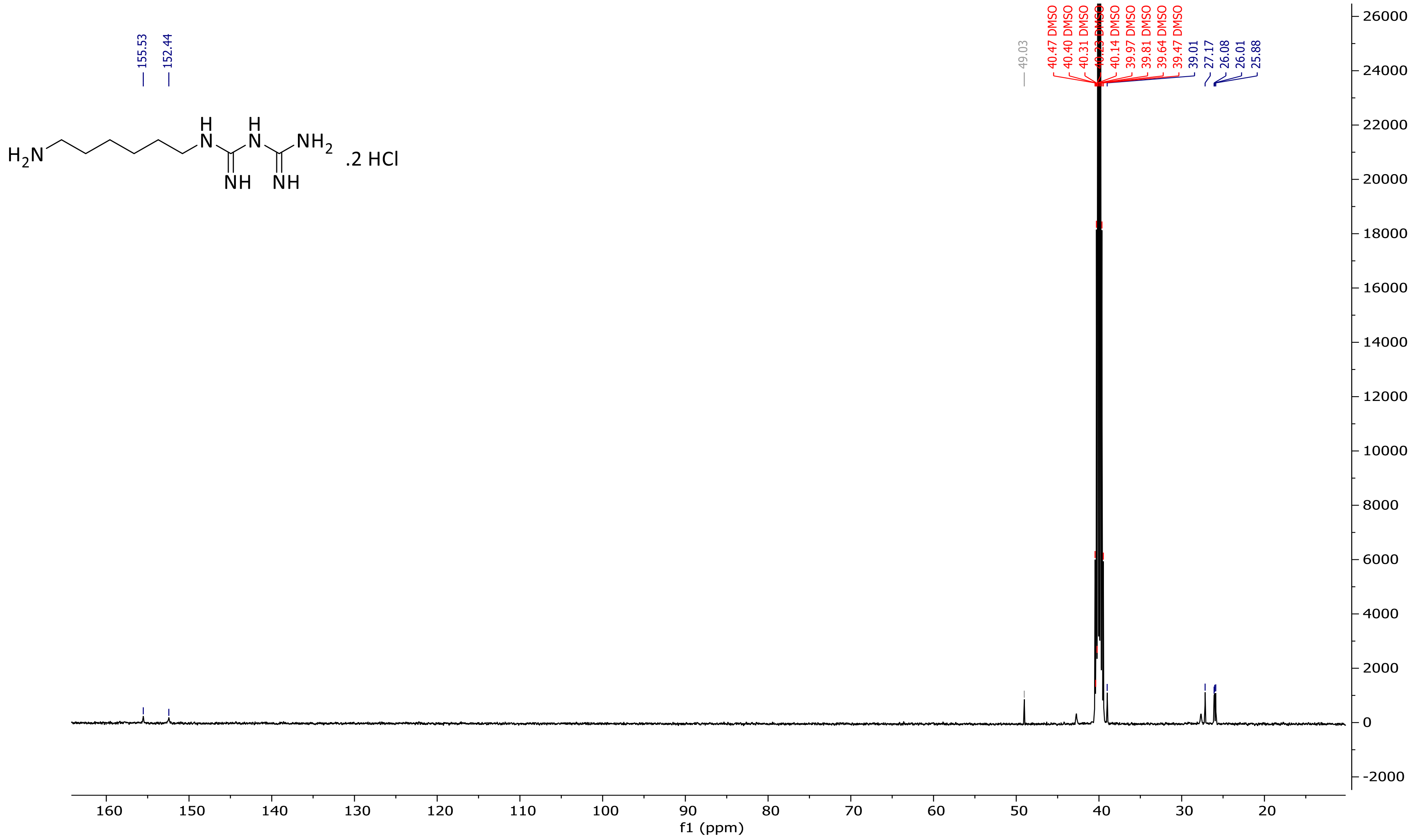

Figure S2. ^{13}C NMR (126 MHz) spectrum of 6-aminohexylbiguanide hydrochloride (1) in DMSO-d_6

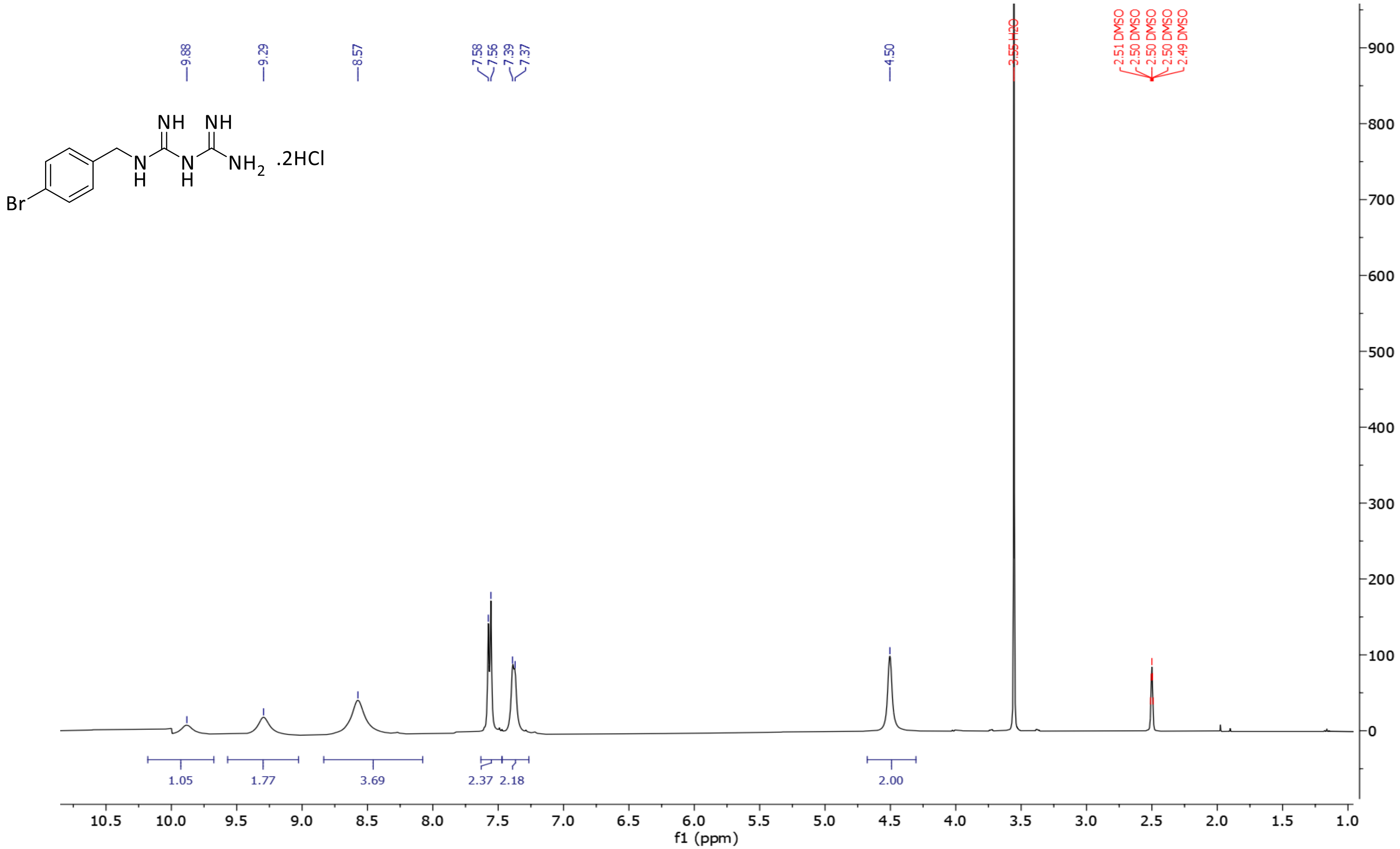

Figure S3. ^{1}H NMR (400 MHz) spectrum of bromobenzylbiguanide hydrochloride (2) in DMSO-d_6

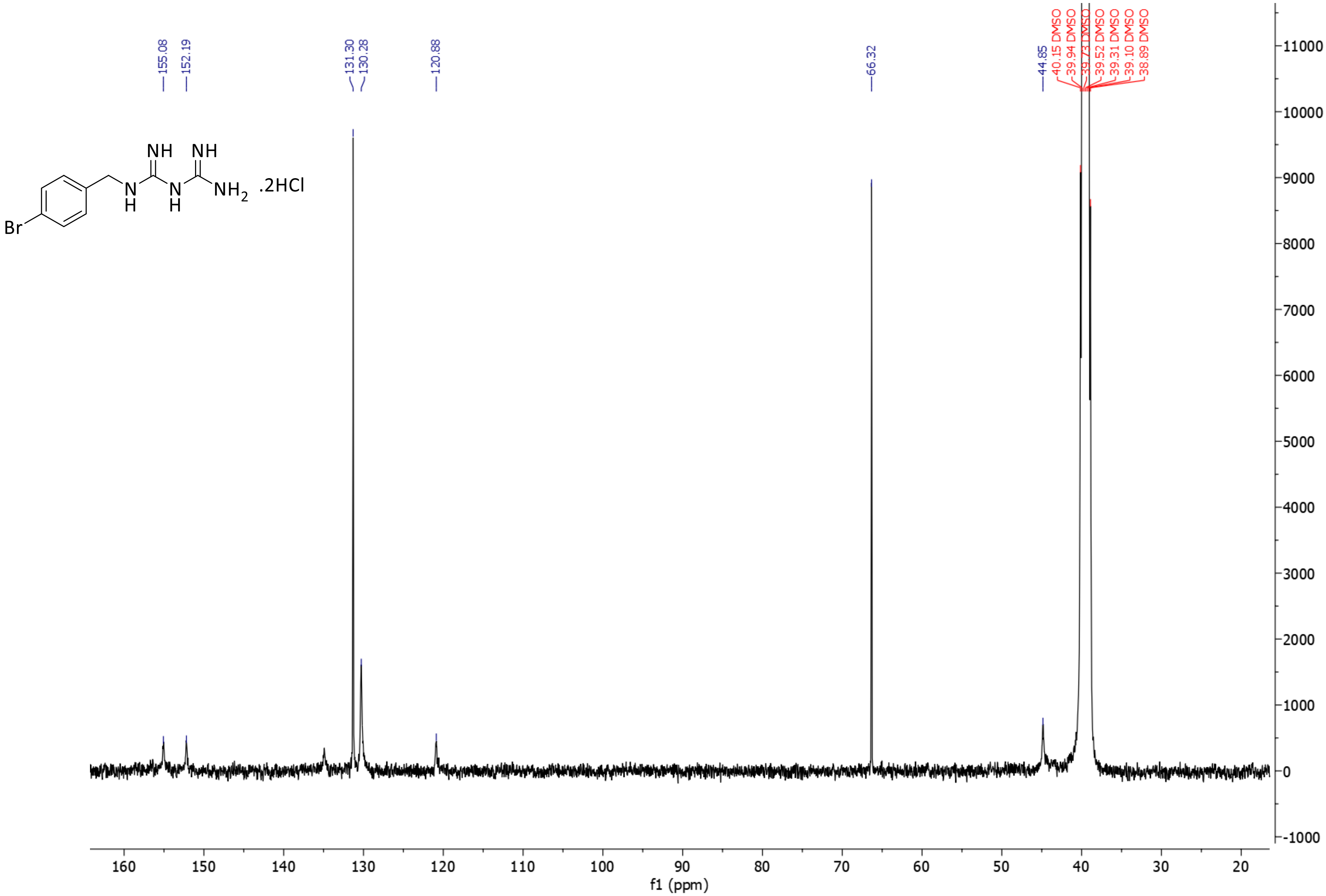

Figure S4. ^{13}C NMR (101 MHz) spectrum of bromobenzylbiguanide hydrochloride (2) in DMSO-d_6

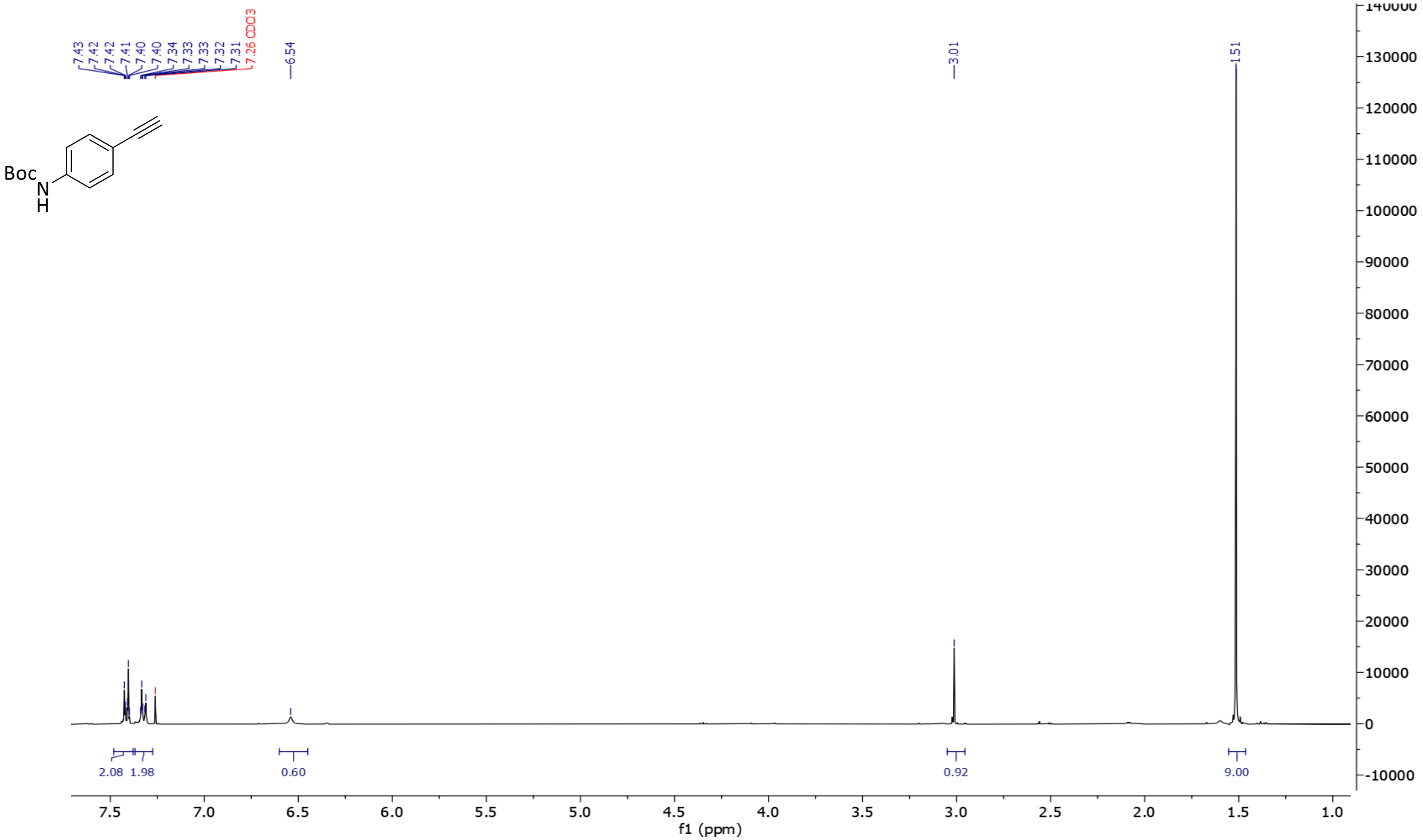

Figure S5. ^{1}H NMR (400 MHz) spectrum of *tert*-butyl (4-ethynylphenyl)carbamate (3) in chloroform-*d*

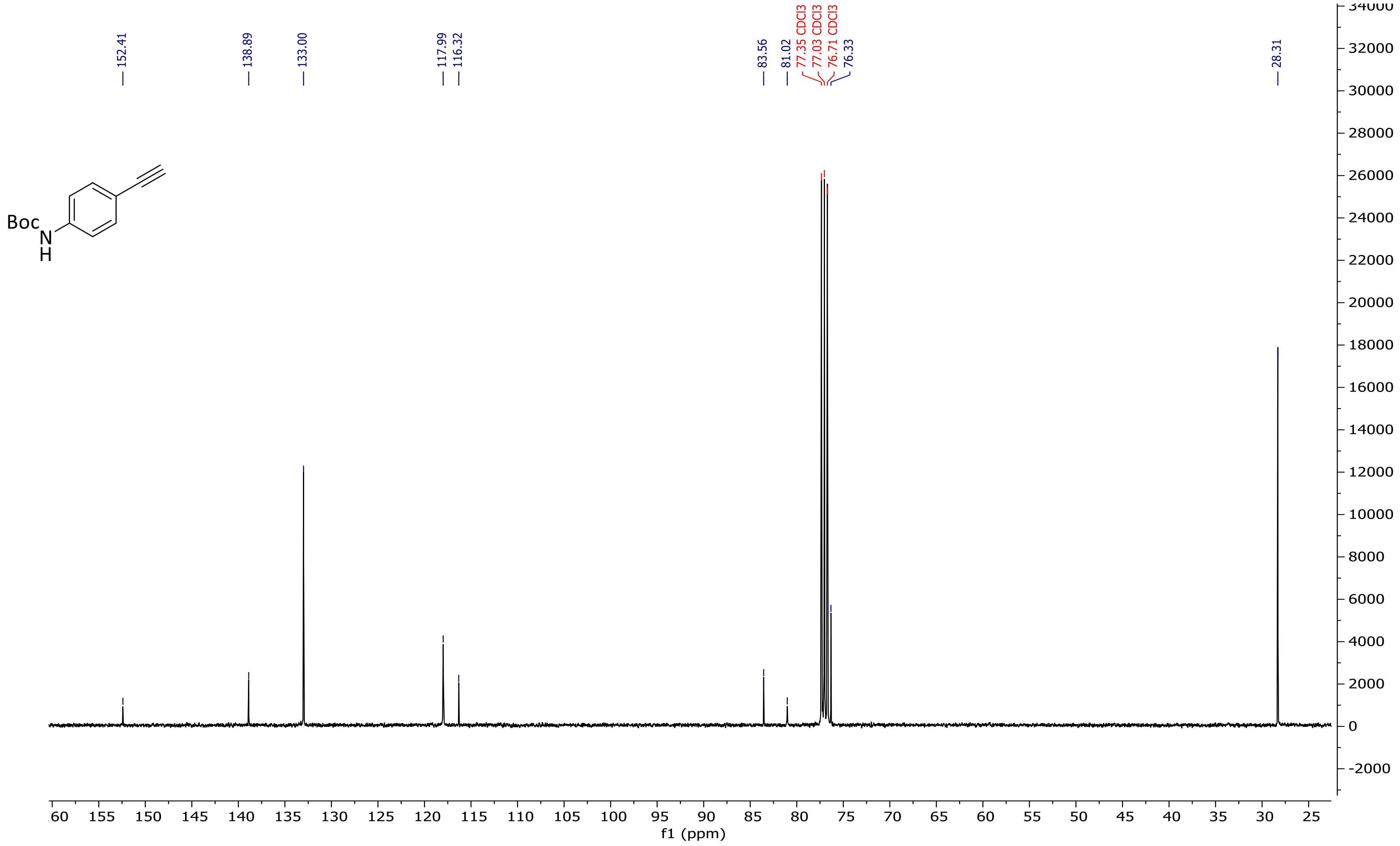

Figure S6. ^{13}C NMR (101 MHz) spectrum of *tert*-butyl (4-ethynylphenyl)carbamate (3) in chloroform-*d*

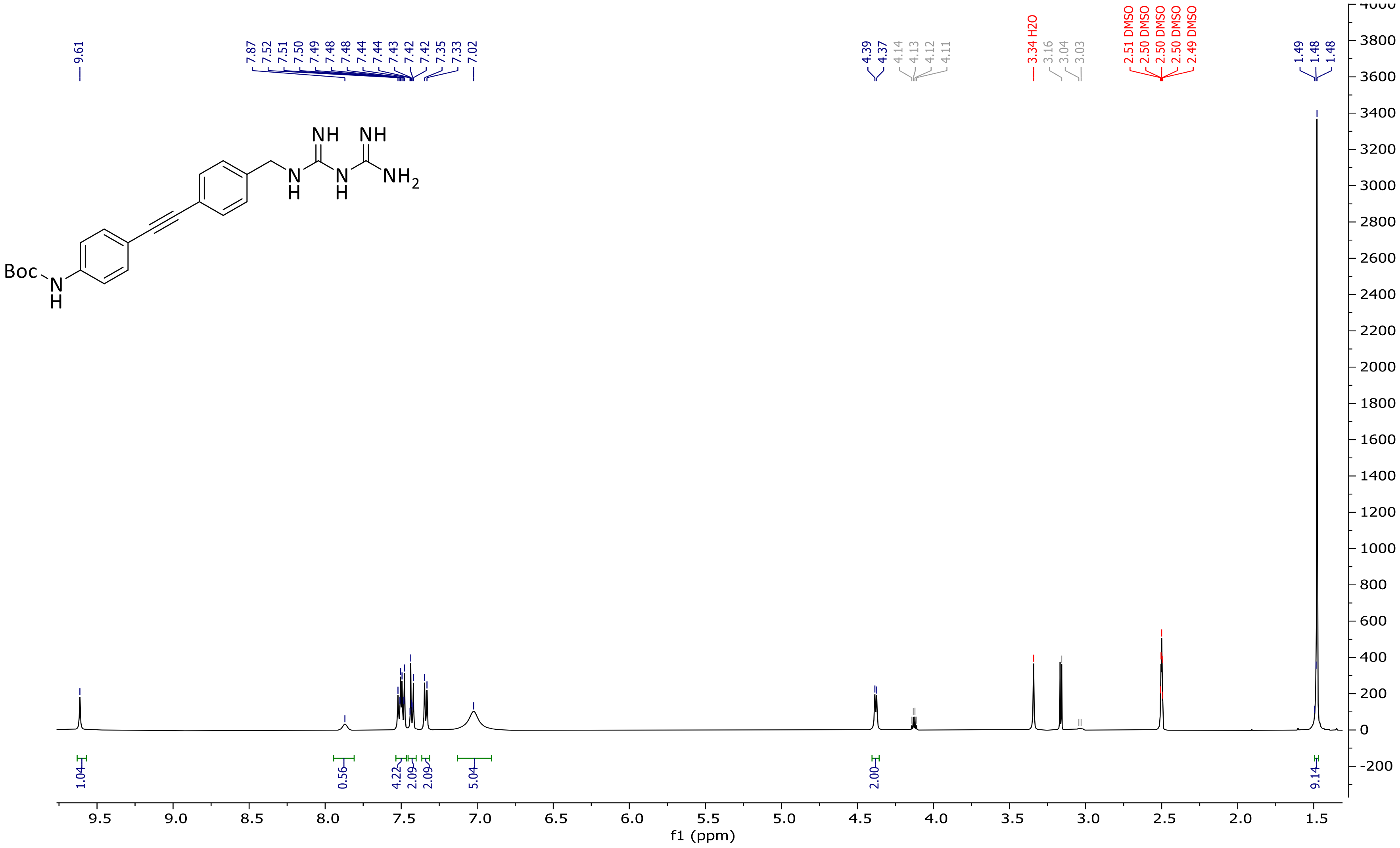

Figure S7. ^{1}H NMR (400 MHz) spectrum of *tert*-butyl(4-((4-((3-carbamimidoylguanidino) methyl)phenyl)ethynyl)phenyl) carbamate (4) in DMSO-d_6

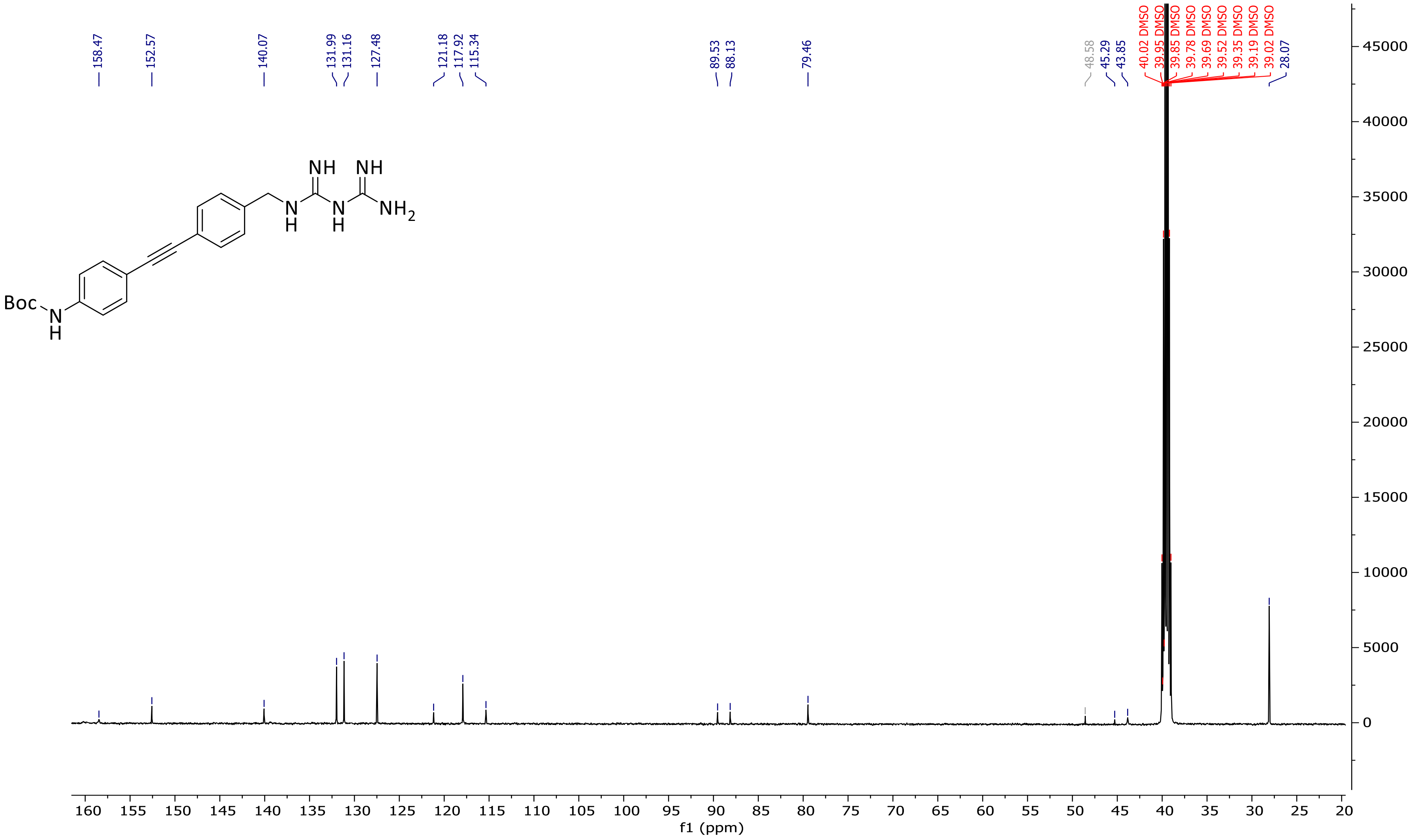

Figure S8. ^{13}C NMR (101 MHz) spectrum of *tert*-butyl(4-((4-((3-carbamimidoylguanidino) methyl) phenyl)ethynyl)phenyl) carbamate (4) in DMSO-d_6

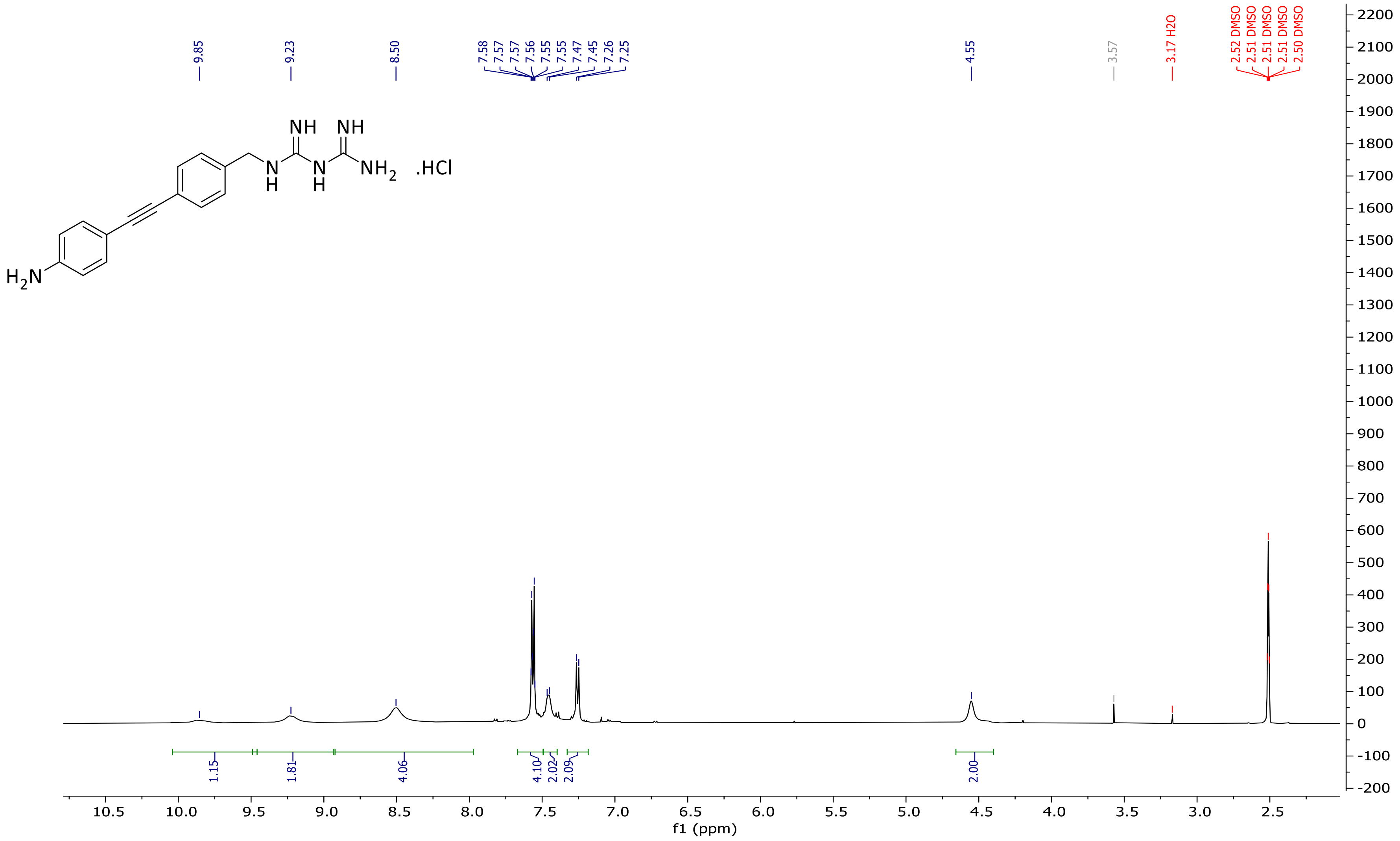

Figure S9. ^{1}H NMR (400 MHz) spectrum of 4-aminophenylethynylbenzylbiguanide hydrochloride (5) in DMSO-d_6

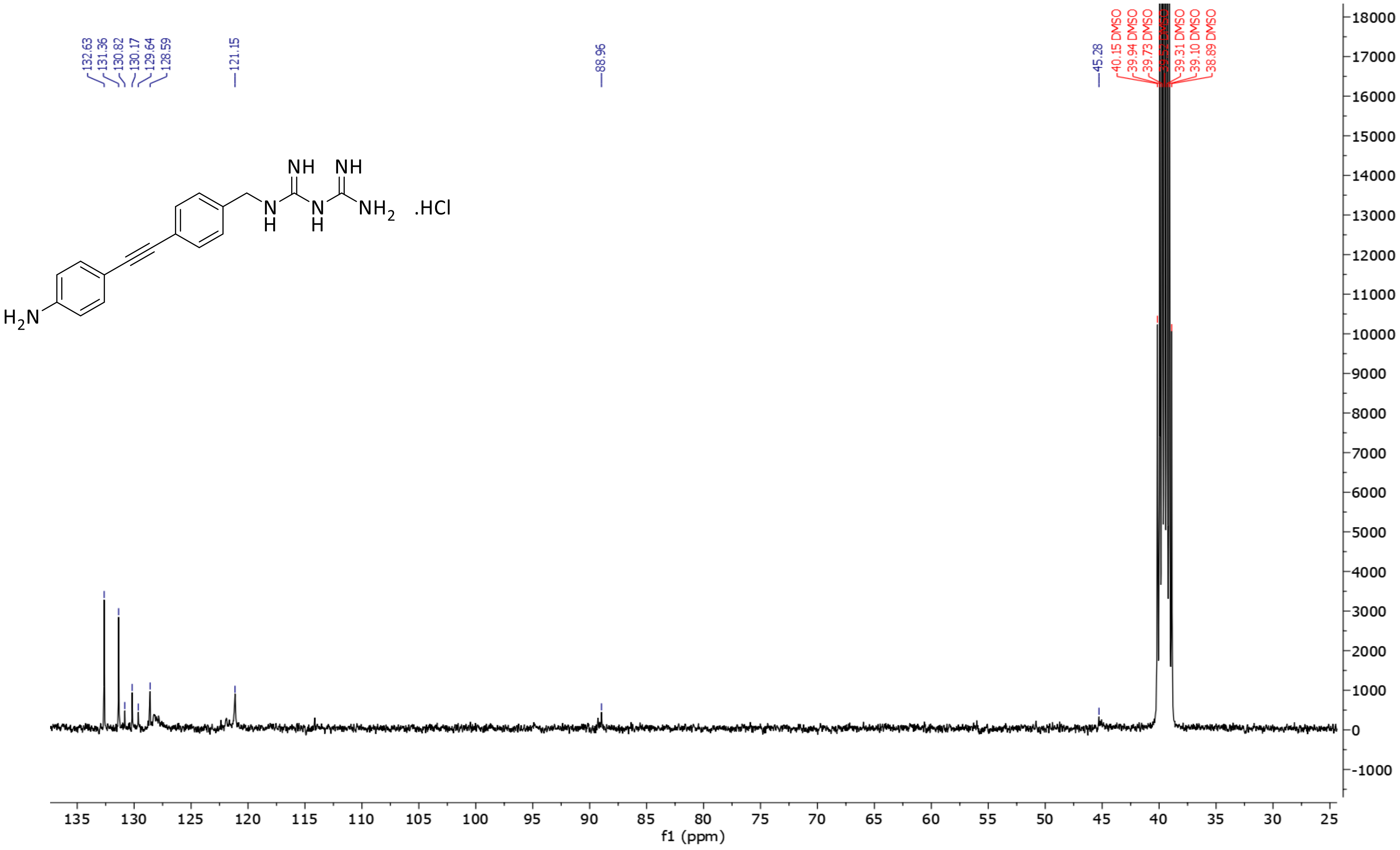

Figure S10. ^{13}C NMR (101 MHz) spectrum of 4-aminophenylethynylbenzylbiguanide hydrochloride (5) in DMSO-d_6

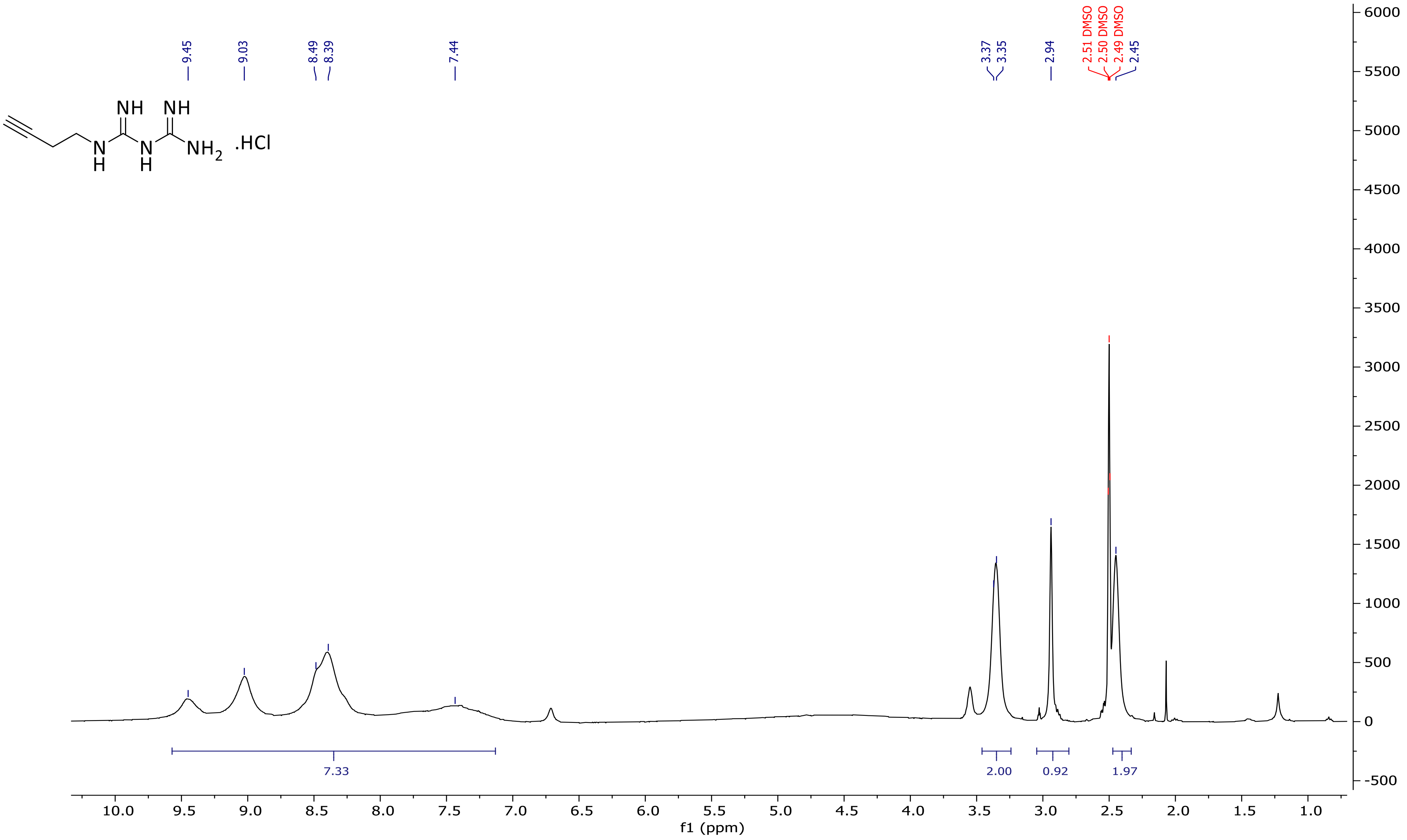

Figure S11. ^{1}H NMR (400 MHz) spectrum of but-3-yn-1-biguanide hydrochloride (6) in DMSO-d_6

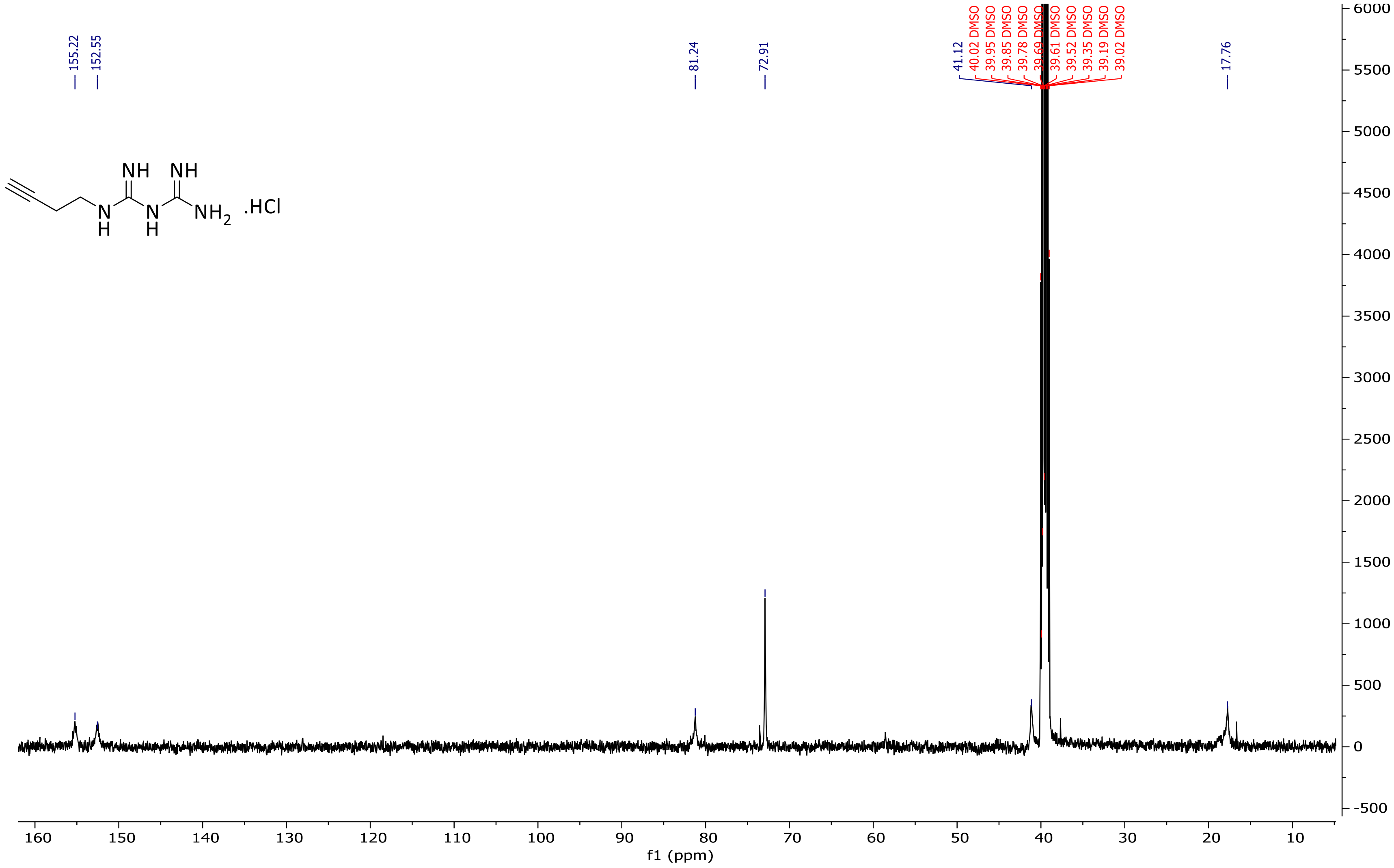

Figure S12. ^{13}C NMR (126 MHz) spectrum of but-3-yn-1-biguanide hydrochloride (6) in DMSO-d_6

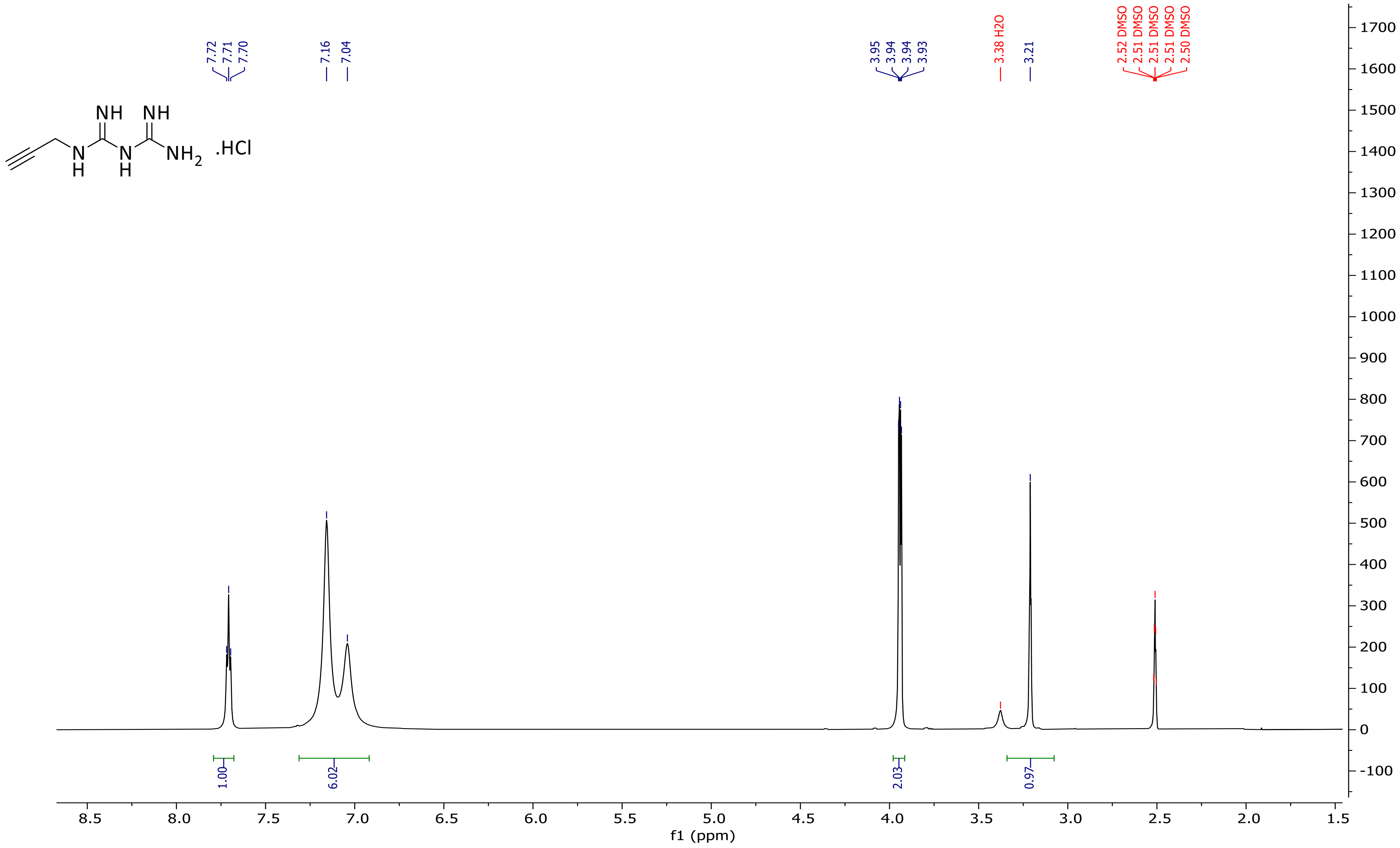

Figure S13. ${}^{1}H$ NMR (500 MHz) spectrum of propargyl-biguanide hydrochloride (**7**) in DMSO-d_6

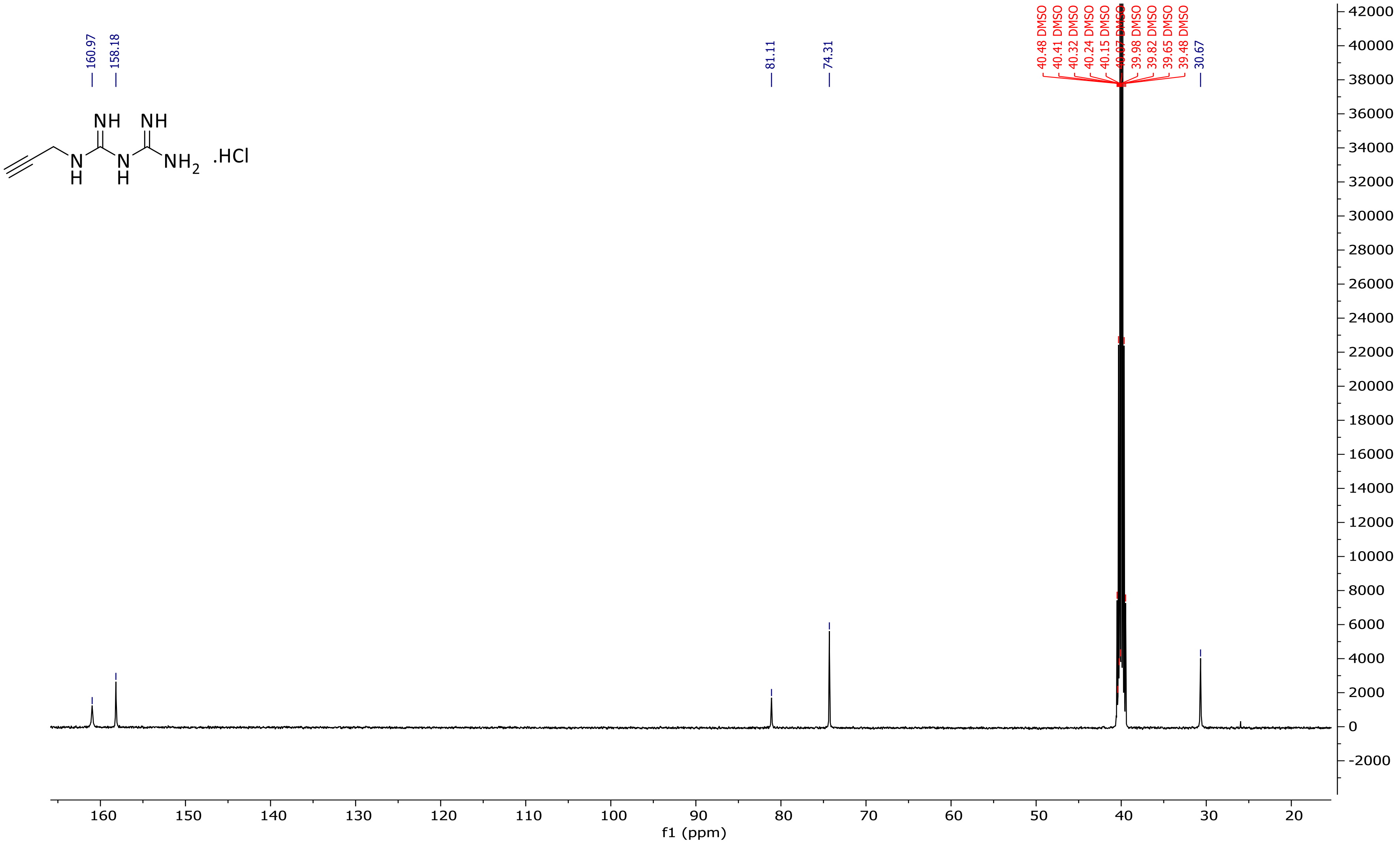

Figure S14. ^{13}C NMR (126 MHz) spectrum of propargyl-biguanide hydrochloride (**7**) in DMSO-d_6

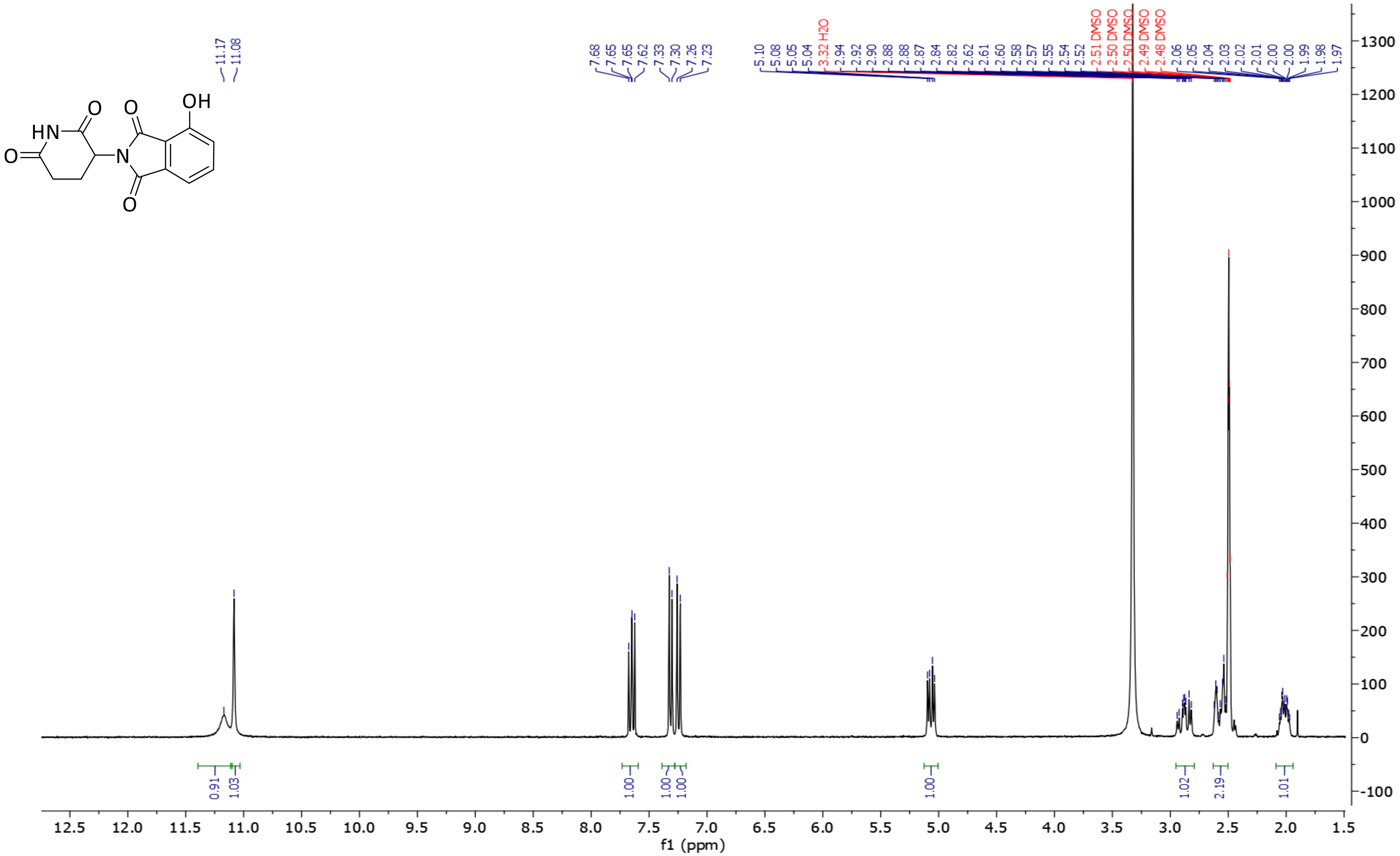

Figure S15. ^{1}H NMR (300 MHz) spectrum of 2-(2,6-dioxopiperidin-3-yl)-4-hydroxyisoindoline-1,3-dione (**8**) in DMSO-d_6

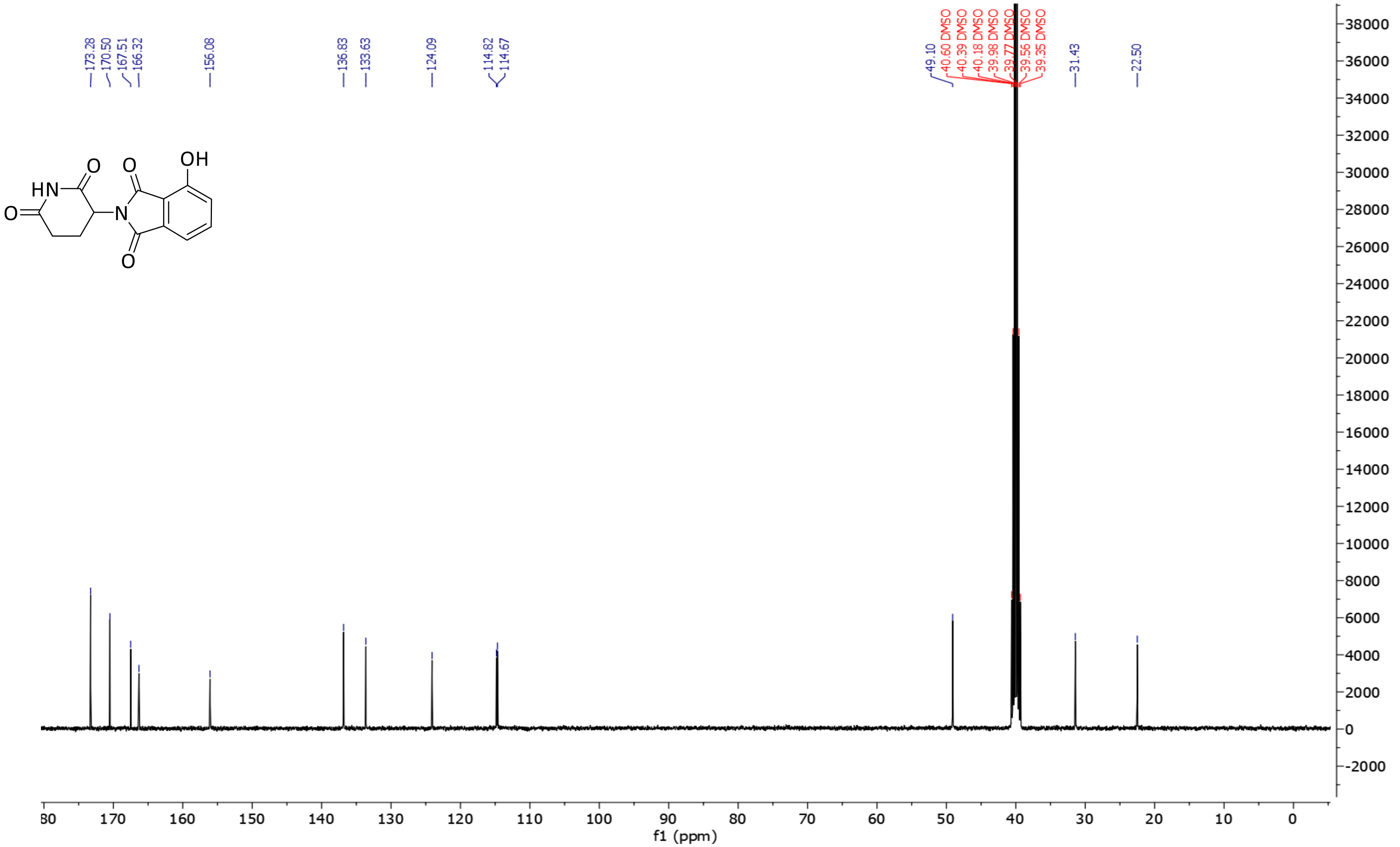

Figure S16. ^{13}C NMR (101 MHz) spectrum of 2-(2,6-dioxopiperidin-3-yl)-4-hydroxyisoindoline-1,3-dione (**8**) in DMSO-d_6

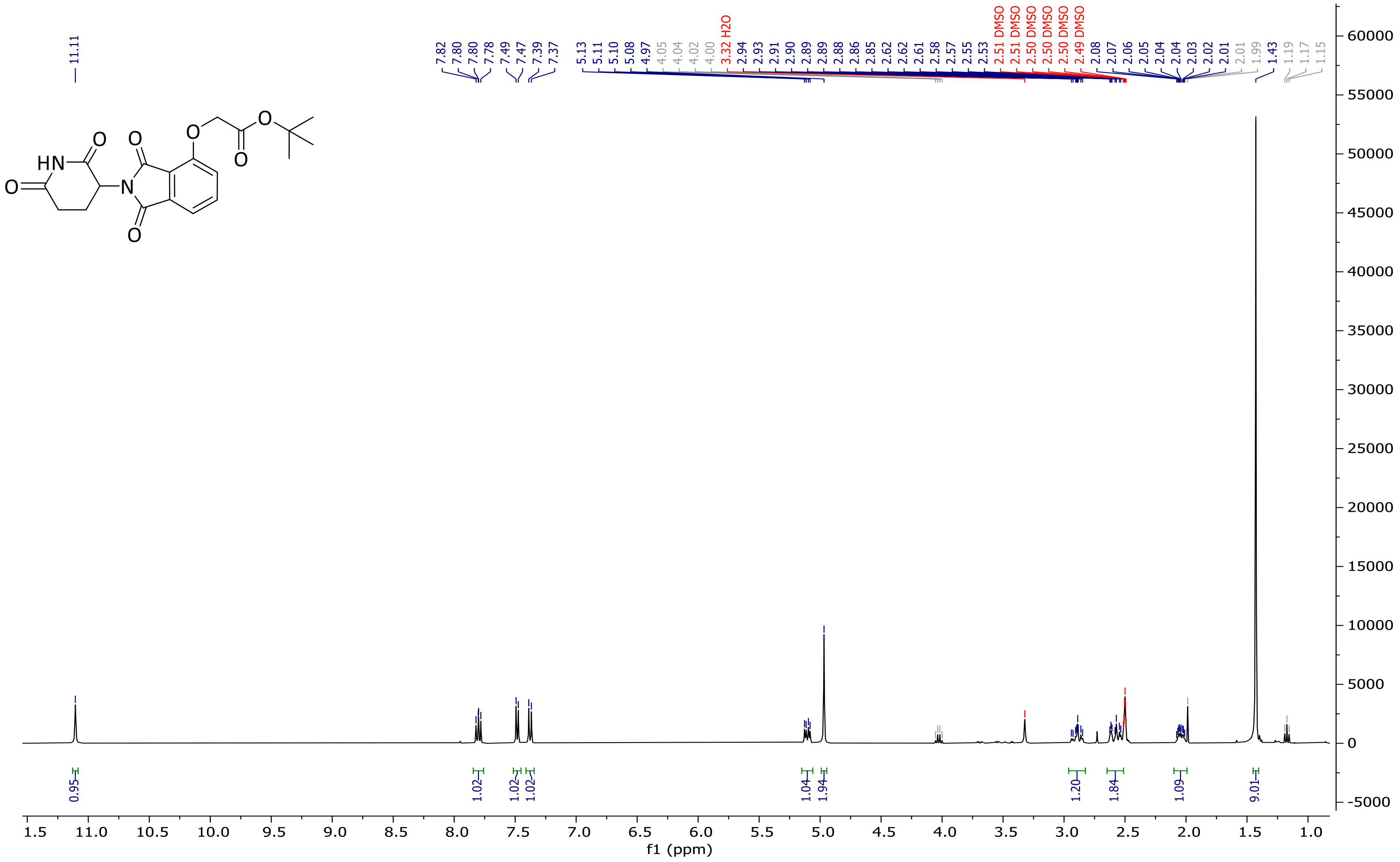

Figure S17. ^{1}H NMR (400 MHz) spectrum of *tert*-butyl 2-((2-(2,6-dioxopiperidin-3-yl)-1,3-dioxoisoindolin-4-yl)oxy)acetate (**9**) in DMSO-d_6

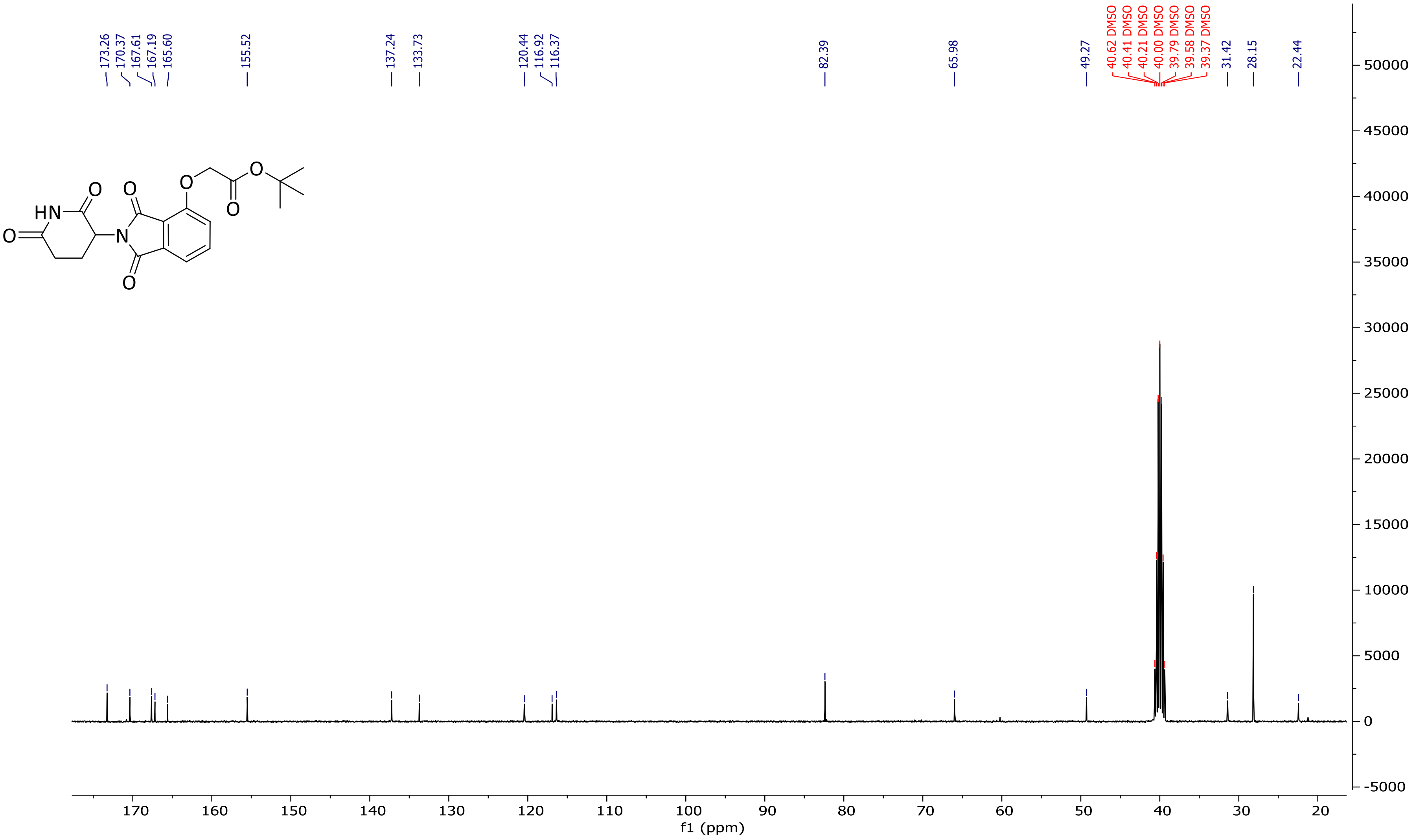

Figure S18. ^{13}C NMR (101 MHz) spectrum of *tert*-butyl 2-((2-(2,6-dioxopiperidin-3-yl)-1,3-dioxoisoindolin-4-yl)oxy)acetate (**9**) in DMSO-d_6

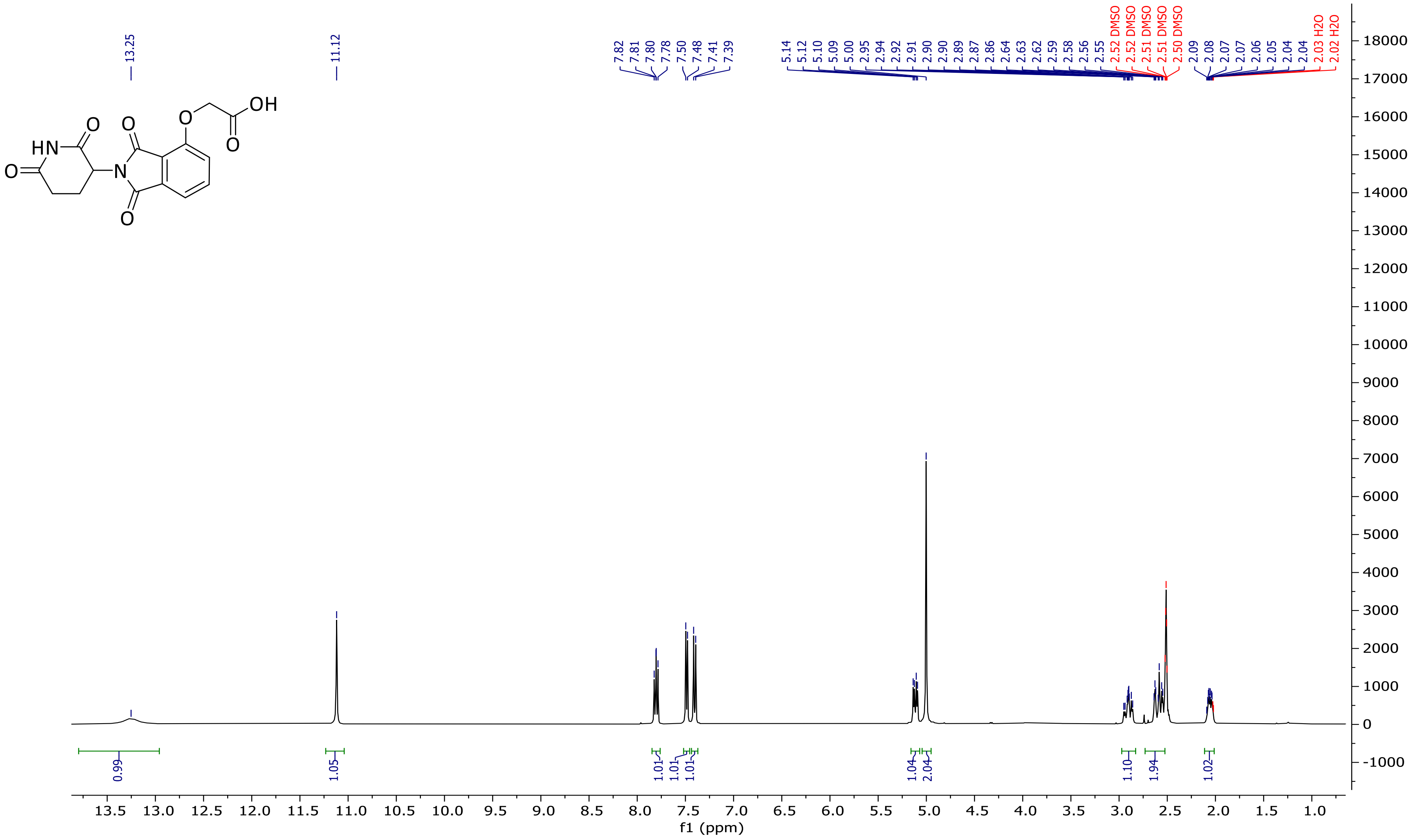

Figure S19. ^{1}H NMR (400 MHz) spectrum of 2-((2-(2,6-dioxopiperidin-3-yl)-1,3-dioxoisoindolin-4-yl)oxy)acetic acid (**10**) in DMSO-d_6

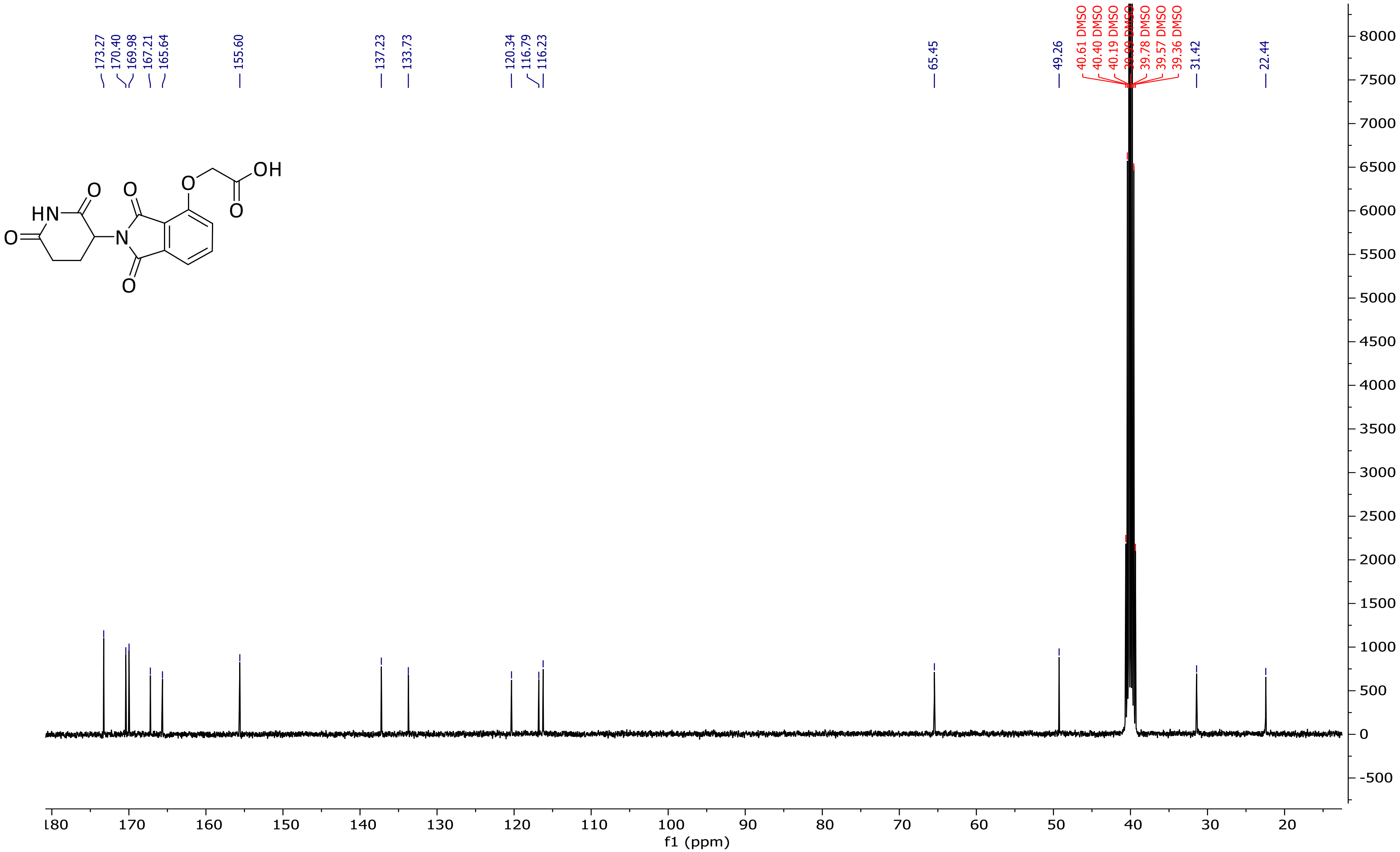

Figure S20. ^{13}C NMR (101 MHz) spectrum of 2-((2-(2,6-dioxopiperidin-3-yl)-1,3-dioxoisoindolin-4-yl)oxy)acetic acid (**10**) in DMSO-d_6

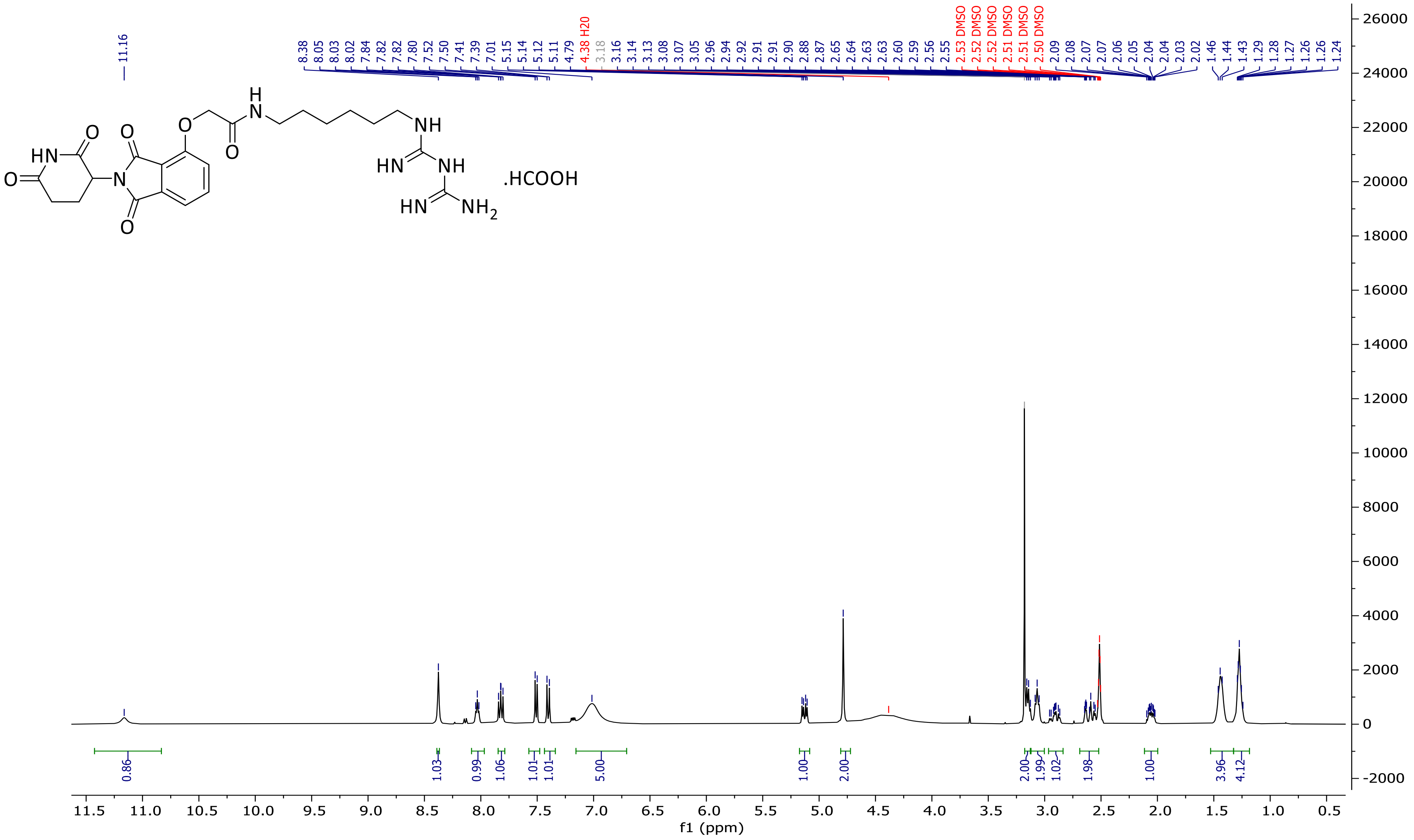

Figure S21. ^{1}H NMR (400 MHz) spectrum of *N*-(6-aminohexylbiguanide)-2-((2-(2,6-dioxopiperidin-3-yl)-1,3-dioxoisoindolin-4-yl)oxy)acetamide formate (**11**) in DMSO-d_6

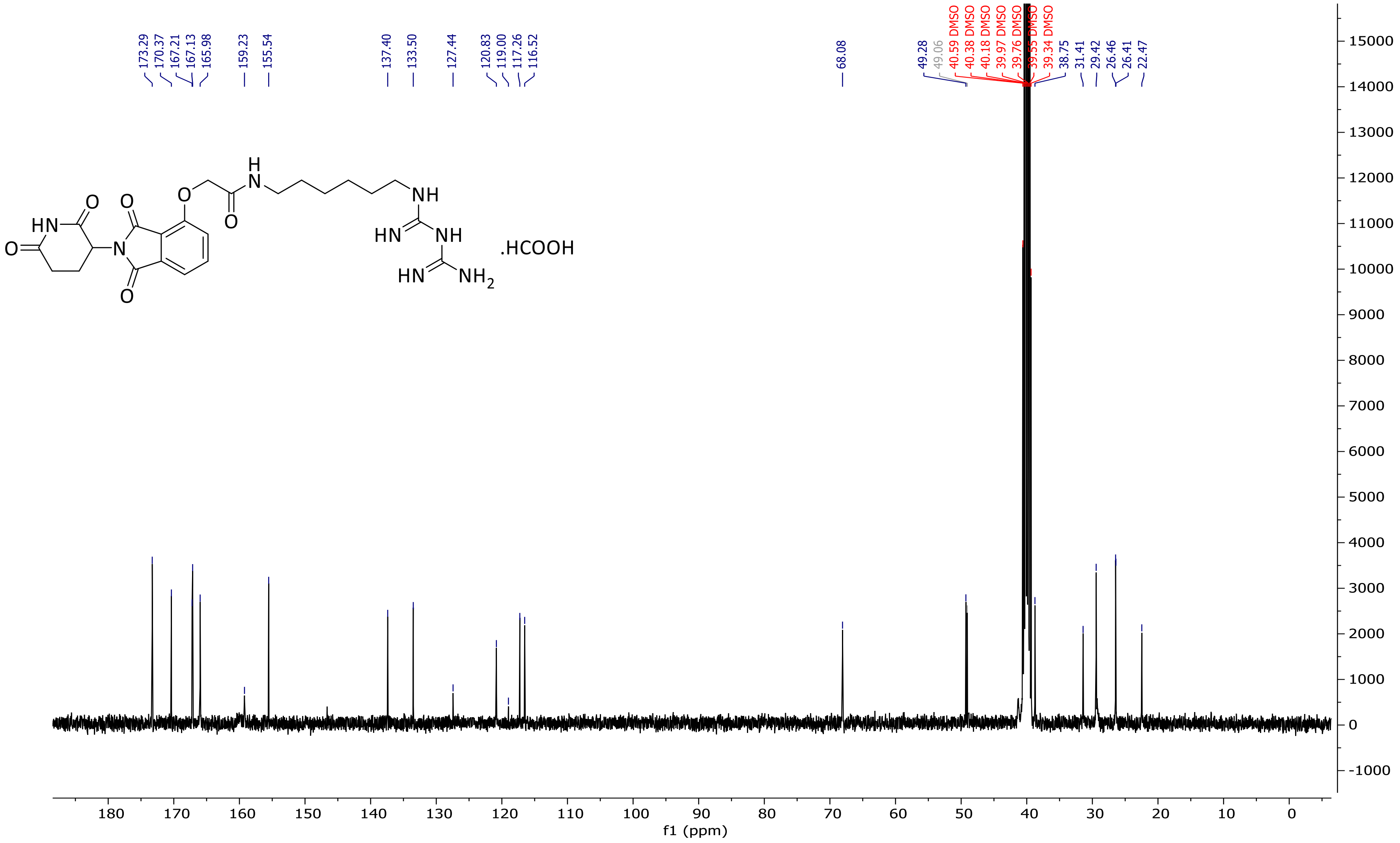

Figure S22. ^{13}C NMR (101 MHz) spectrum of *N*-(6-aminohexylbiguanide)-2-((2-(2,6-dioxopiperidin-3-yl)-1,3-dioxoisoindolin-4-yl)oxy)acetamide formate (**11**) in DMSO-d_6

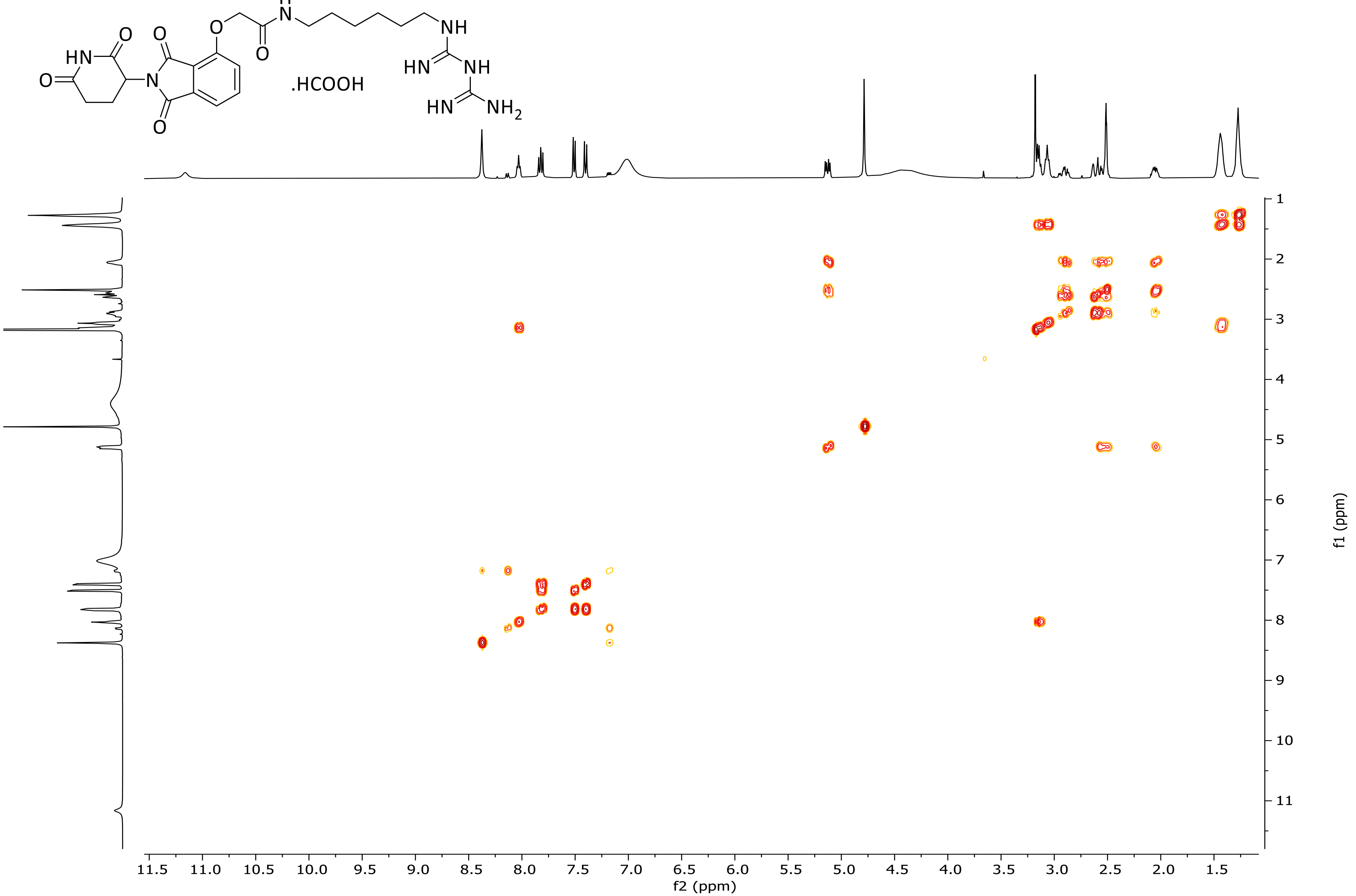

Figure S23. COSY NMR (400 MHz) spectrum of *N*-(6-aminohexylbiguanide)-2-((2-(2,6-dioxopiperidin-3-yl)-1,3-dioxoisoindolin-4-yl)oxy)acetamide formate (**11**) in DMSO-d_6

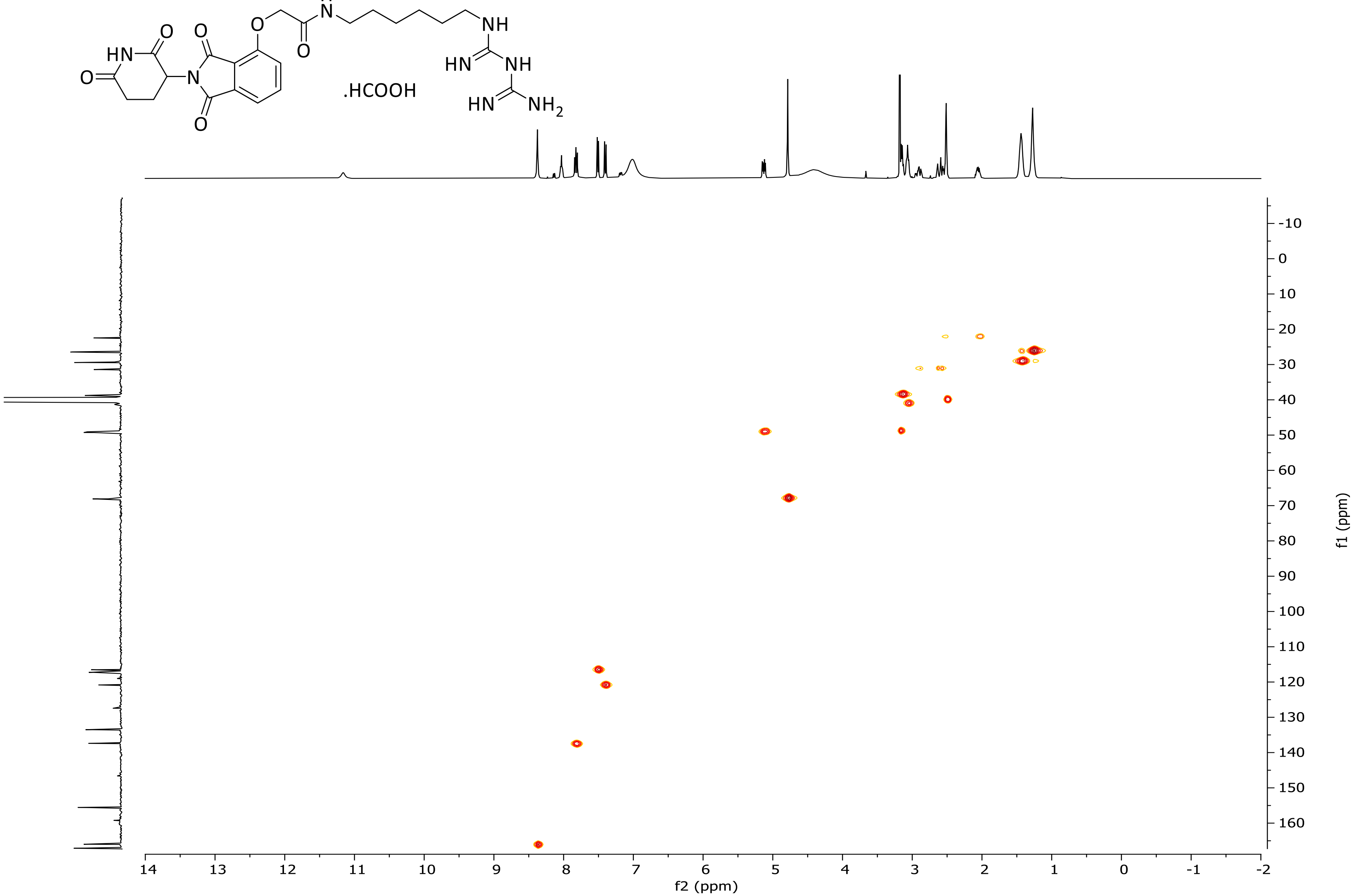

Figure S24. HSQC NMR spectrum of *N*-(6-aminohexylbiguanide)-2-((2-(2,6-dioxopiperidin-3-yl)-1,3-dioxoisoindolin-4-yl)oxy)acetamide formate (**11**) in DMSO-d_6

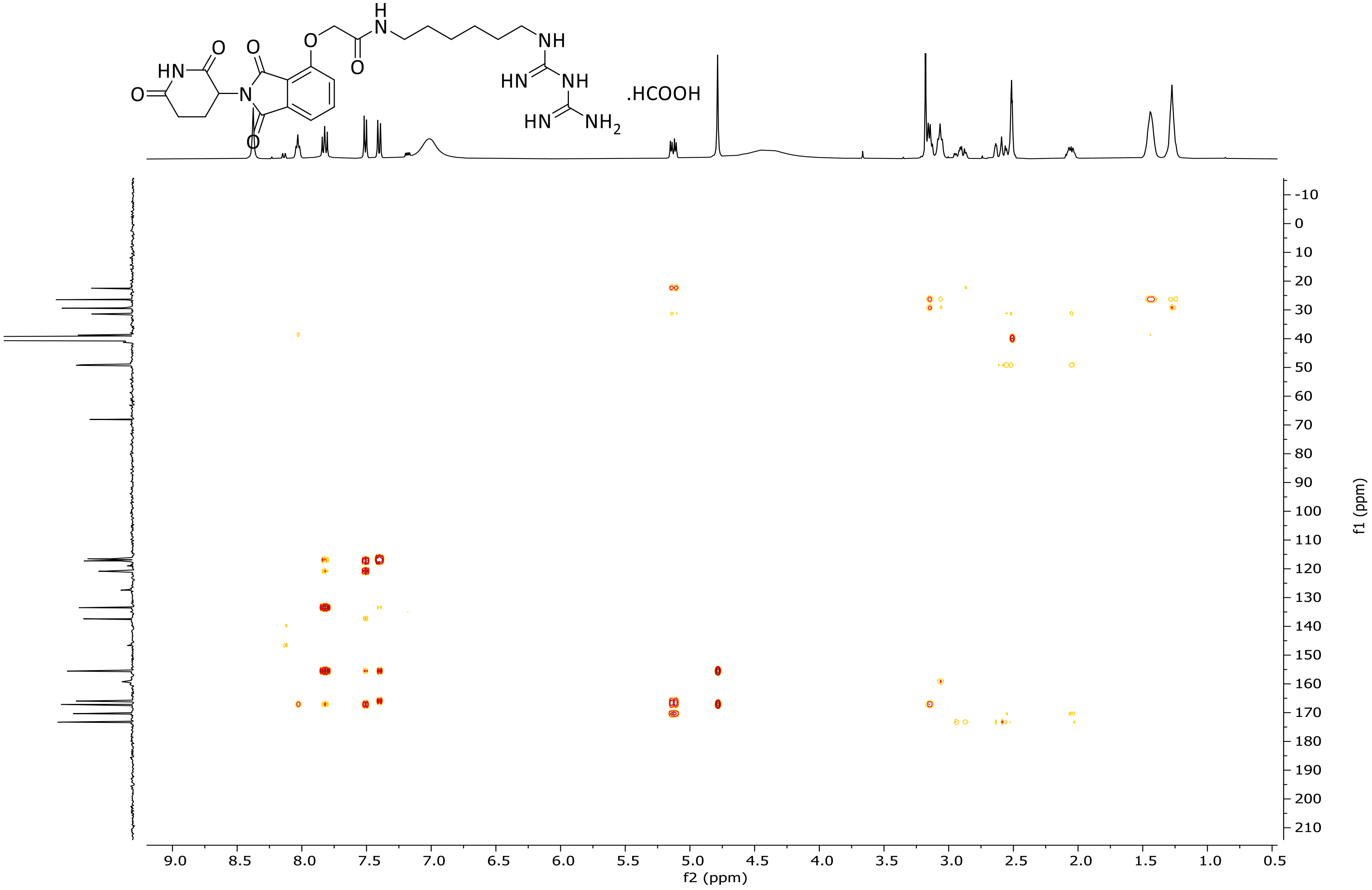

Figure S25. HMBC NMR spectrum of *N*-(6-aminohexylbiguanide)-2-((2-(2,6-dioxopiperidin-3-yl)-1,3-dioxoisoindolin-4-yl)oxy)acetamide formate (**11**) in DMSO-d_6

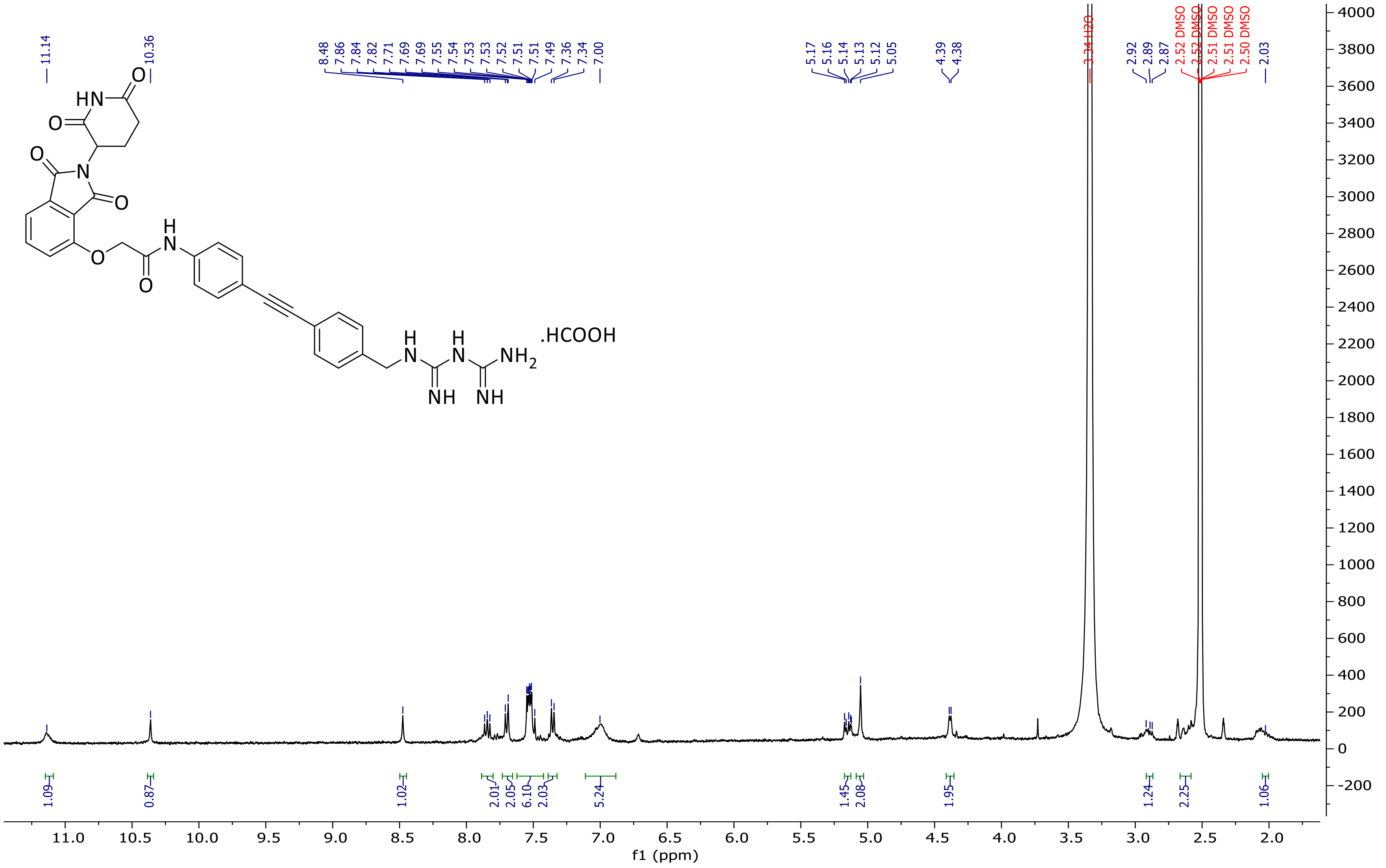

Figure S26. ^{1}H NMR (400 MHz) spectrum of *N*-(4-((4-((3-biguanidemethyl)phenyl) ethynyl)phenyl)-2-((2-(2,6-dioxopiperidin-3-yl)-1,3-dioxoisoindolin-4-yl)oxy)acetamide formate (**12**) in DMSO-d_6

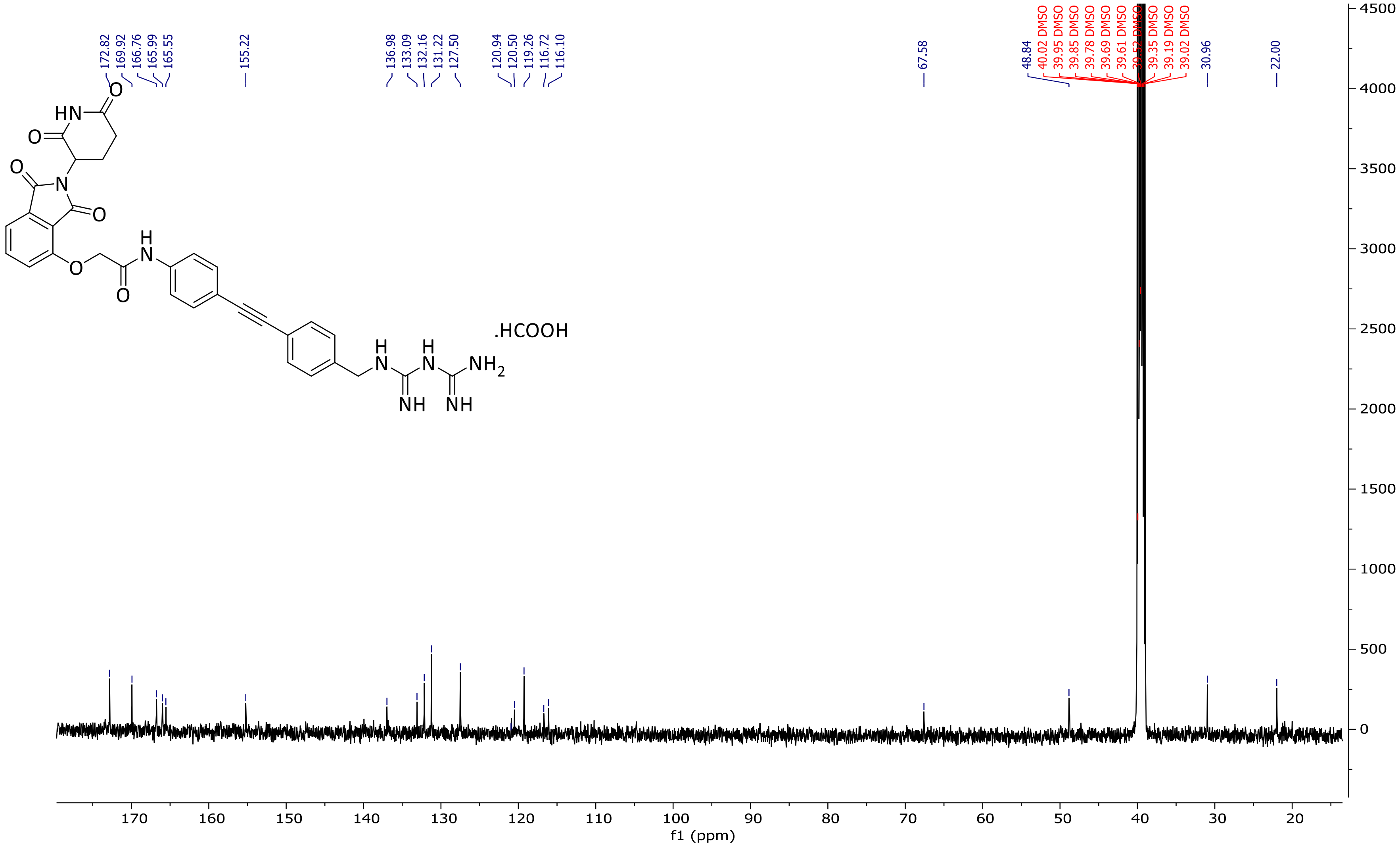

Figure S27. ^{13}C NMR (101 MHz) spectrum of *N*-(4-((4-((3-biguanidemethyl)phenyl) ethynyl)phenyl)-2-((2-(2,6-dioxopiperidin-3-yl)-1,3-dioxoisoindolin-4-yl)oxy)acetamide formate (**12**) in DMSO-d_6

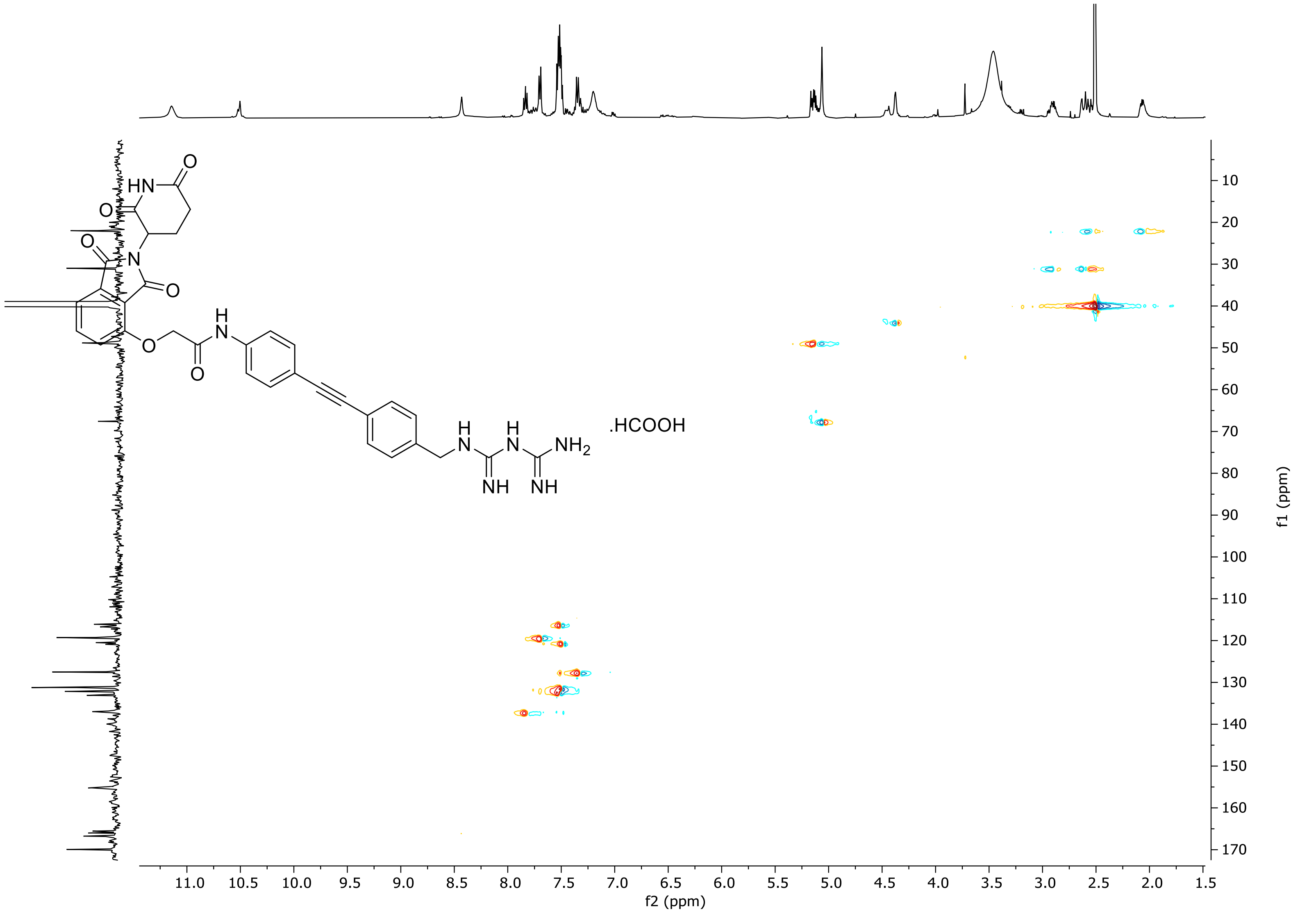

Figure S28. HSQC edited spectrum of *N*-(4-((4-((3-biguanidemethyl)phenyl) ethynyl) phenyl)-2-((2-(2,6-dioxopiperidin-3-yl)-1,3-dioxoisoindolin-4-yl)oxy)acetamide formate (**12**) in DMSO-d_6

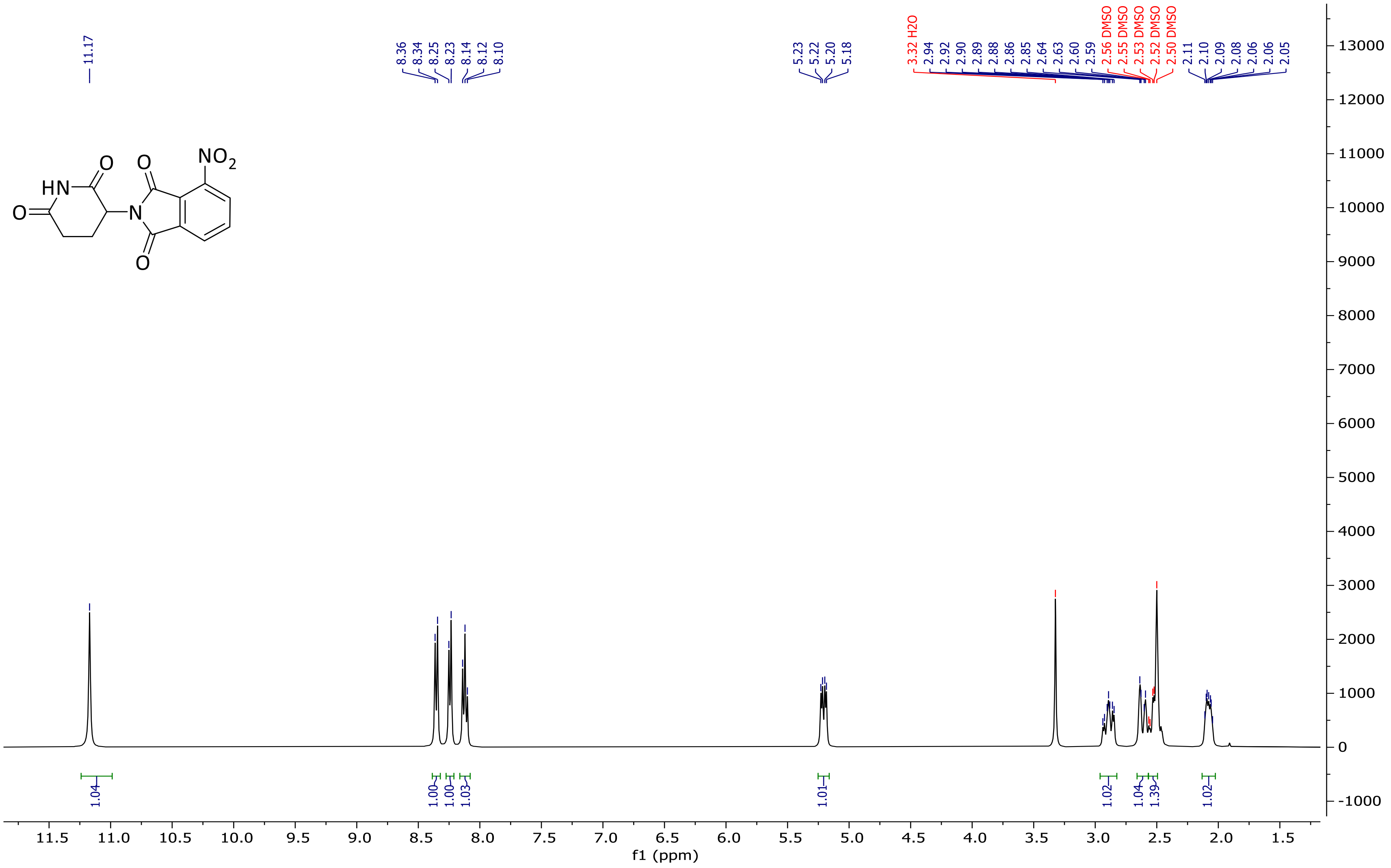

Figure S29. ^1H NMR (400 MHz) spectrum of 2-(2,6-dioxopiperidin-3-yl)-4-nitroisoindoline-1,3-dione (**13**) in DMSO-d_6

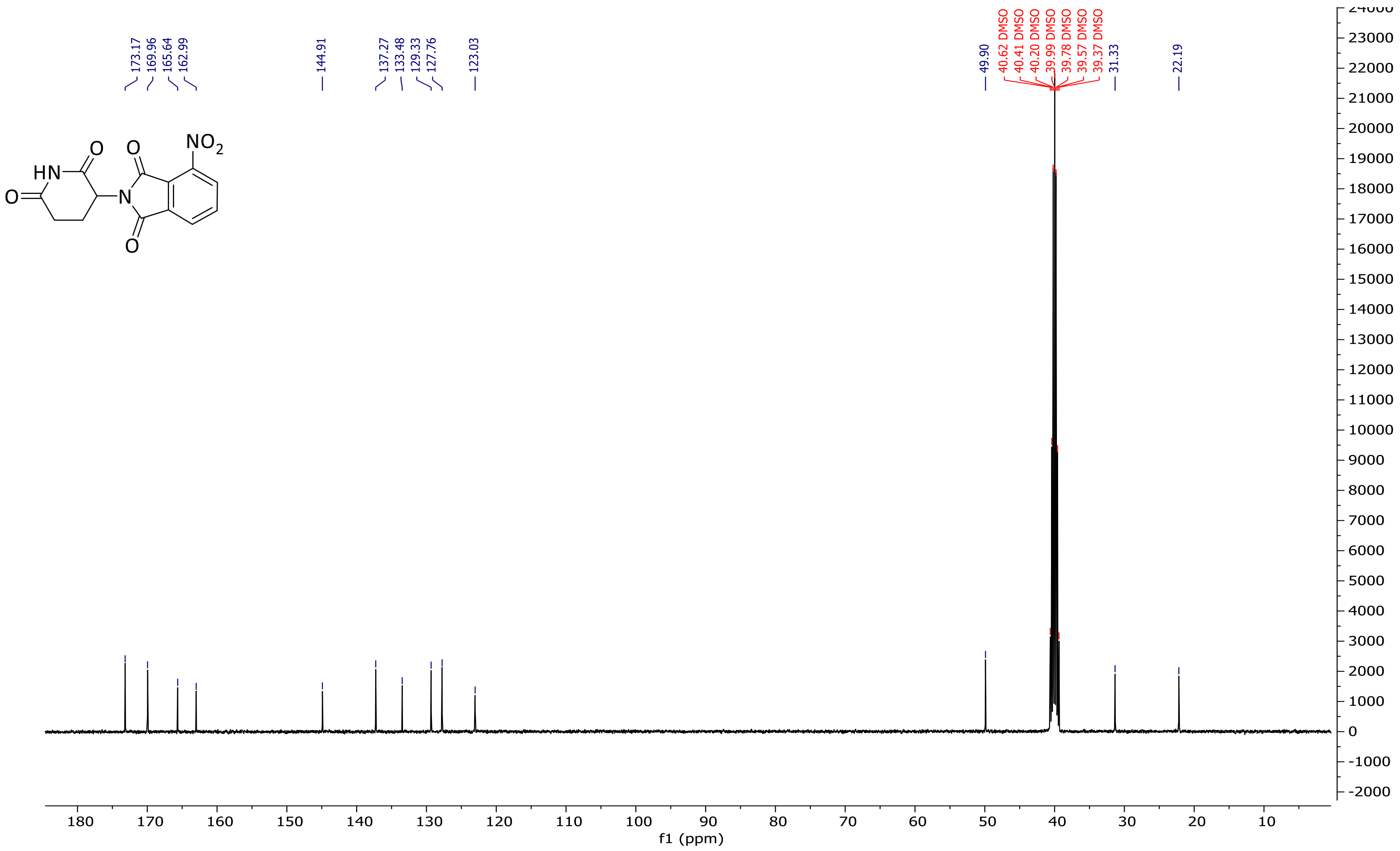

Figure S30. ^{13}C NMR (101 MHz) spectrum of 2-(2,6-dioxopiperidin-3-yl)-4-nitroisoindoline-1,3-dione (**13**) in DMSO-d_6

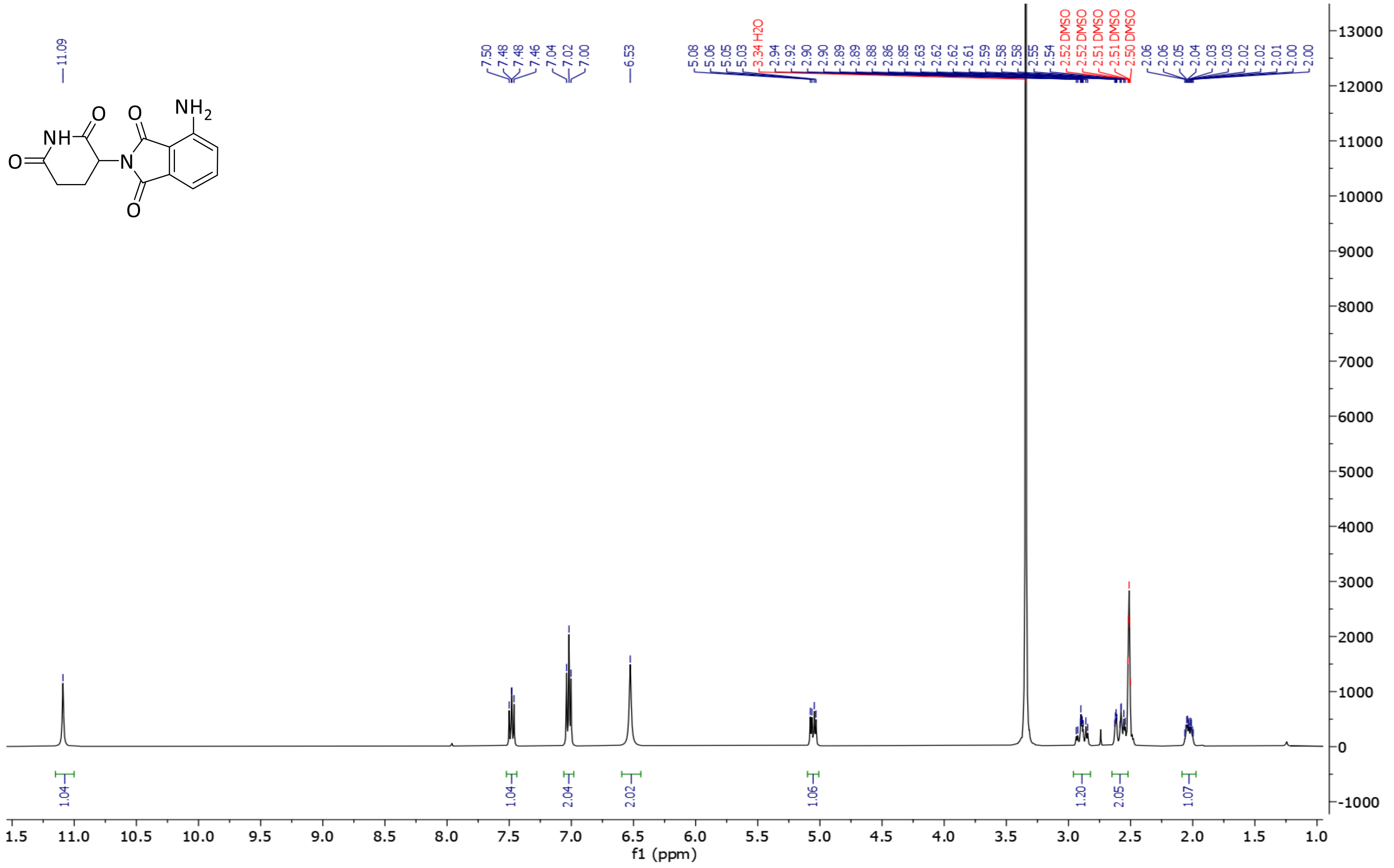

Figure S31. ^{1}H NMR (400 MHz) spectrum of 4-amino-2-(2,6-dioxopiperidin-3-yl)isoindoline-1,3-dione (**14**) in DMSO-d_6

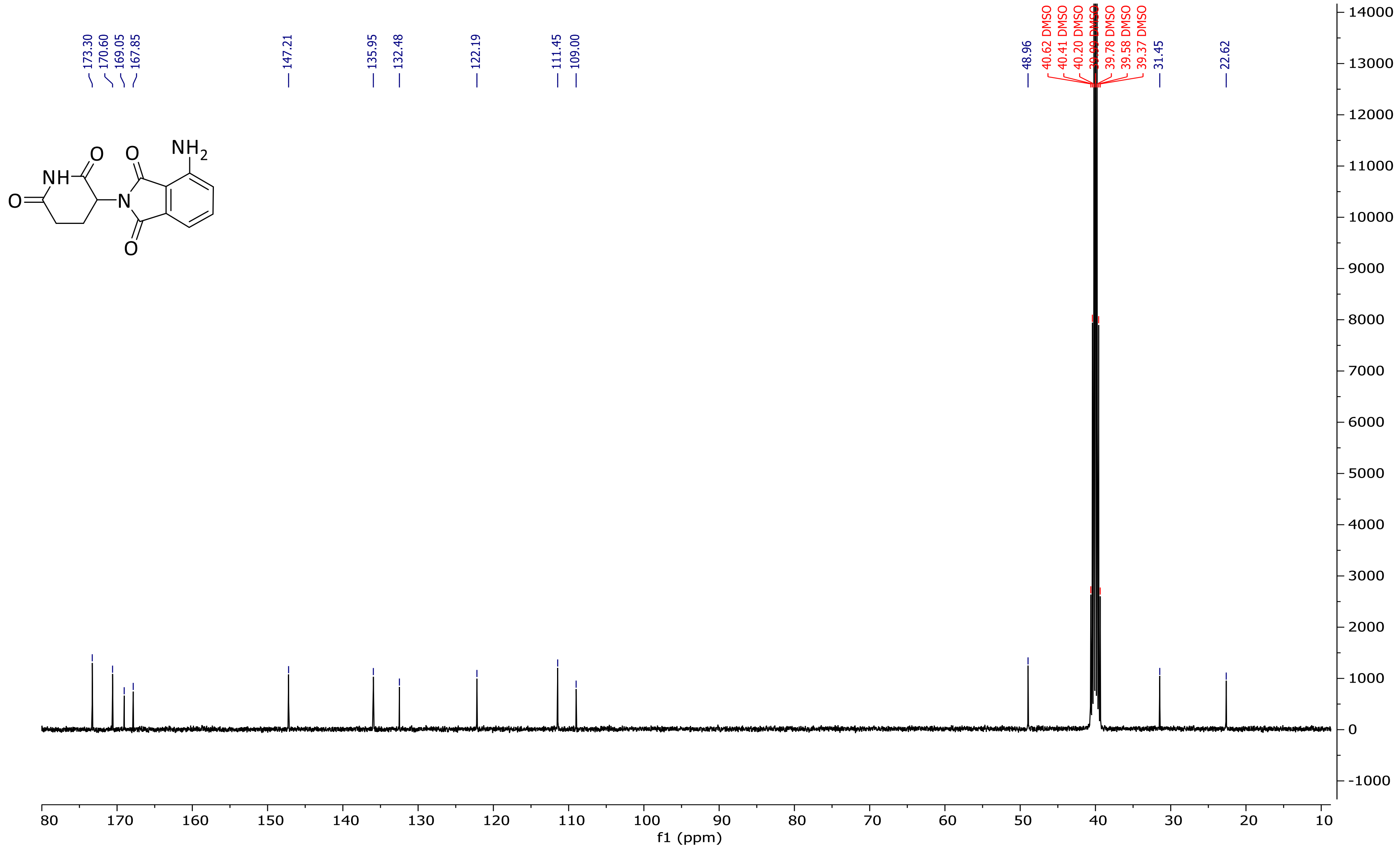

Figure S32. ^{13}C NMR (101 MHz) spectrum of 4-amino-2-(2,6-dioxopiperidin-3-yl) isoindoline-1,3-dione (**14**) in DMSO-d_6

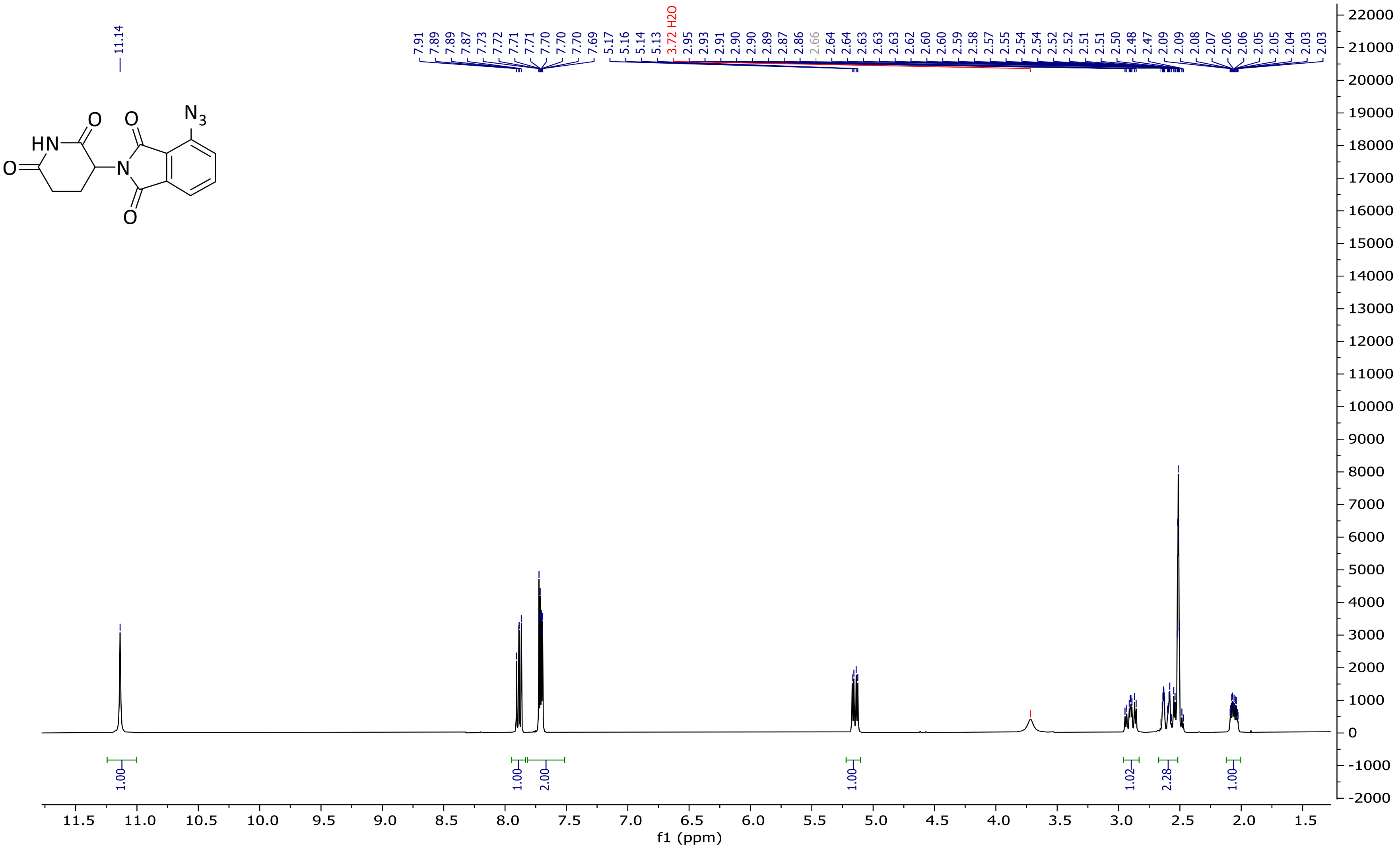

Figure S33. ^{1}H NMR (400 MHz) spectrum of 4-azido-2-(2,6-dioxopiperidin-3-yl)isoindoline-1,3-dione (**15**) in DMSO-d_6

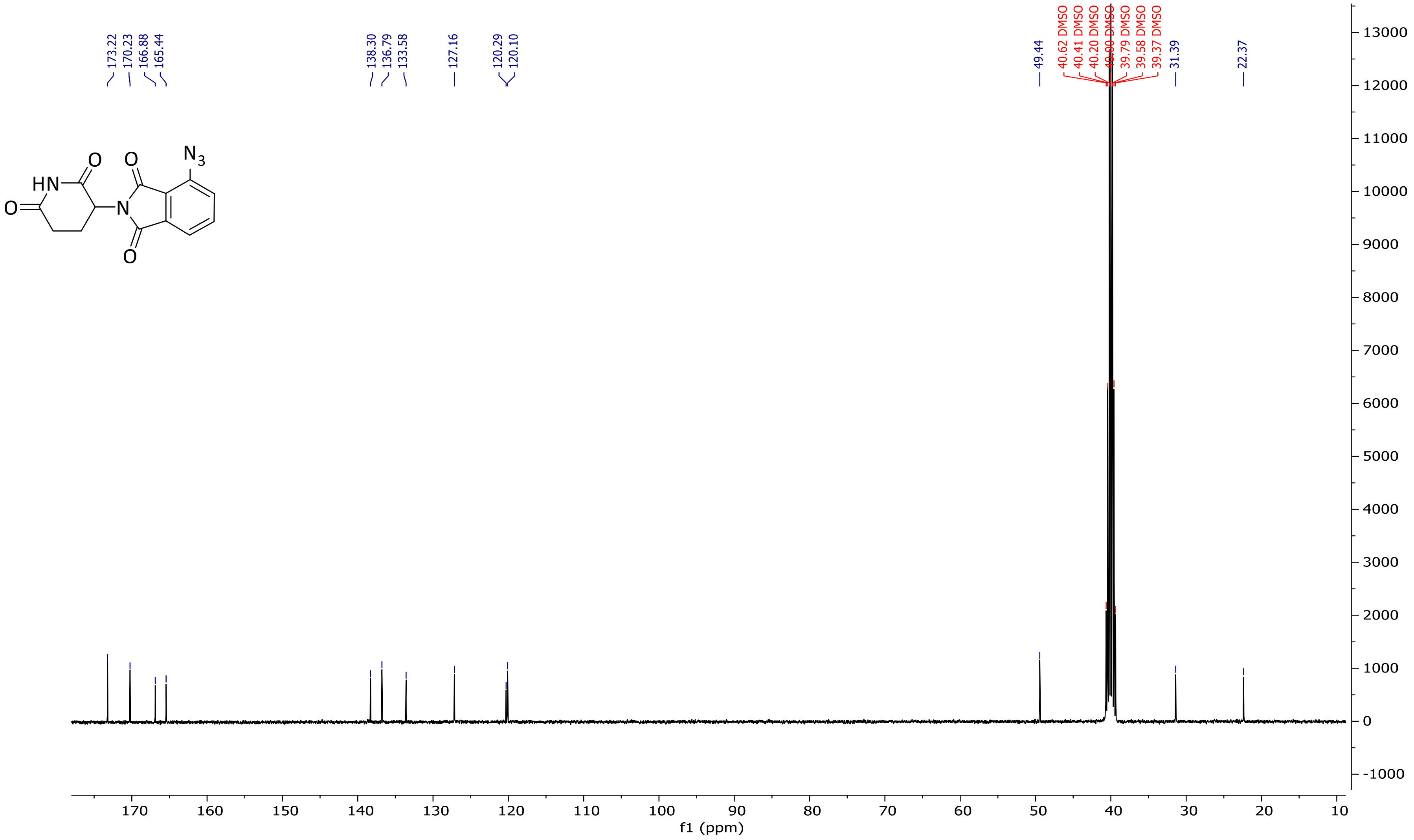

Figure S34. ^{13}C NMR (101 MHz) spectrum of 4-azido-2-(2,6-dioxopiperidin-3-yl) isoindoline-1,3-dione (**15**) in DMSO-d_6

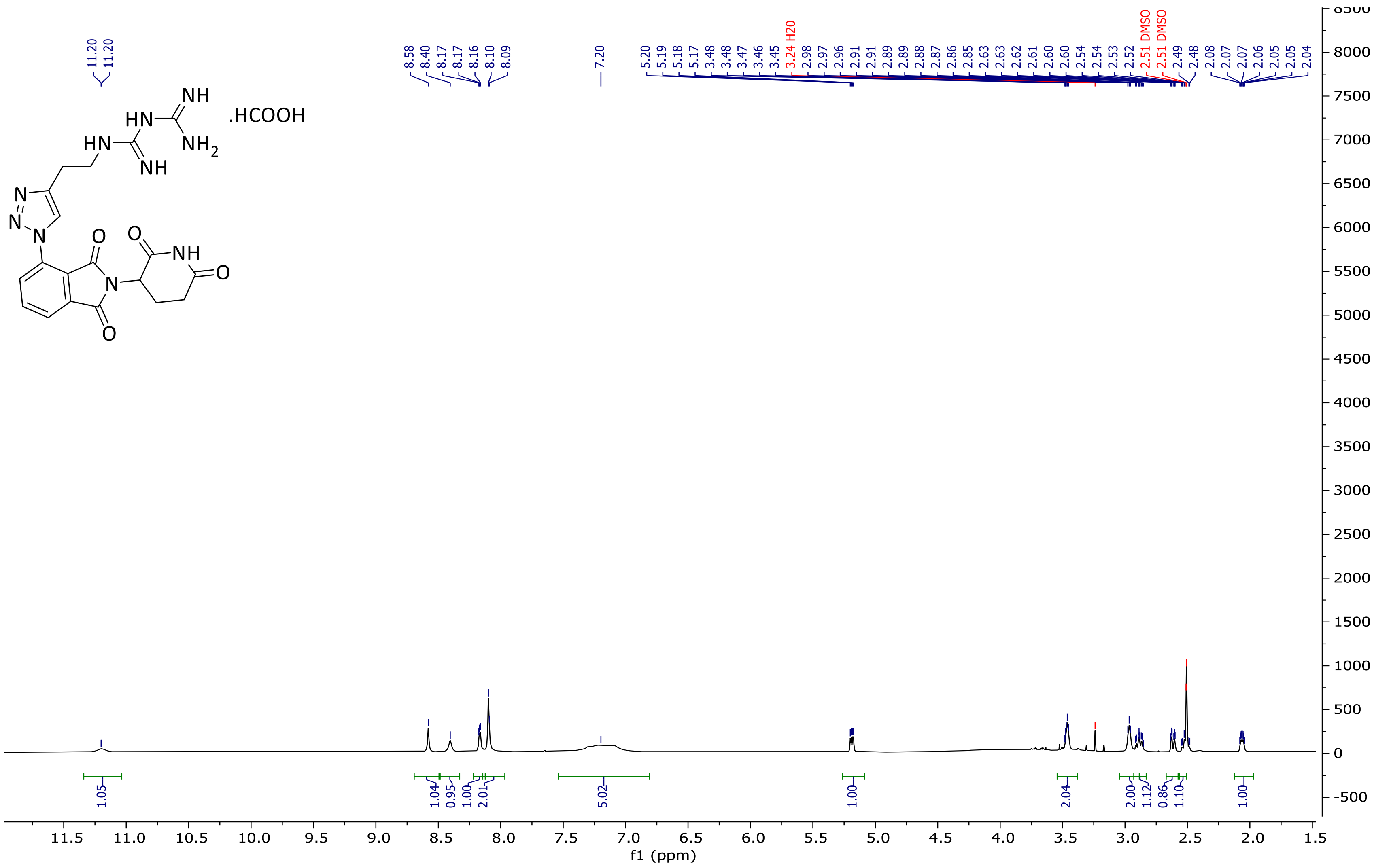

Figure S35. ^{1}H NMR (400 MHz) spectrum of 4-(4-(2-biguanide-ethyl)-1H-1,2,3-triazol-1-yl) -2-(2,6-dioxopiperidin-3-yl) isoindoline-1,3-dione formate (**16**) in DMSO-d_6

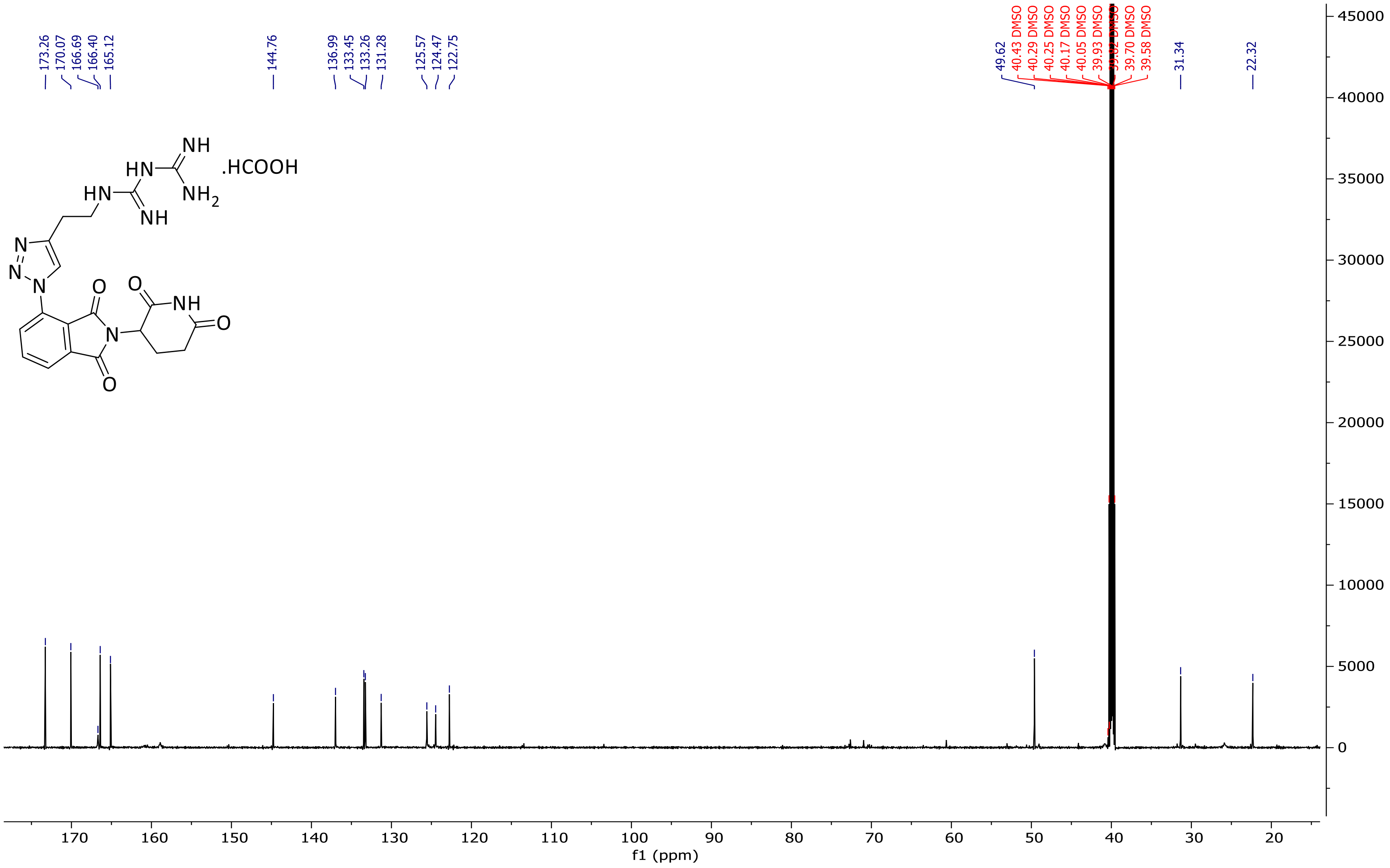

Figure S36. ^{13}C NMR (176 MHz) spectrum of 4-(4-(2-biguanide-ethyl)-1H -1,2,3-triazol-1-yl)-2-(2,6-dioxopiperidin-3-yl)isoindoline-1,3-dione formate (**16**) in DMSO-d_6

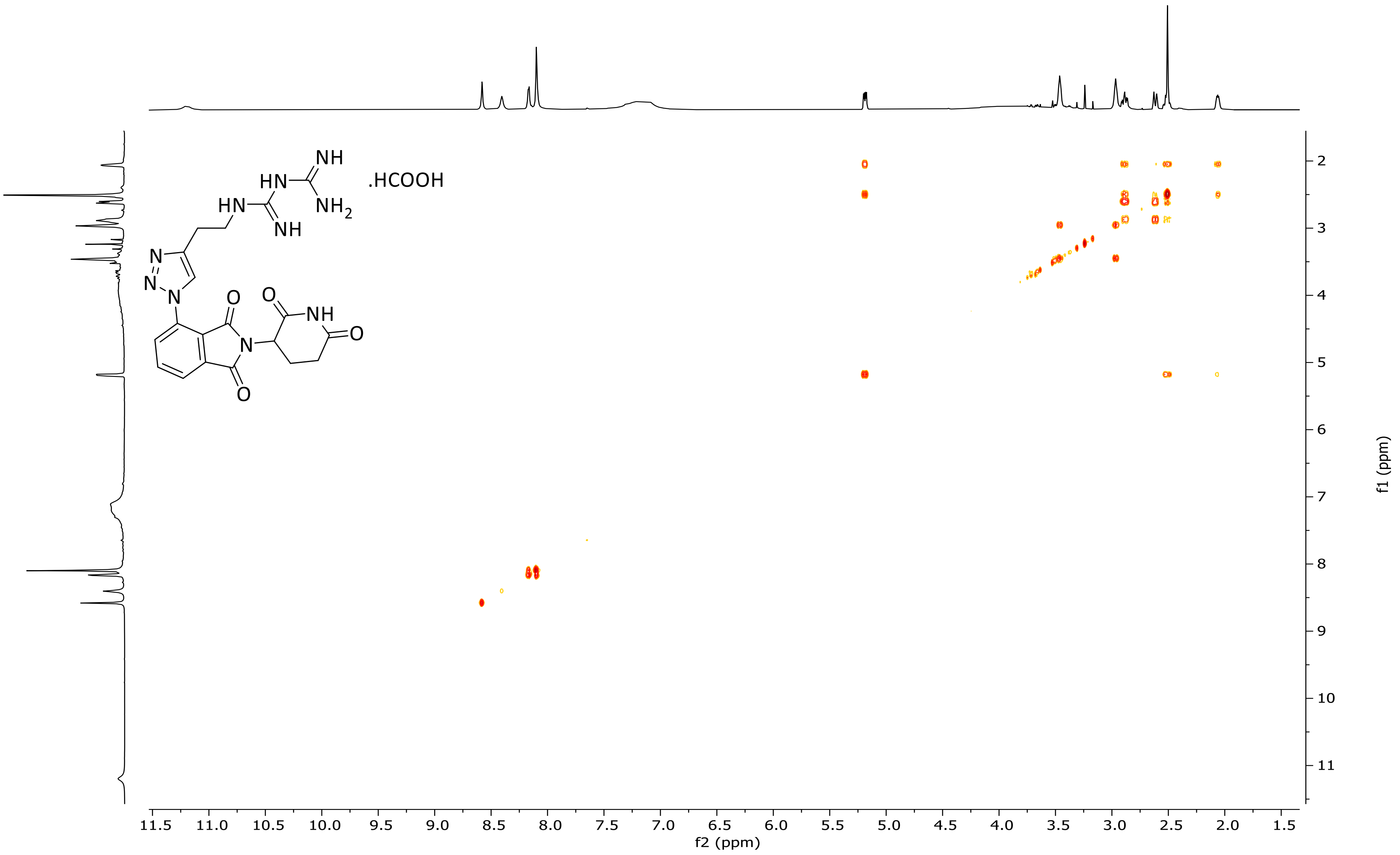

Figure S37. COSY NMR (400 MHz) spectrum of 4-(4-(2-biguanide-ethyl)-1H- 1,2,3-triazol-1-yl)-2-(2,6-dioxopiperidin-3-yl)isoindoline-1,3-dione formate (**16**) in DMSO-d_6

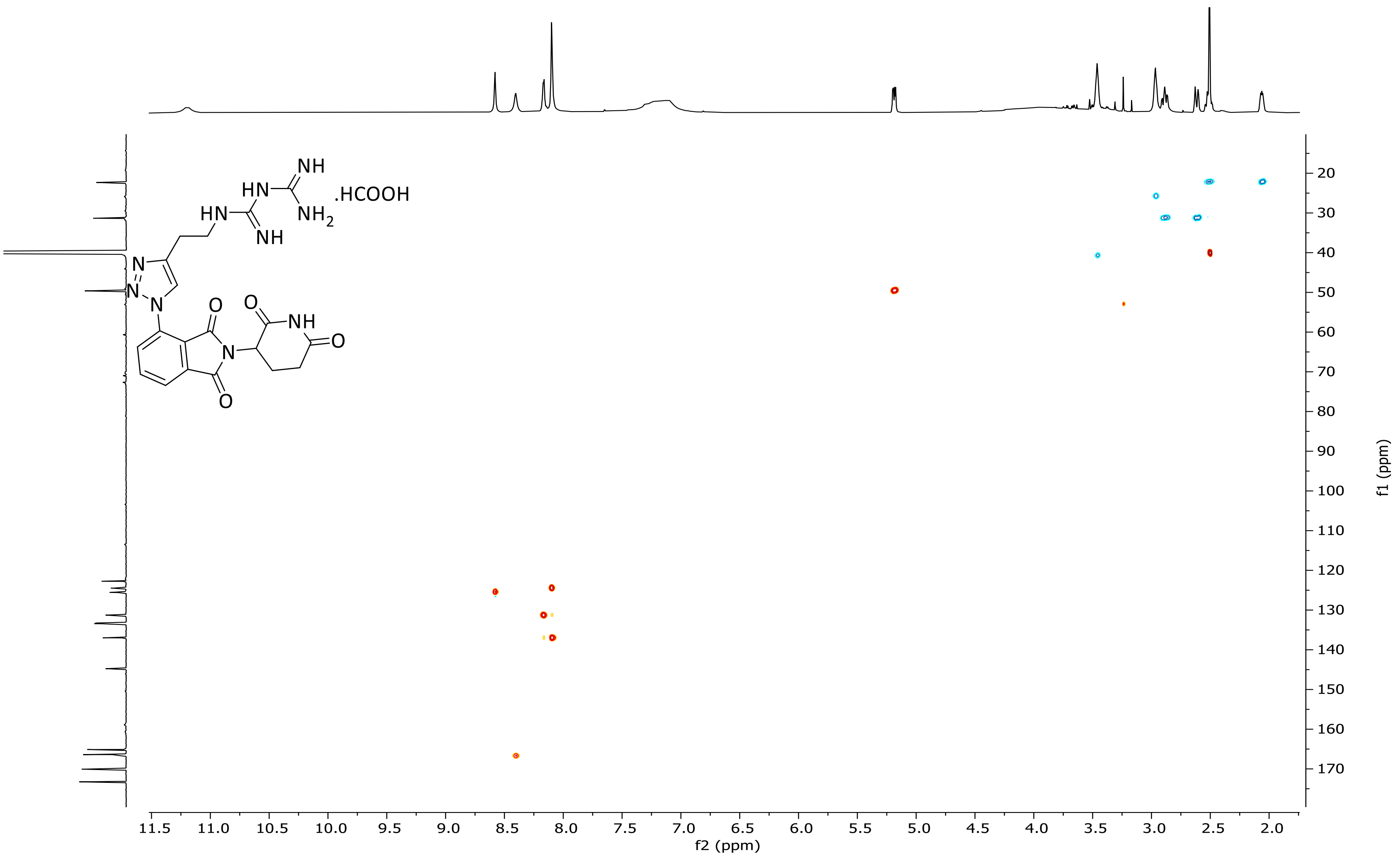

Figure S38. HSQC editied NMR spectrum of 4-(4-(2-biguanide-ethyl)-1H -1,2,3-triazol-1-yl)-2-(2,6-dioxopiperidin-3-yl)isoindoline-1,3-dione formate (**16**) in DMSO-d_6

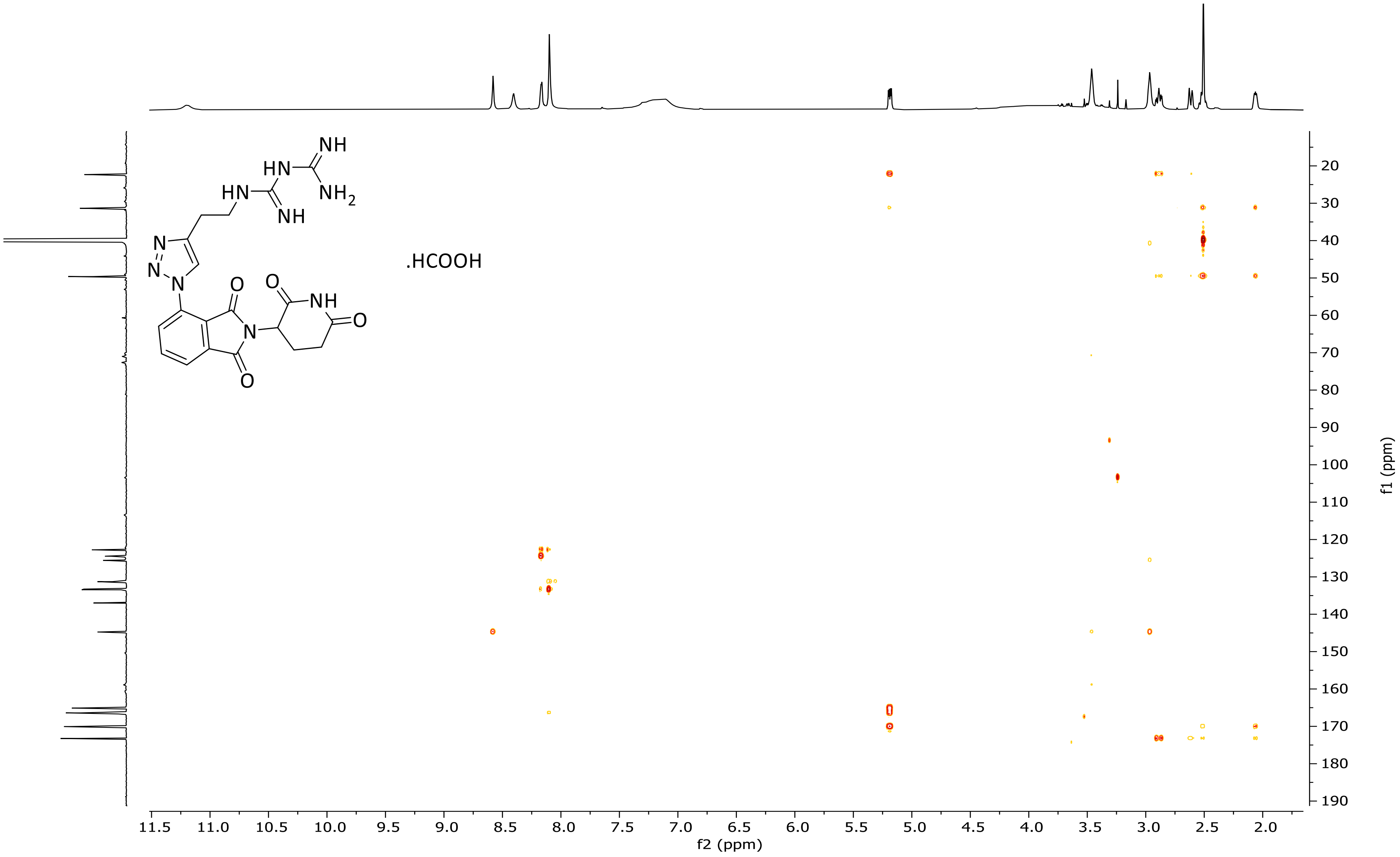

Figure S39. HMBC NMR spectrum of 4-(4-(2-biguanide-ethyl)-1H-1,2,3-triazol-1-yl)-2-(2,6-dioxopiperidin-3-yl)isoindoline-1,3-dione formate (**16**) in DMSO-*d*$_6$

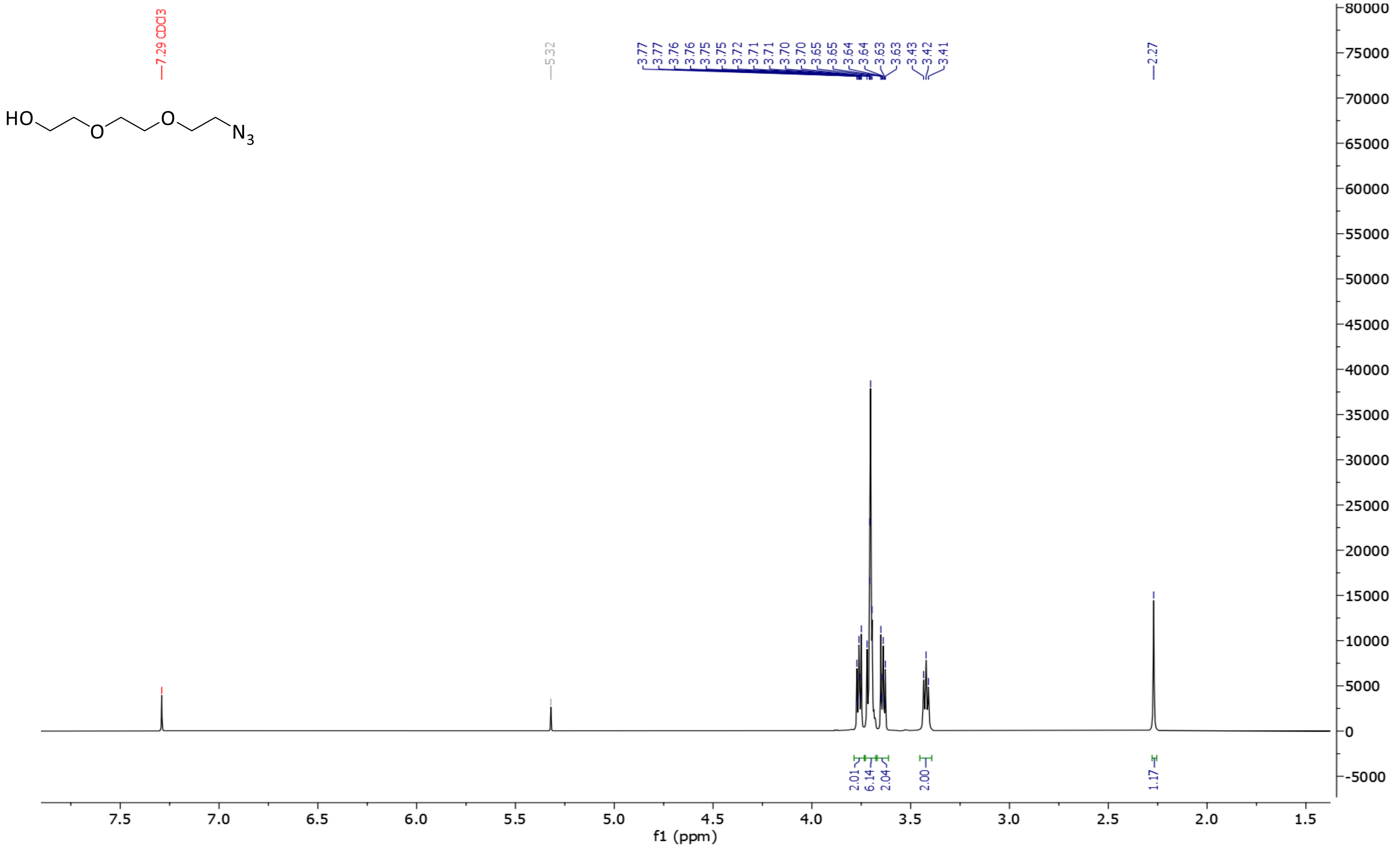

Figure S40. ^{1}H NMR (400 MHz) spectrum of 2-(2-(2-azidoethoxy)ethoxy)ethan-1-ol (**17**) in chloroform-*d*

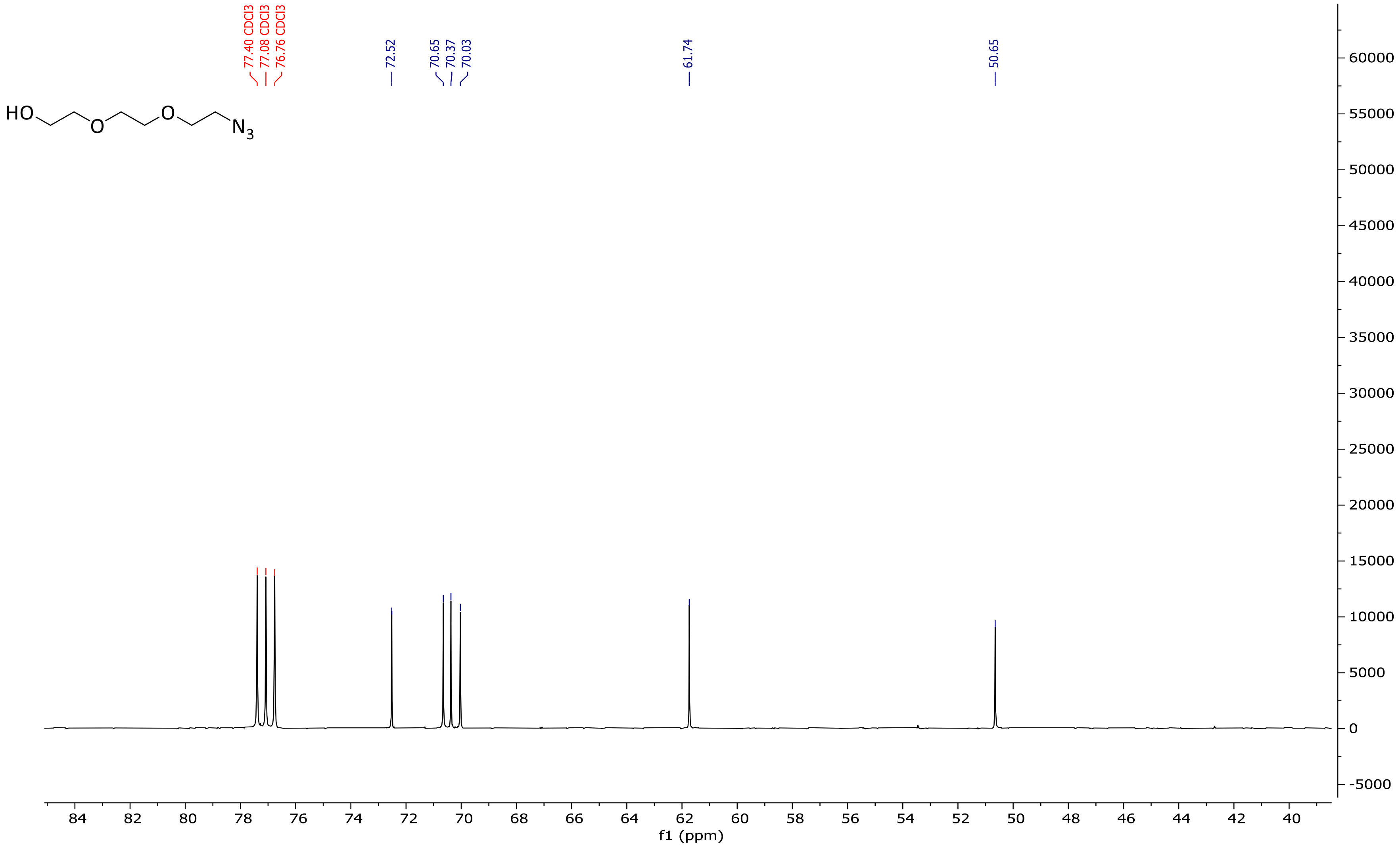

Figure S41. ^{13}C NMR (101 MHz) spectrum of 2-(2-(2-azidoethoxy)ethoxy)ethan-1-ol (**17**) in chloroform-*d*

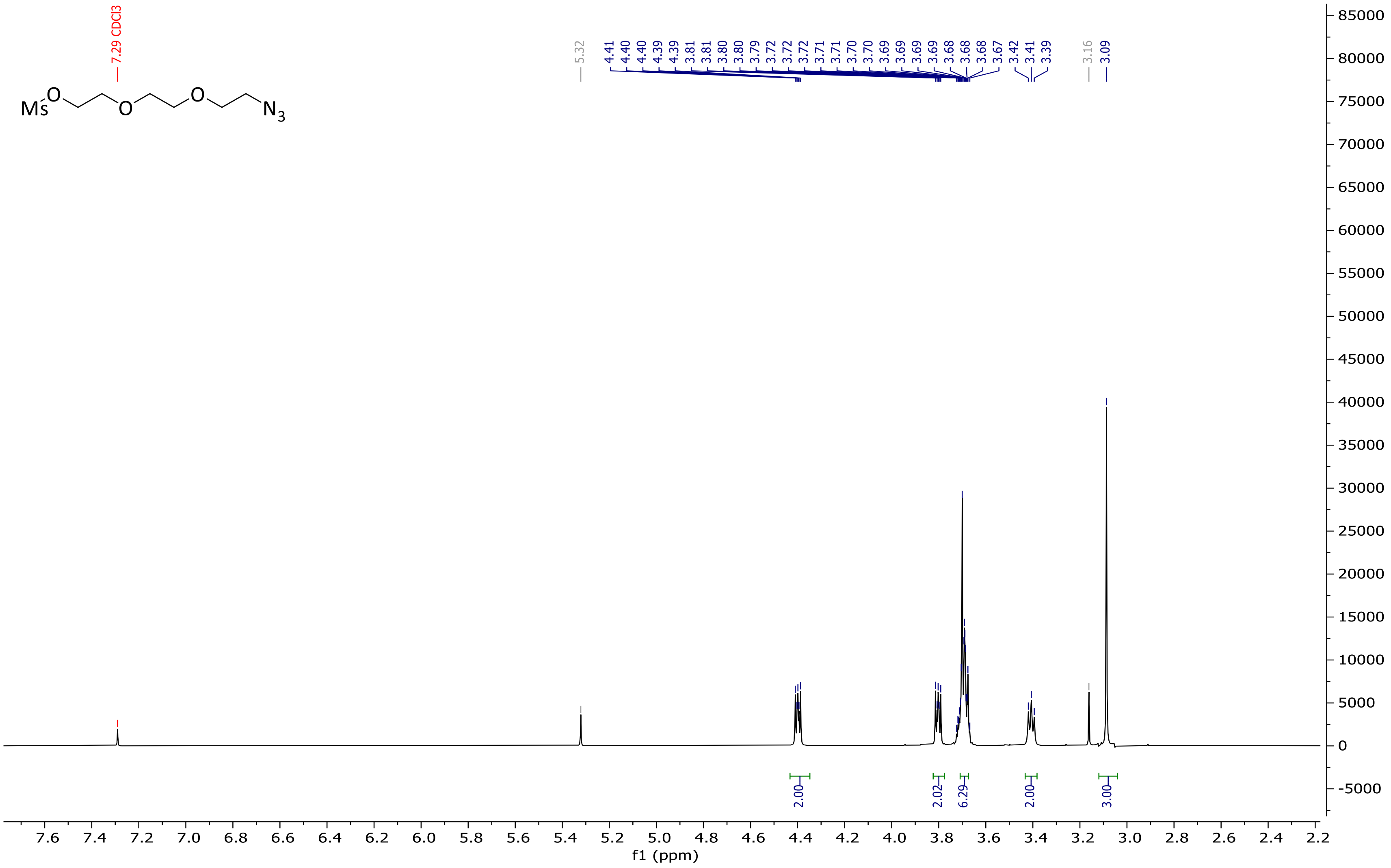

Figure S42. ^{1}H NMR (400 MHz) spectrum of 2-(2-(2-azidoethoxy)ethoxy)ethylmethane sulfonate (**18**) in chloroform-*d*

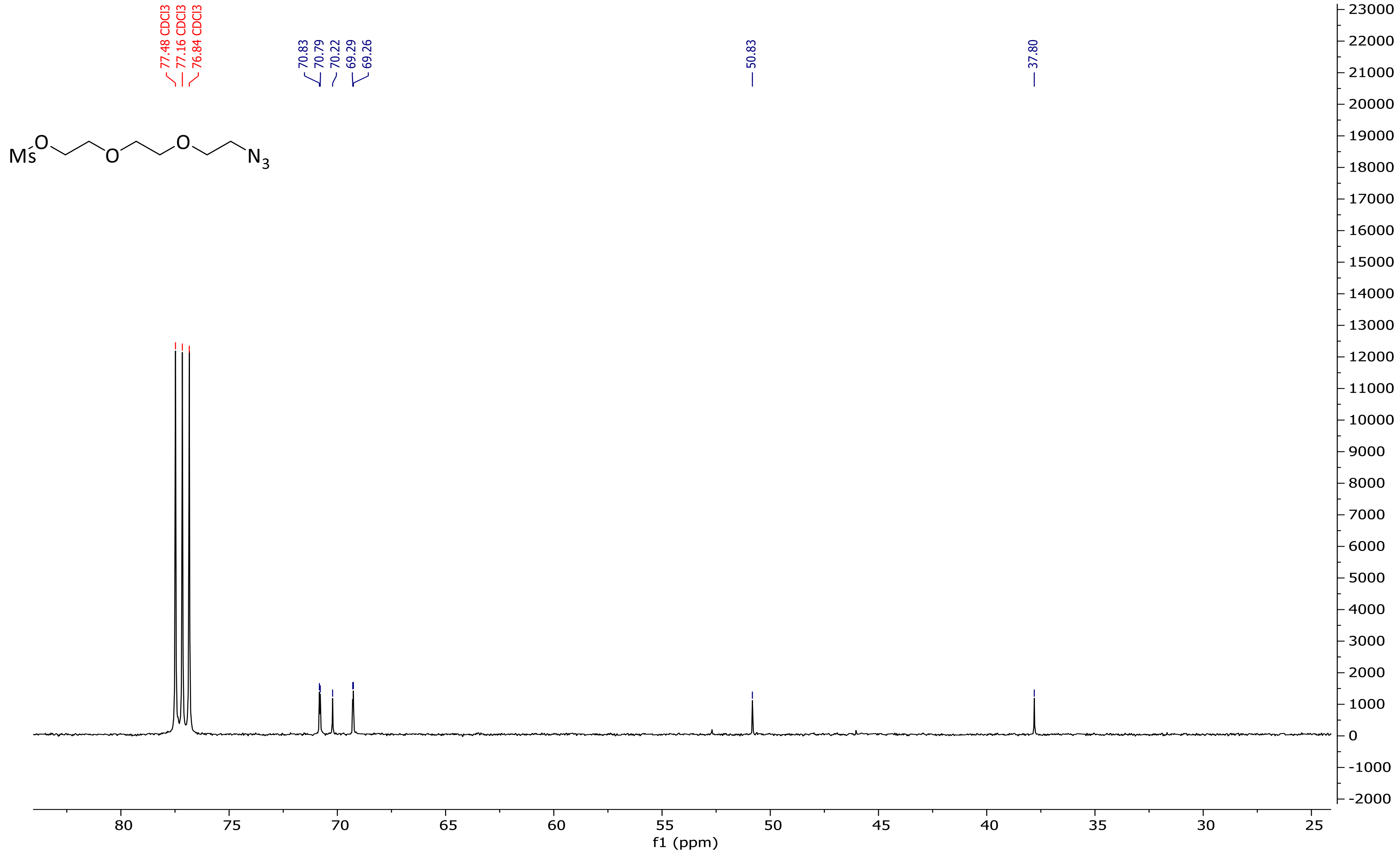

Figure S43. ^{13}C NMR (101 MHz) spectrum 2-(2-(2-azidoethoxy)ethoxy)ethylmethane sulfonate (**18**) in chloroform-*d*

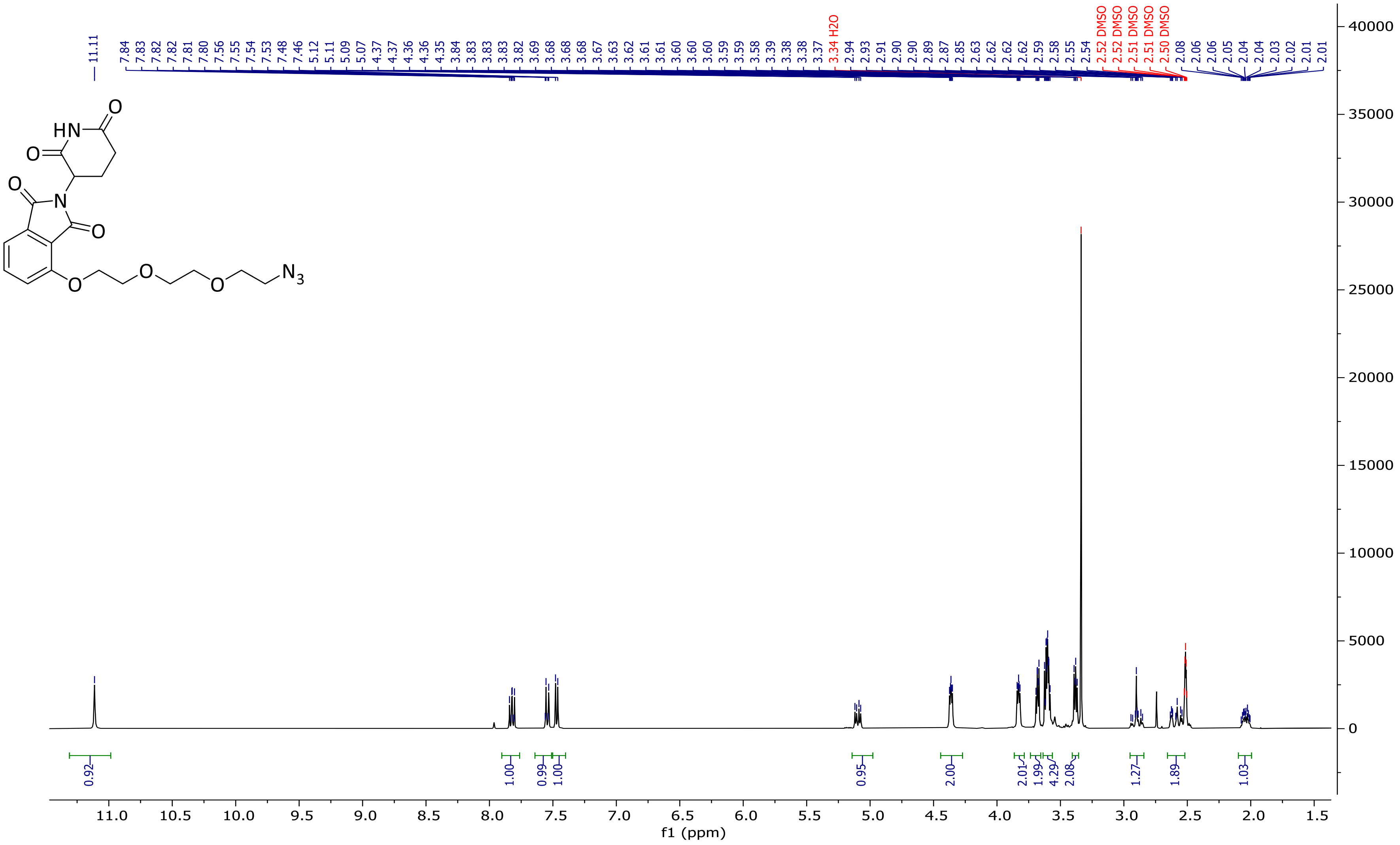

Figure S44. ^{1}H NMR (400 MHz) spectrum of 4-(2-(2-(2-azidoethoxy)ethoxy)ethoxy)-2-(2,6-dioxopiperidin-3-yl)isoindoline-1,3-dione (**19**) in DMSO-d_6

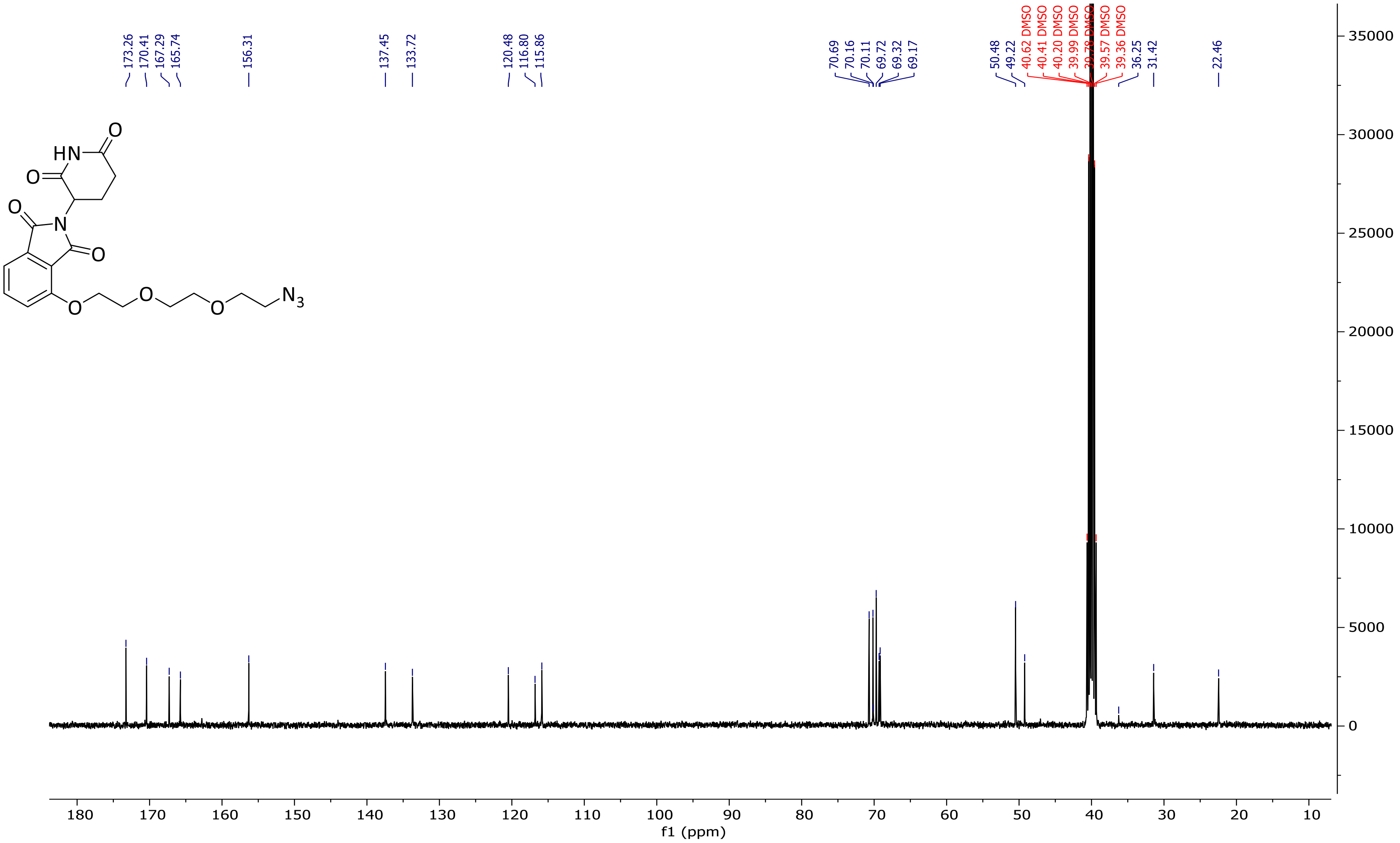

Figure S45. ^{13}C NMR (101 MHz) spectrum of 4-(2-(2-(2-azidoethoxy)ethoxy)ethoxy)-2-(2,6-dioxopiperidin-3-yl)isoindoline-1,3-dione (**19**) in DMSO-*d_6*

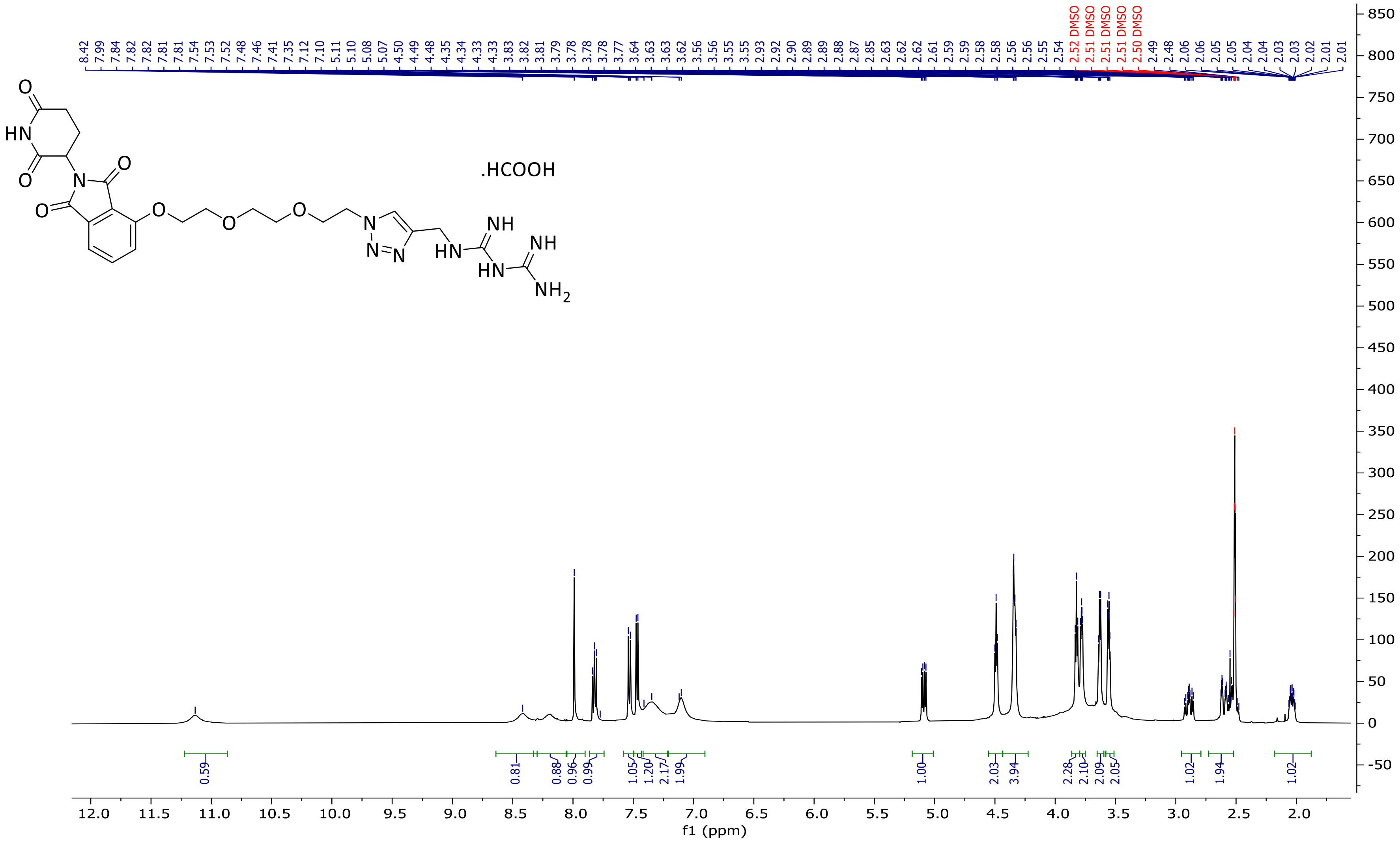

Figure S46. ^{1}H NMR (500 MHz) spectrum of 4-(2-(2-(2-(4-(biguanidemethyl)-1H -1,2,3-triazol-1-yl)ethoxy)ethoxy)ethoxy)-2-(2,6-dioxopiperidin-3-yl)isoindoline-1,3-dione formate (**20**) in DMSO-d_6

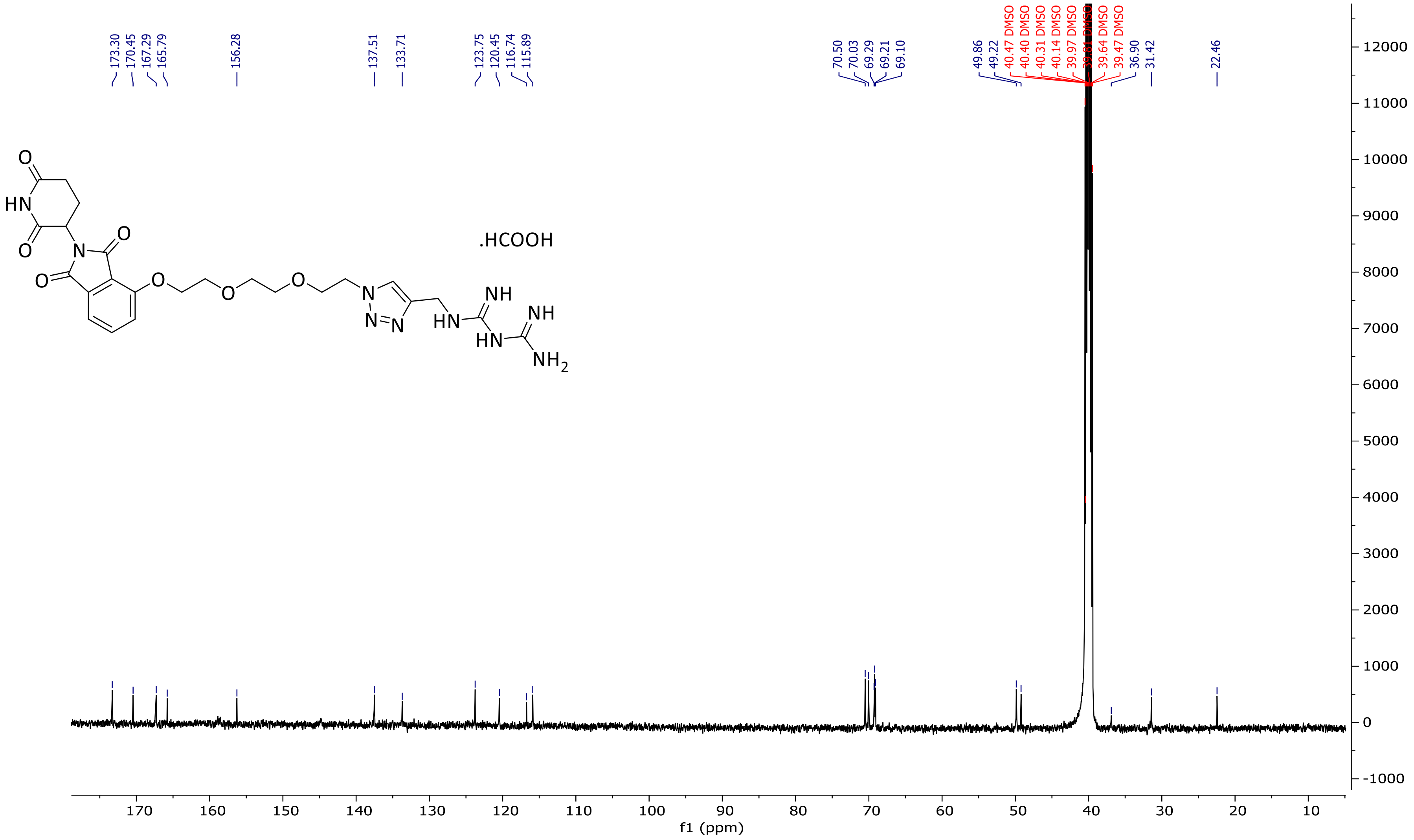

Figure S47. ^{13}C NMR (126 MHz) spectrum of 4-(2-(2-(2-(4-(biguanidemethyl)-1H -1,2,3-triazol-1-yl)ethoxy)ethoxy)ethoxy)-2-(2,6-dioxopiperidin-3-yl)isoindoline-1,3-dione formate (**20**) in DMSO-d_6

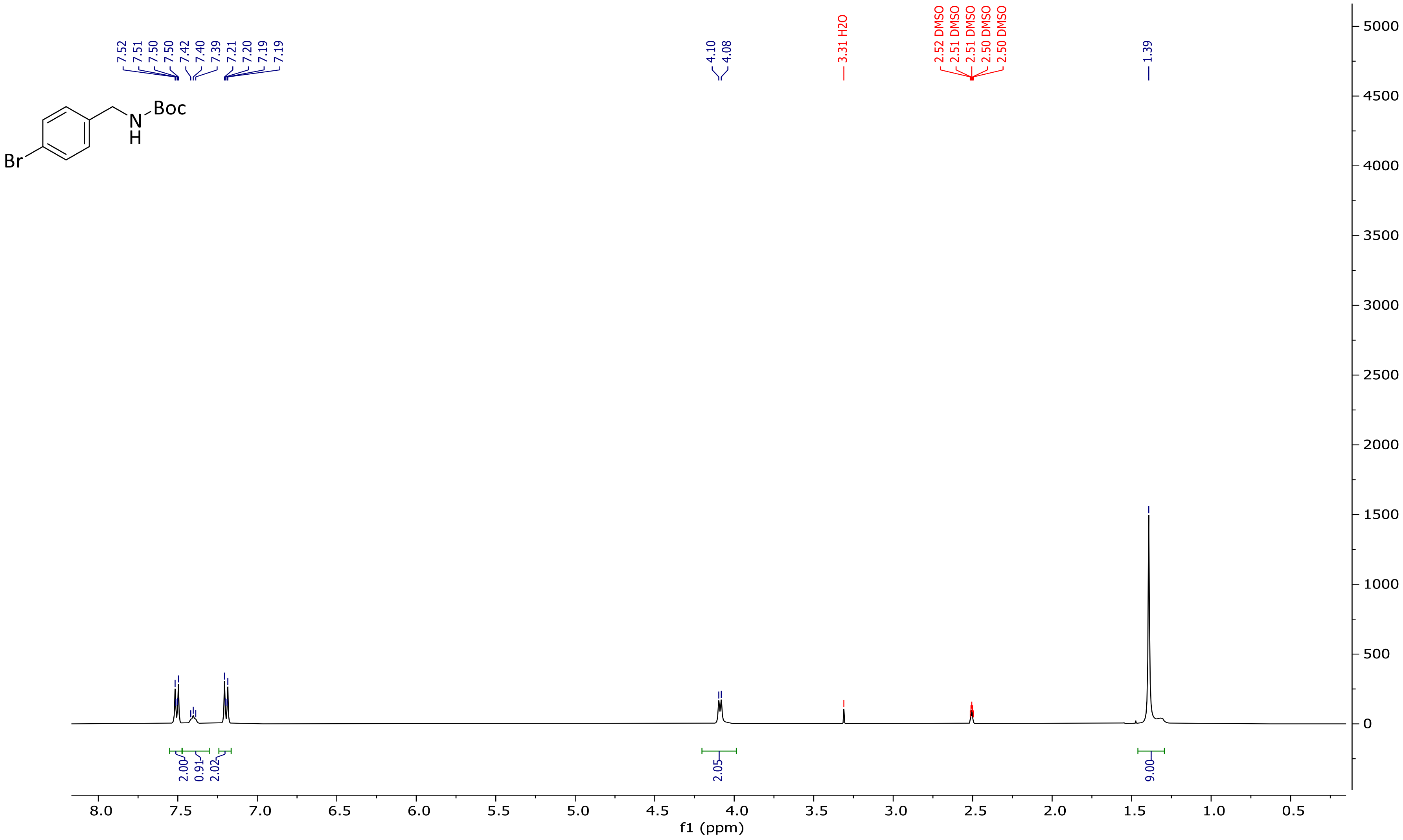

Figure S48. ^{1}H NMR (400 MHz) spectrum of *tert*-butyl (4-bromobenzyl)carbamate (**21**) in DMSO-d_6

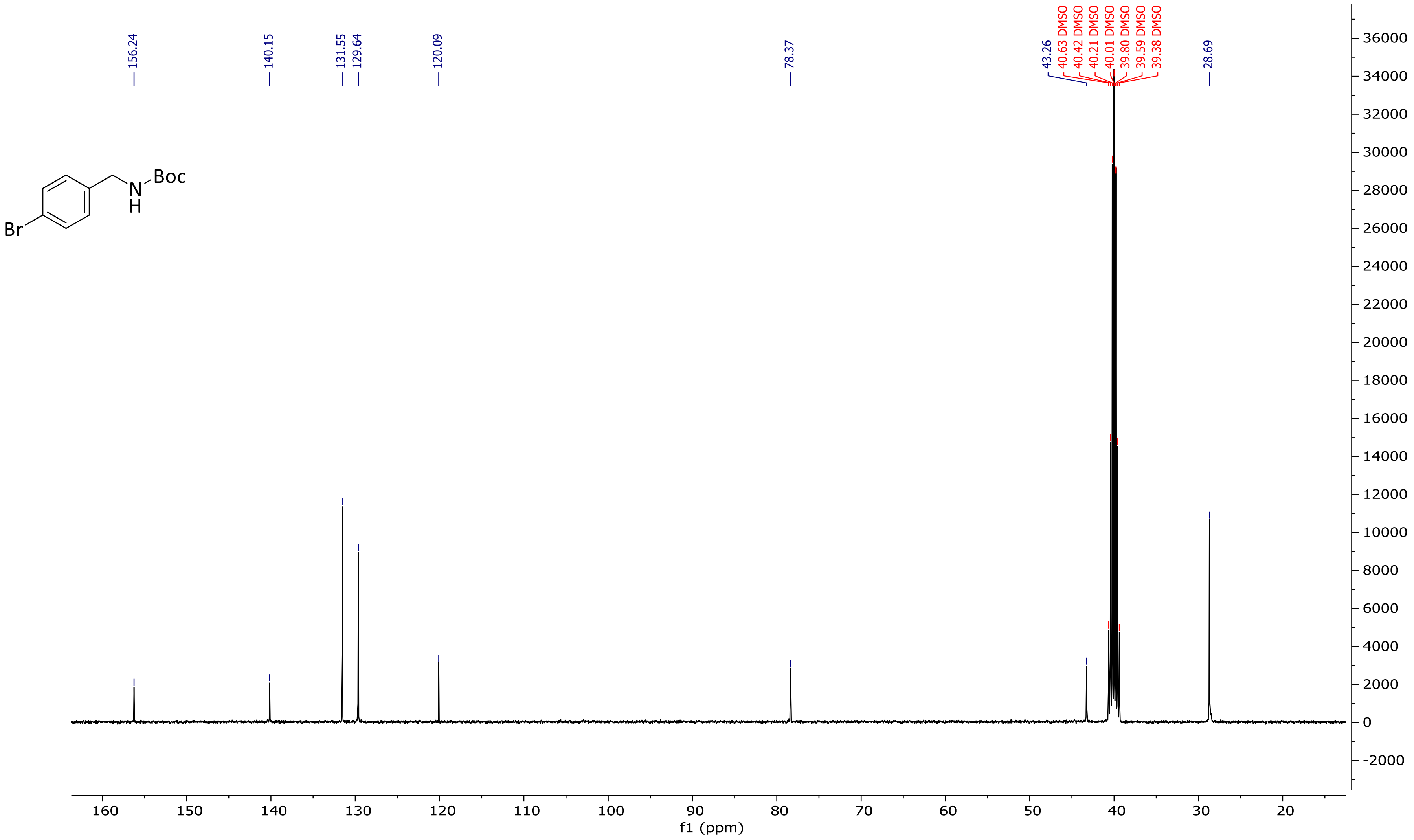

Figure S49. ^{13}C NMR (101 MHz) spectrum of *tert*-butyl (4-bromobenzyl)carbamate (**21**) in DMSO-d_6

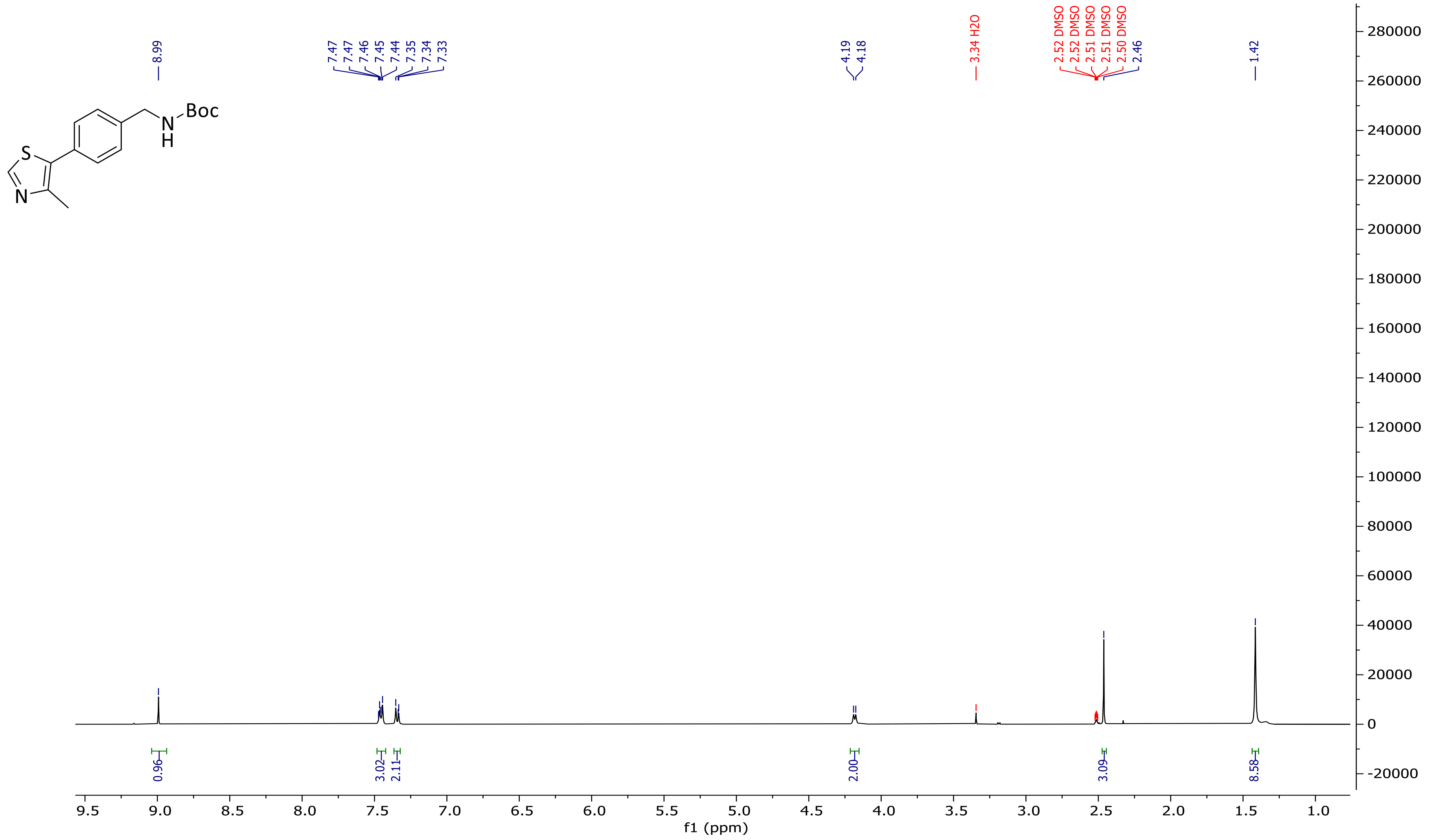

Figure S50. ^{1}H NMR (400 MHz) spectrum of *tert*-butyl (4-(4-methylthiazol-5-yl)benzyl) carbamate (**22**) in DMSO-d_6

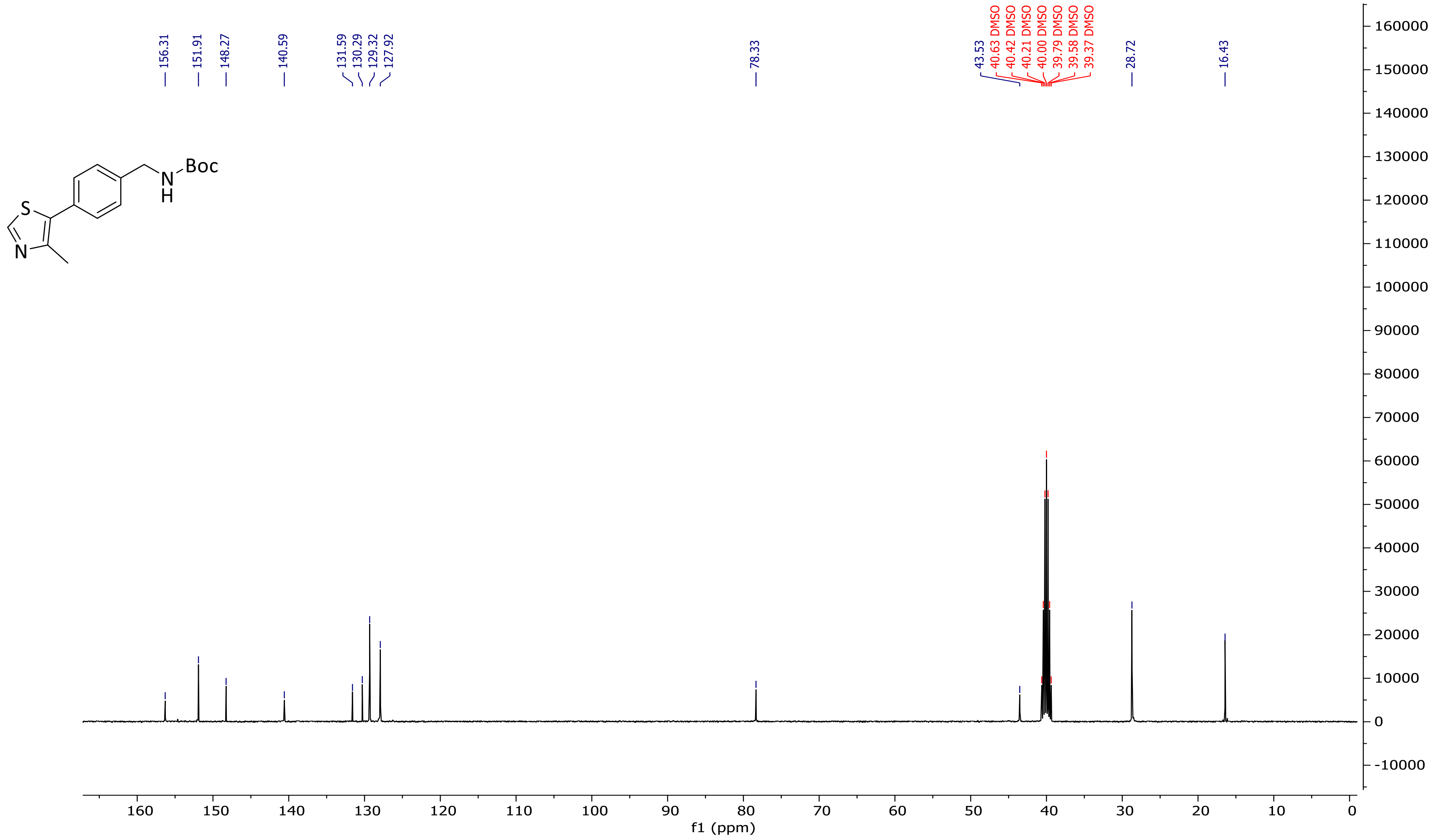

Figure S51. ^{13}C NMR (101 MHz) spectrum of *tert*-butyl (4-(4-methylthiazol-5-yl)benzyl) carbamate (**22**) in DMSO-d_6

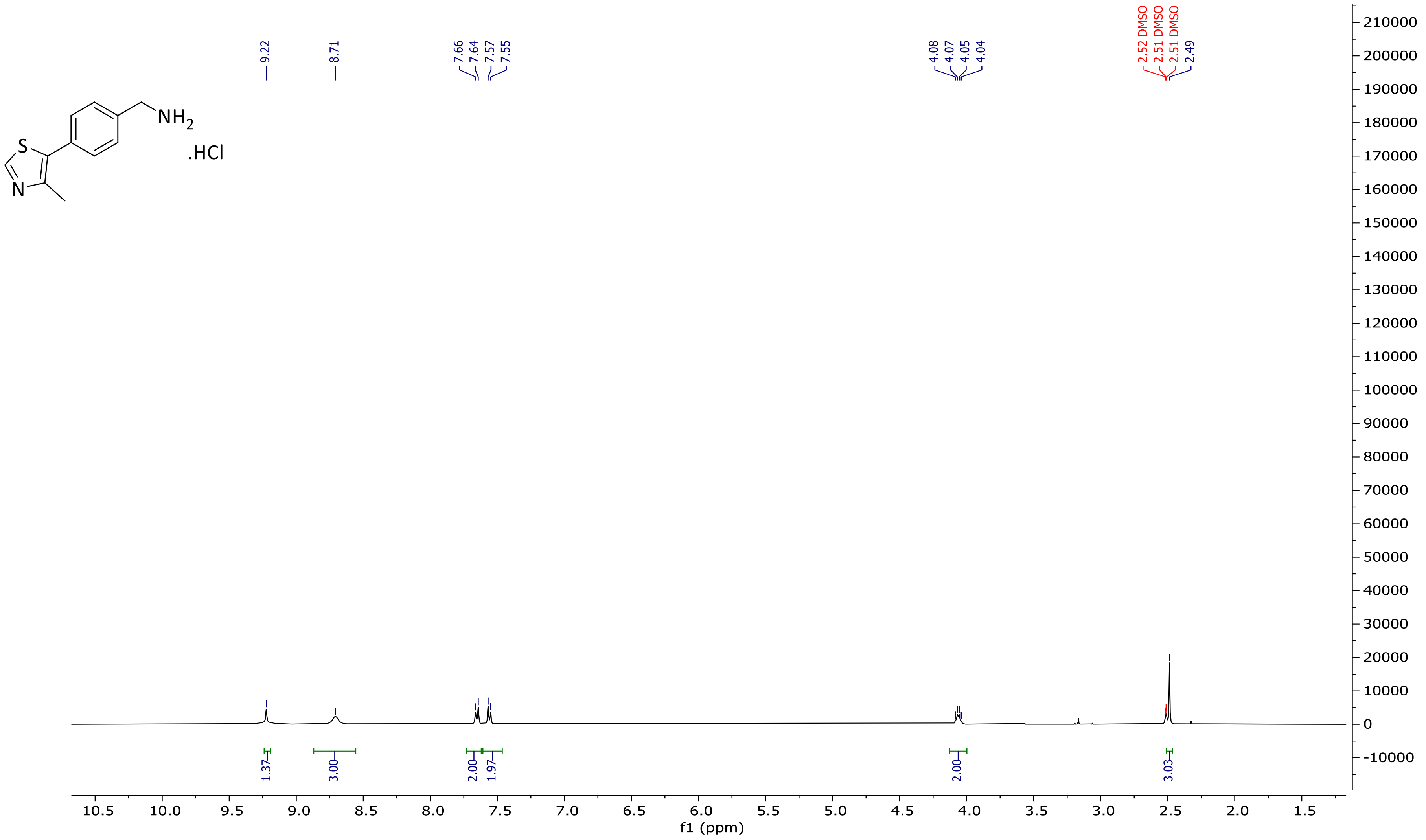

Figure S52. ^{1}H NMR (400 MHz) spectrum of (4-(4-methylthiazol-5-yl)phenyl)methanamine hydrochloride (**23**) in DMSO-d_6

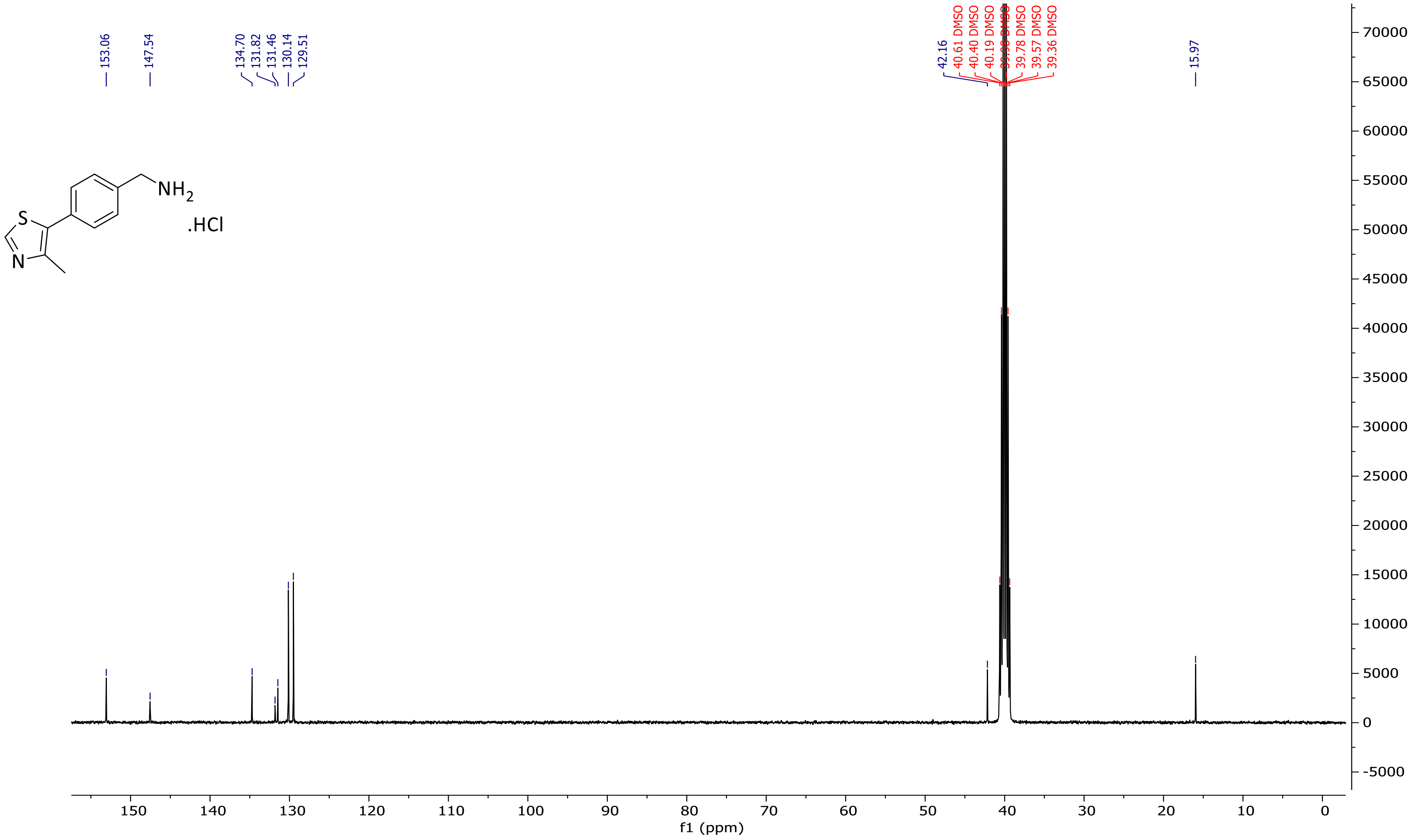

Figure S53. ^{13}C NMR (101 MHz) spectrum of (4-(4-methylthiazol-5-yl)phenyl) methanamine hydrochloride (**23**) in DMSO-d_6

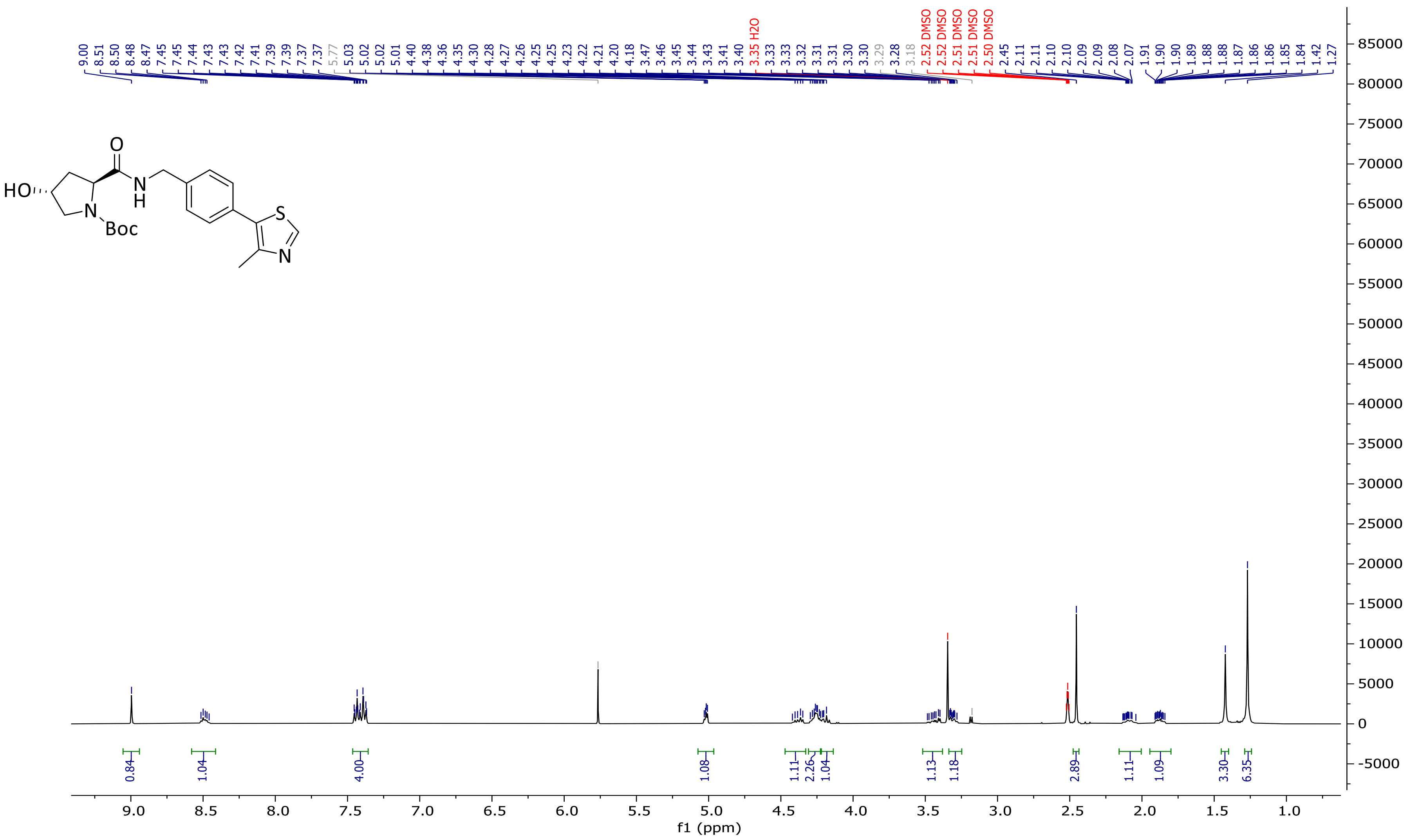

Figure S54. ^{1}H NMR (400 MHz) spectrum of *tert*-butyl (2R)-4-hydroxy-2- ((4-(4-methylthiazol-5-yl)benzyl)carbamoyl) pyrrolidine-1-carboxylate (**24**) in DMSO-d_6

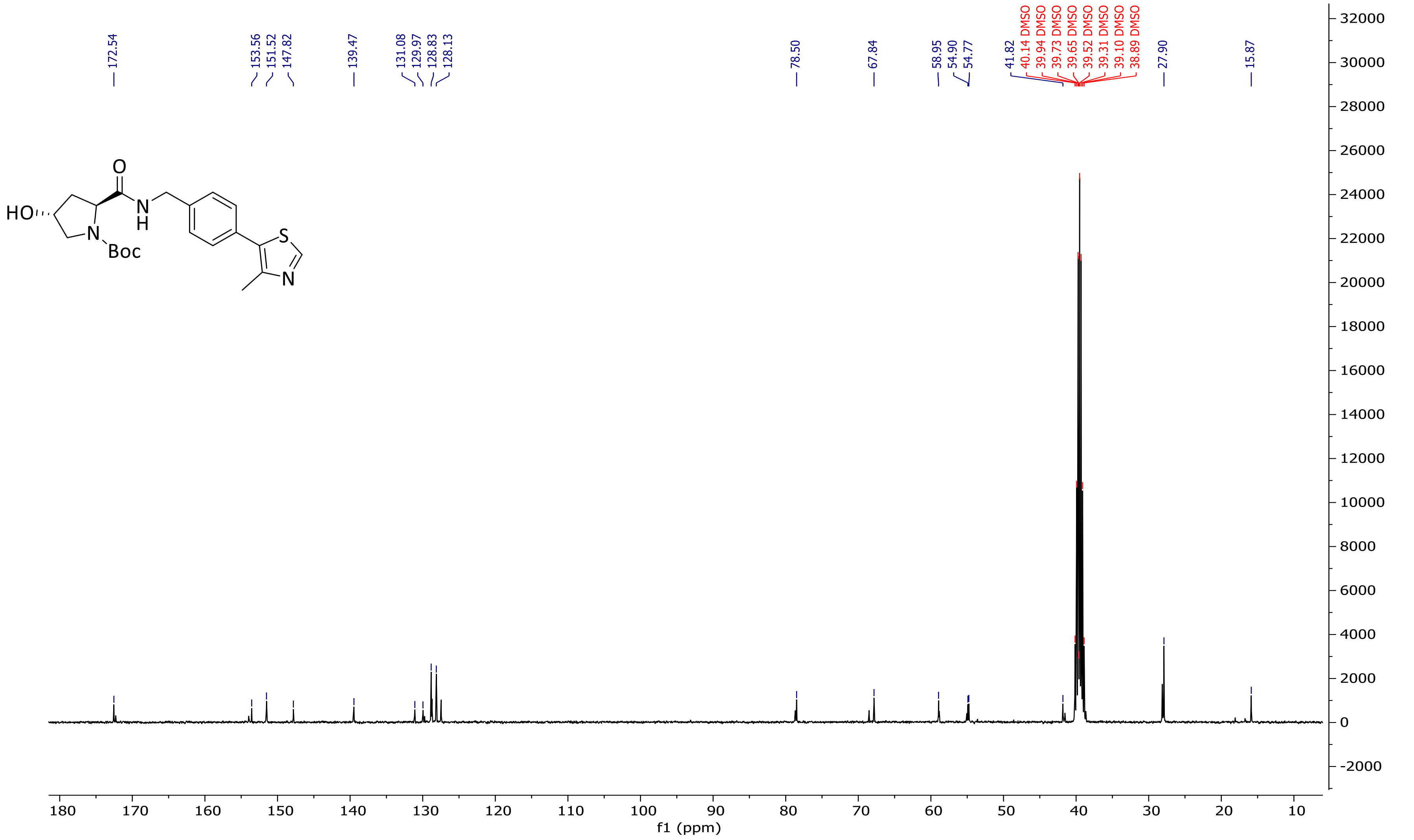

Figure S55. ^{13}C NMR (101 MHz) spectrum of *tert*-butyl (2R)-4-hydroxy-2-((4-(4-methylthiazol-5-yl)benzyl)carbamoyl) pyrrolidine-1-carboxylate (**24**) in DMSO-d_6

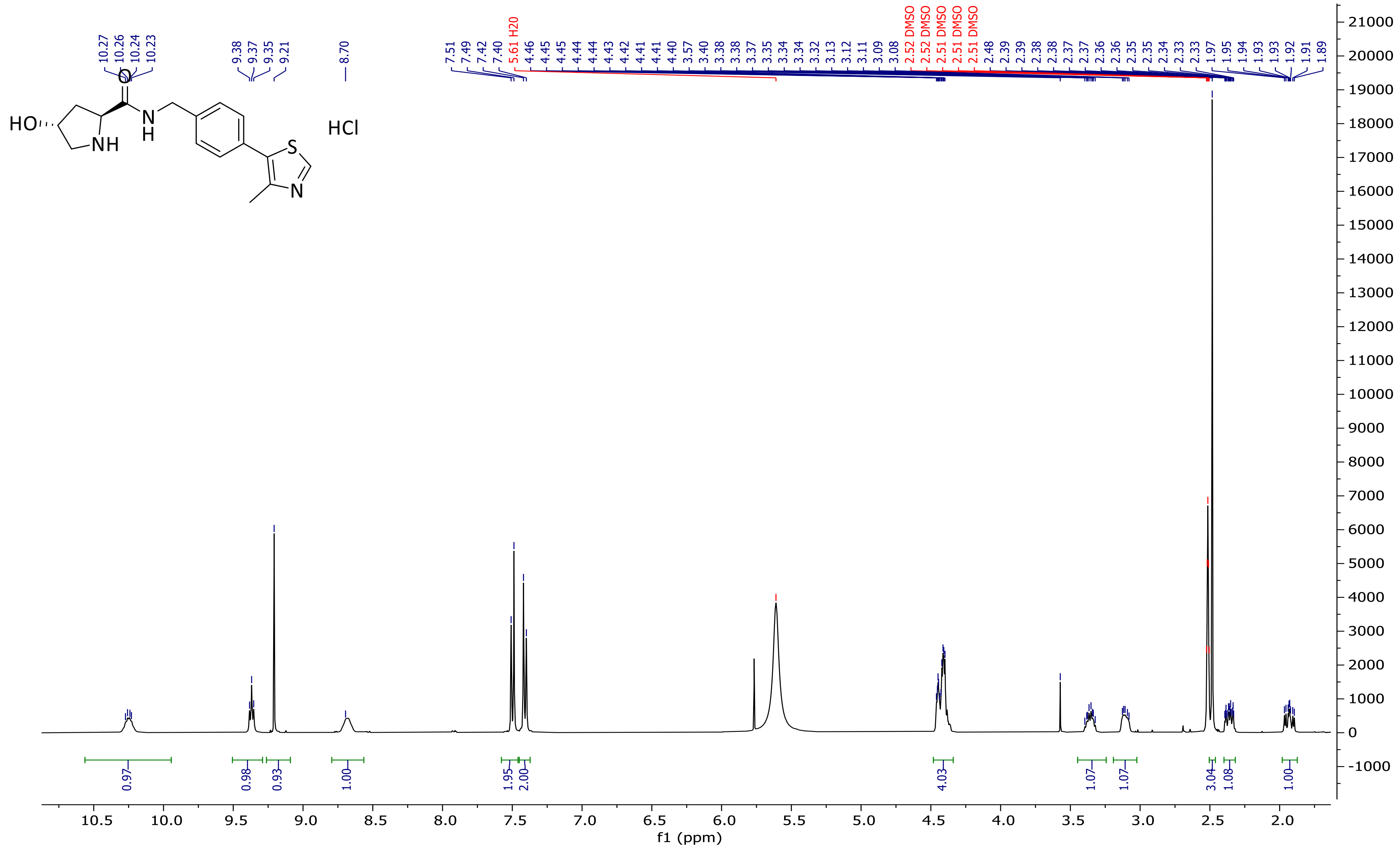

Figure S56. ^{1}H NMR (400 MHz) spectrum of (2S)-4-hydroxy-*N*-(4-(4-methylthiazol-5-yl)benzyl)pyrrolidine-2-carboxamide hydrochloride (**25**) in DMSO-d_6

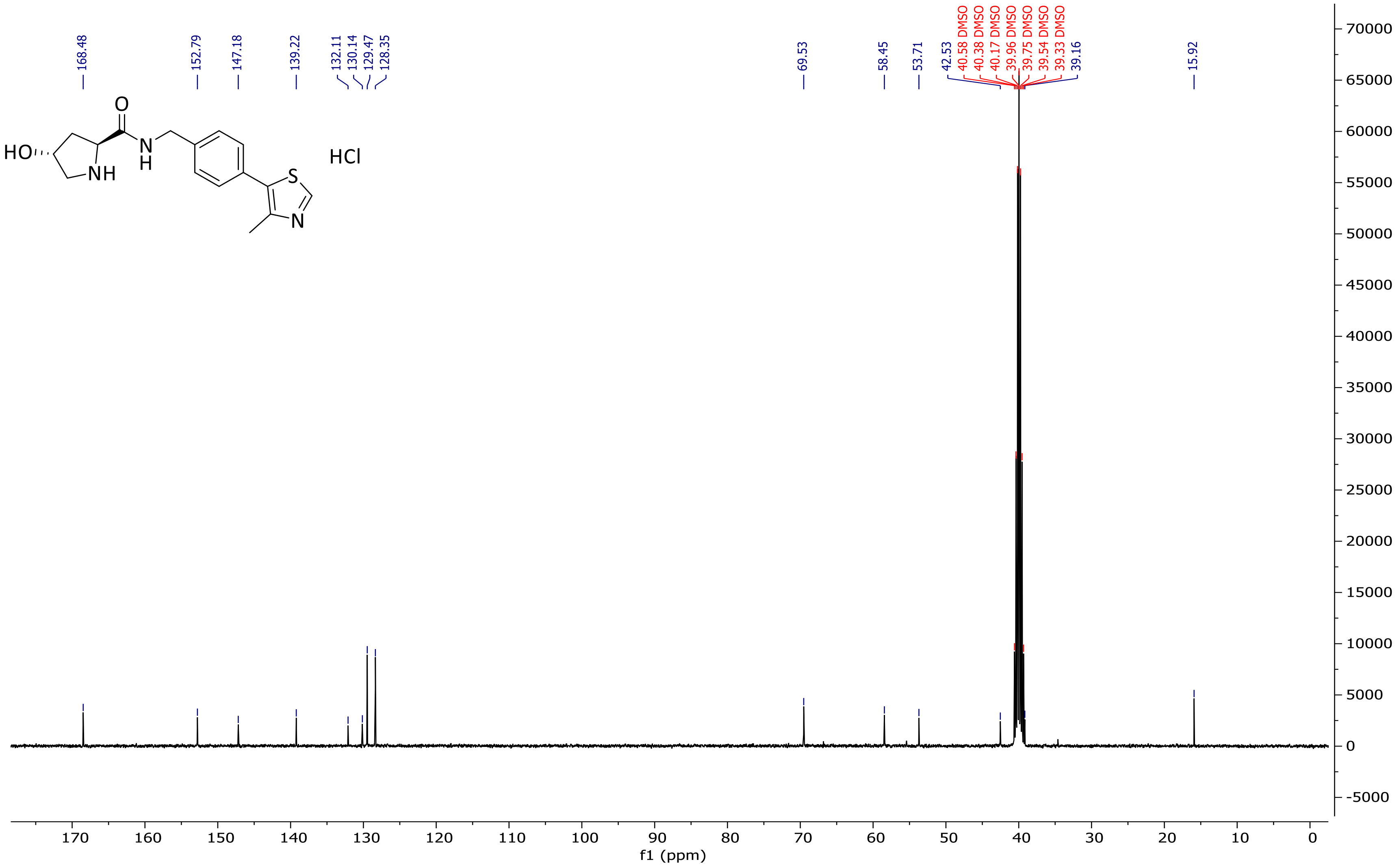

Figure S57. ^{13}C NMR (101 MHz) spectrum of (2S)-4-hydroxy-*N*-(4-(4-methylthiazol-5-yl)benzyl)pyrrolidine-2-carboxamide hydrochloride (**25**) in DMSO-d_6

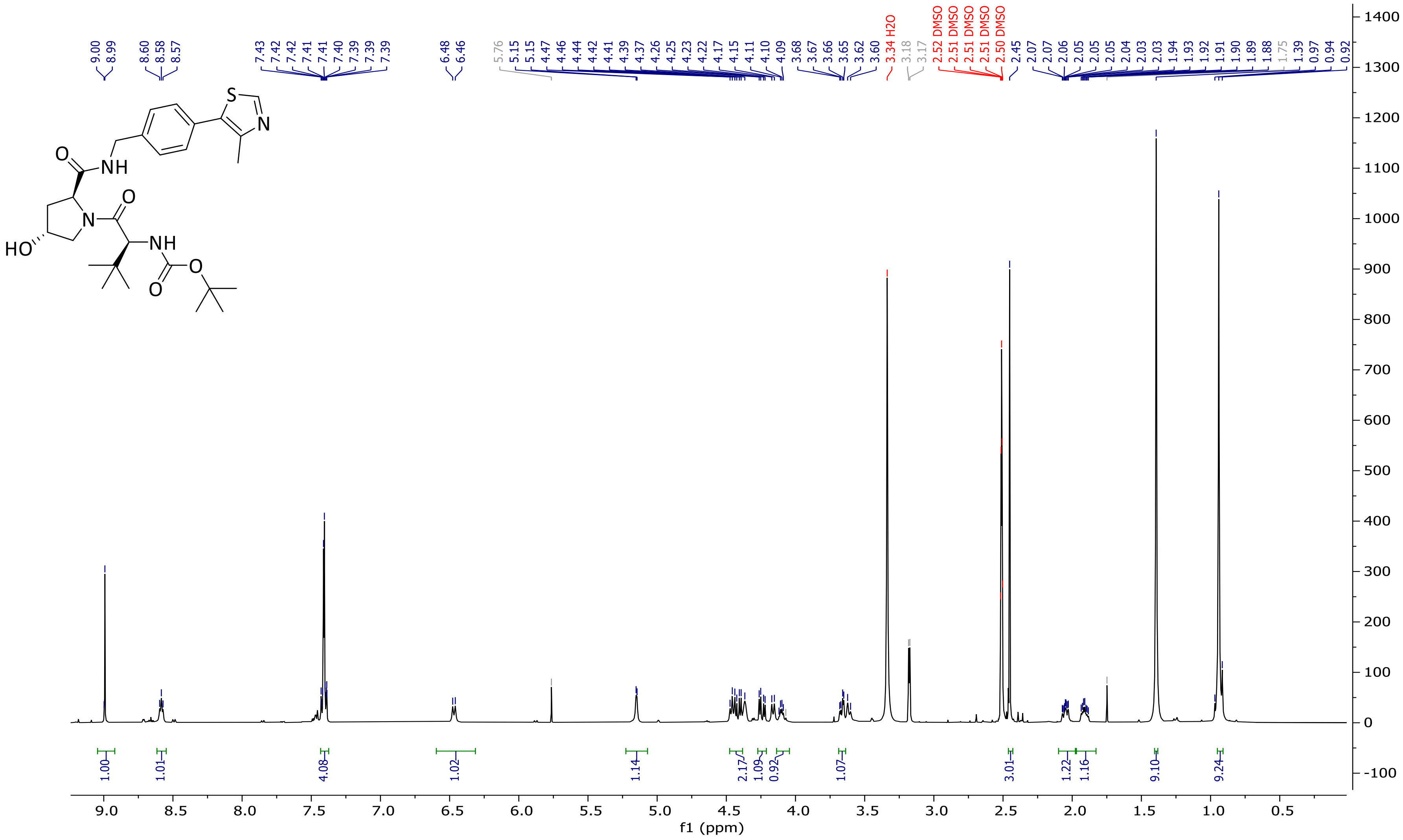

Figure S58. ^{1}H NMR (500 MHz) spectrum of *tert*-butyl (1-((2R)-4-hydroxy-2-((4-(4-methylthiazol-5-yl) benzyl) carbamoyl) pyrrolidin-1-yl)-3,3-dimethyl-1-oxobutan-2-yl)carbamate (**26**) in DMSO-d_6

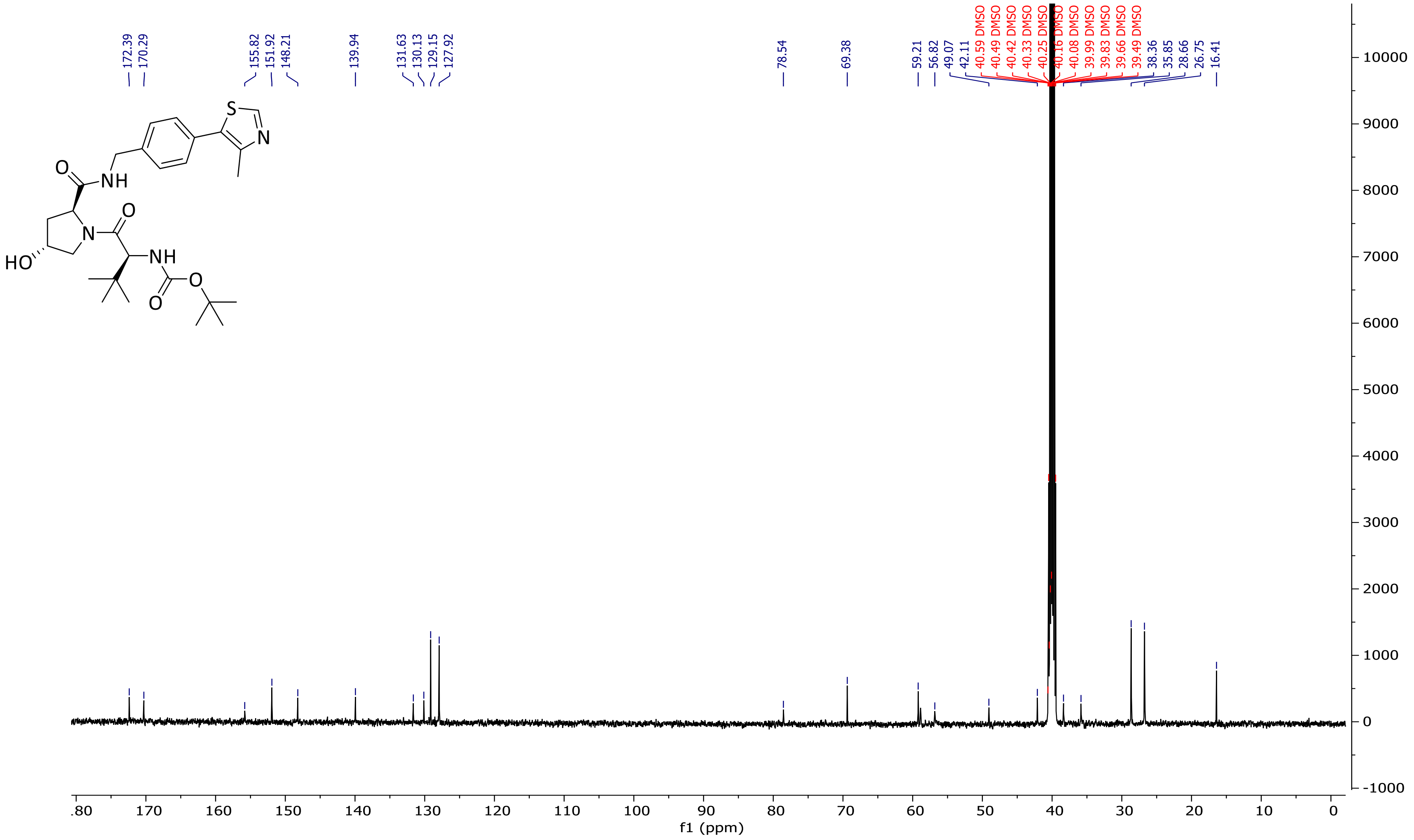

Figure S59. ^{13}C NMR (126 MHz) spectrum of *tert*-butyl (1-((2R)-4-hydroxy-2-((4-(4-methylthiazol-5-yl) benzyl) carbamoyl) pyrrolidin-1-yl)-3,3-dimethyl-1-oxobutan-2-yl)carbamate (**26**) in DMSO-d_6

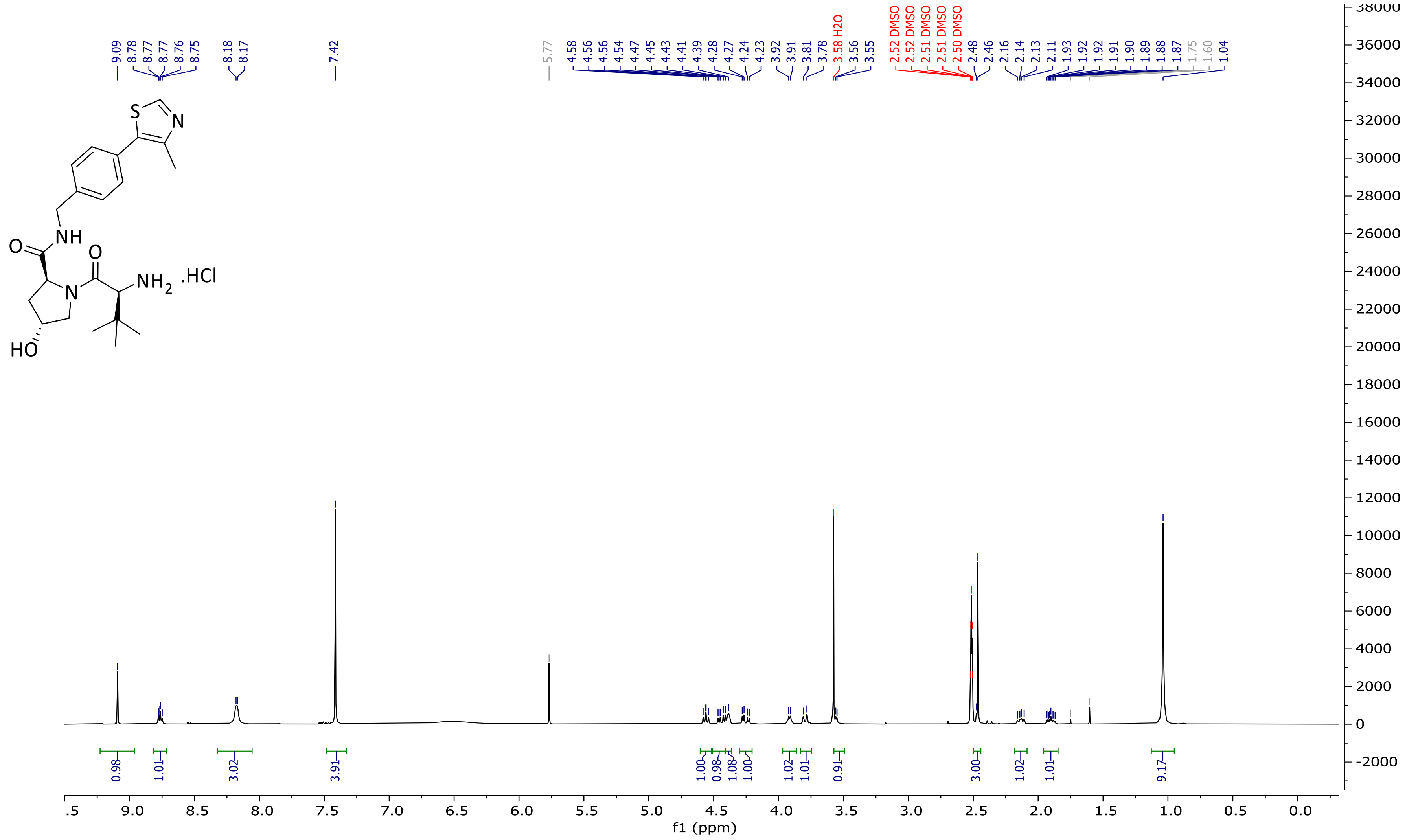

Figure S60. ^{1}H NMR (400 MHz) spectrum of (2R)-1-(2-amino-3,3-dimethylbutanoyl)-4-hydroxy-*N*-(4-(4-methylthiazol-5-yl)benzyl)pyrrolidine-2-carboxamide hydrochloride (**27**) in DMSO-d_6

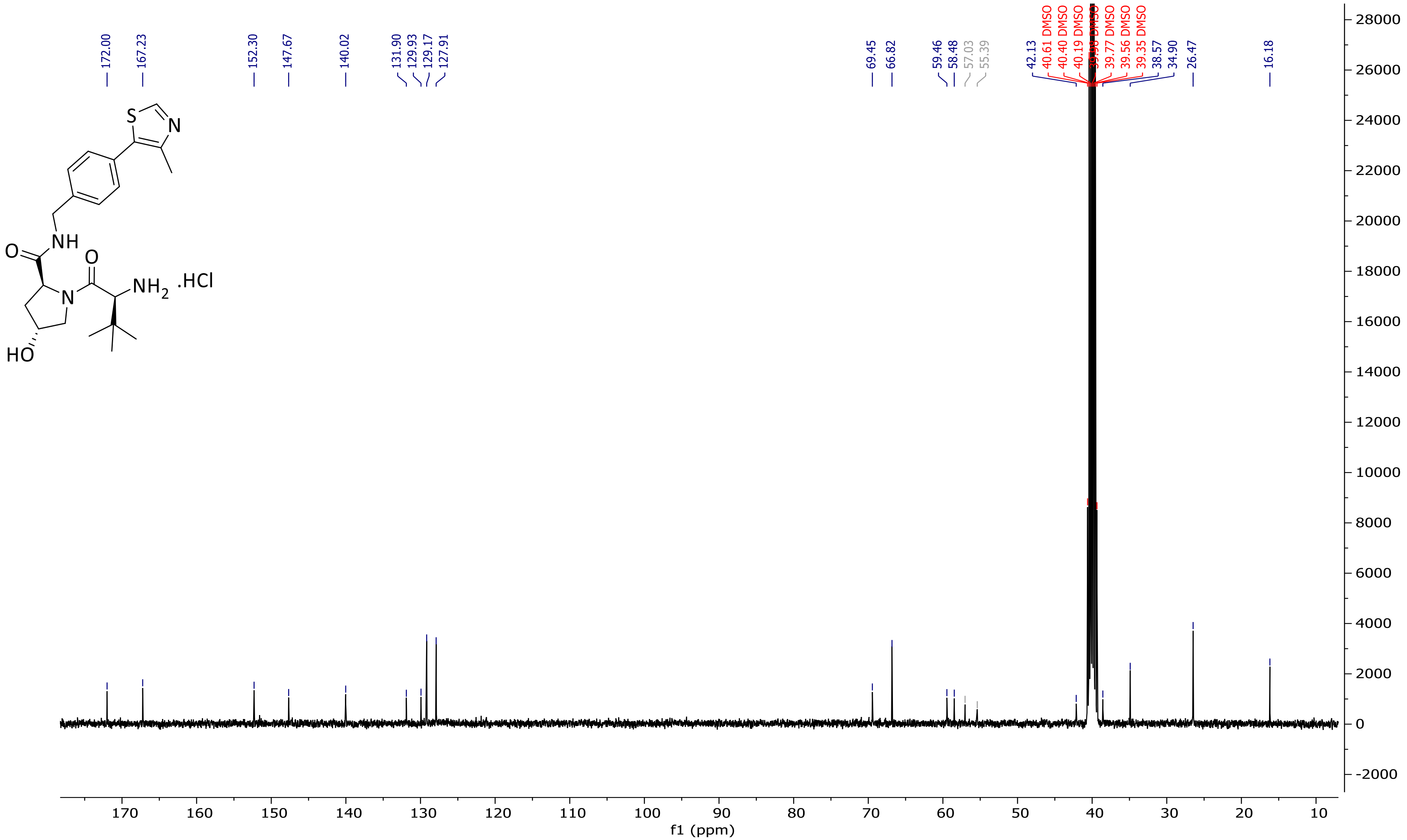

Figure S61. ^{13}C NMR (101 MHz) spectrum of (2R)-1-(2-amino-3,3-dimethylbutanoyl)-4-hydroxy-*N*-(4-(4-methylthiazol-5-yl)benzyl)pyrrolidine-2-carboxamide hydrochloride (**27**) in DMSO-d_6

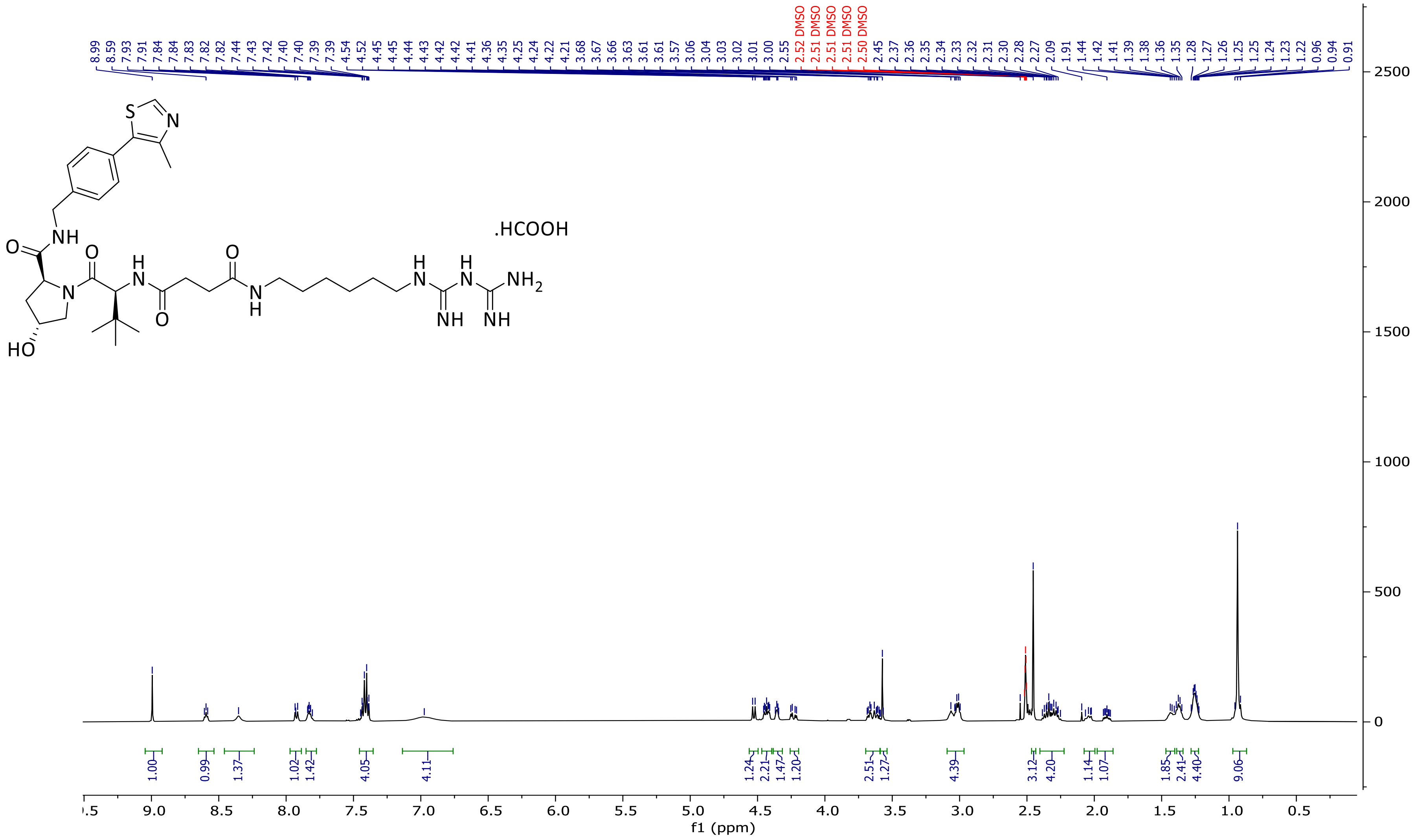

Figure S62. ^{1}H NMR (500 MHz) spectrum of N1-(6-(3-biguanidehexyl)-N4-(1-((2S) -4-hydroxy-2-((4-(4-methylthiazol-5-yl)benzyl)carbamoyl)pyrrolidin-1-yl)-3,3-dimethyl-1-oxobutan-2-yl)succinamide formate (**29**) in DMSO-d_6

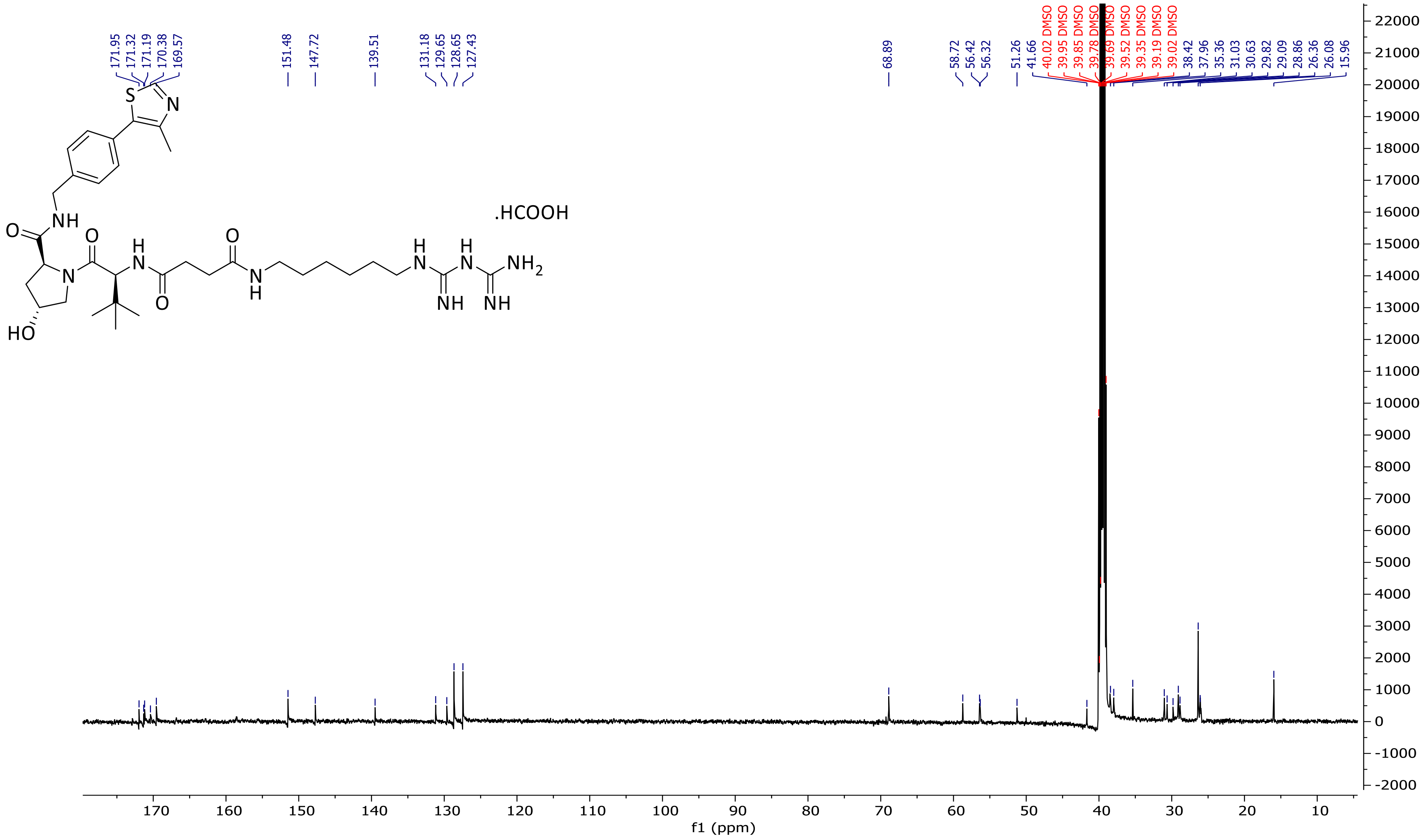

Figure S63. ^{13}C NMR (126 MHz) spectrum of N1-(6-(3-biguanidehexyl)-N4-(1-((2S)- 4-hydroxy-2-((4-(4-methylthiazol-5-yl)benzyl)carbamoyl)pyrrolidin-1-yl)-3,3-dimethyl-1-oxobutan-2-yl)succinamide formate (**29**) in DMSO-d_6

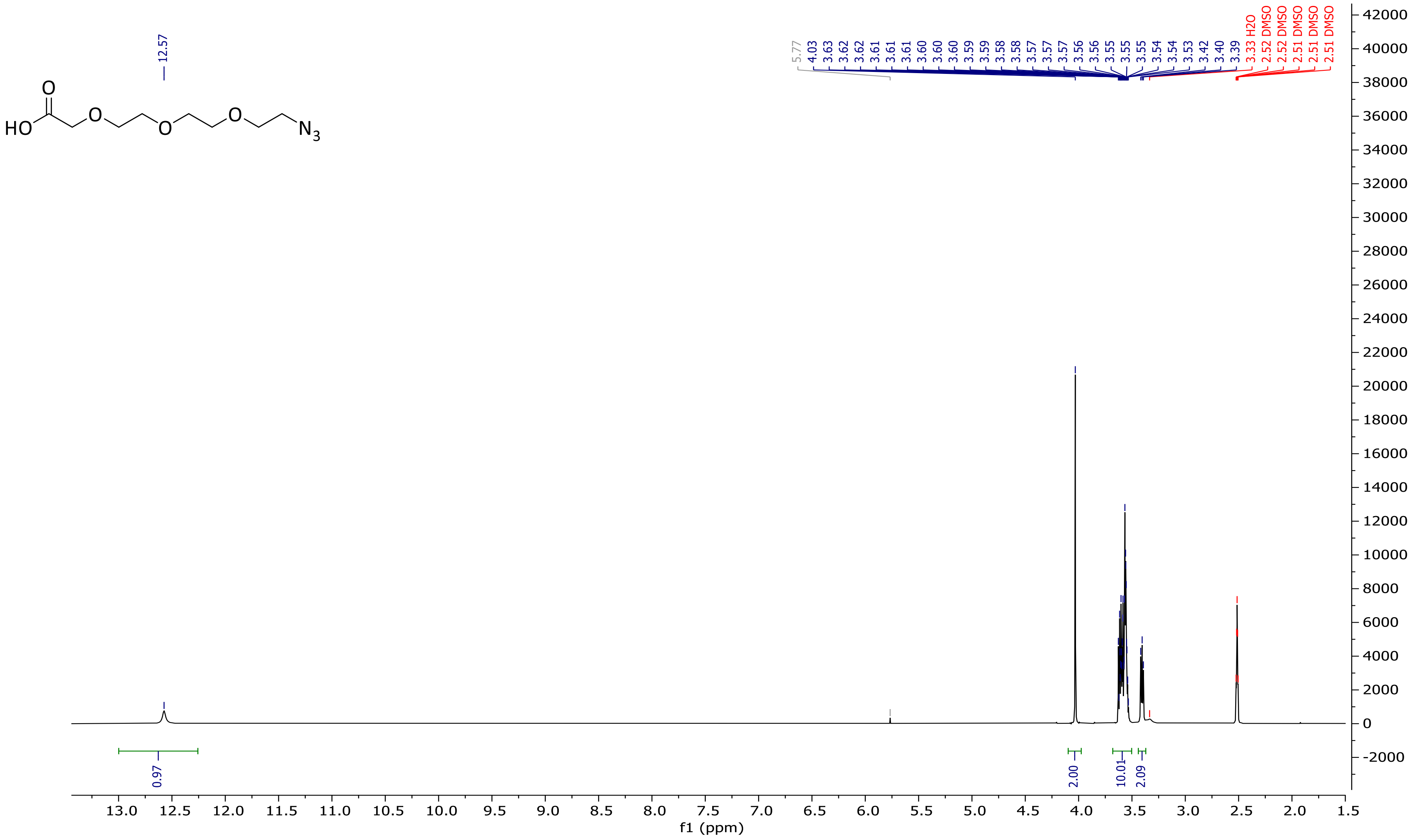

Figure S64. ^{1}H NMR (400 MHz) spectrum of 2-(2-(2-(2-azidoethoxy)ethoxy)ethoxy)acetic acid (**30**) in DMSO-d_6

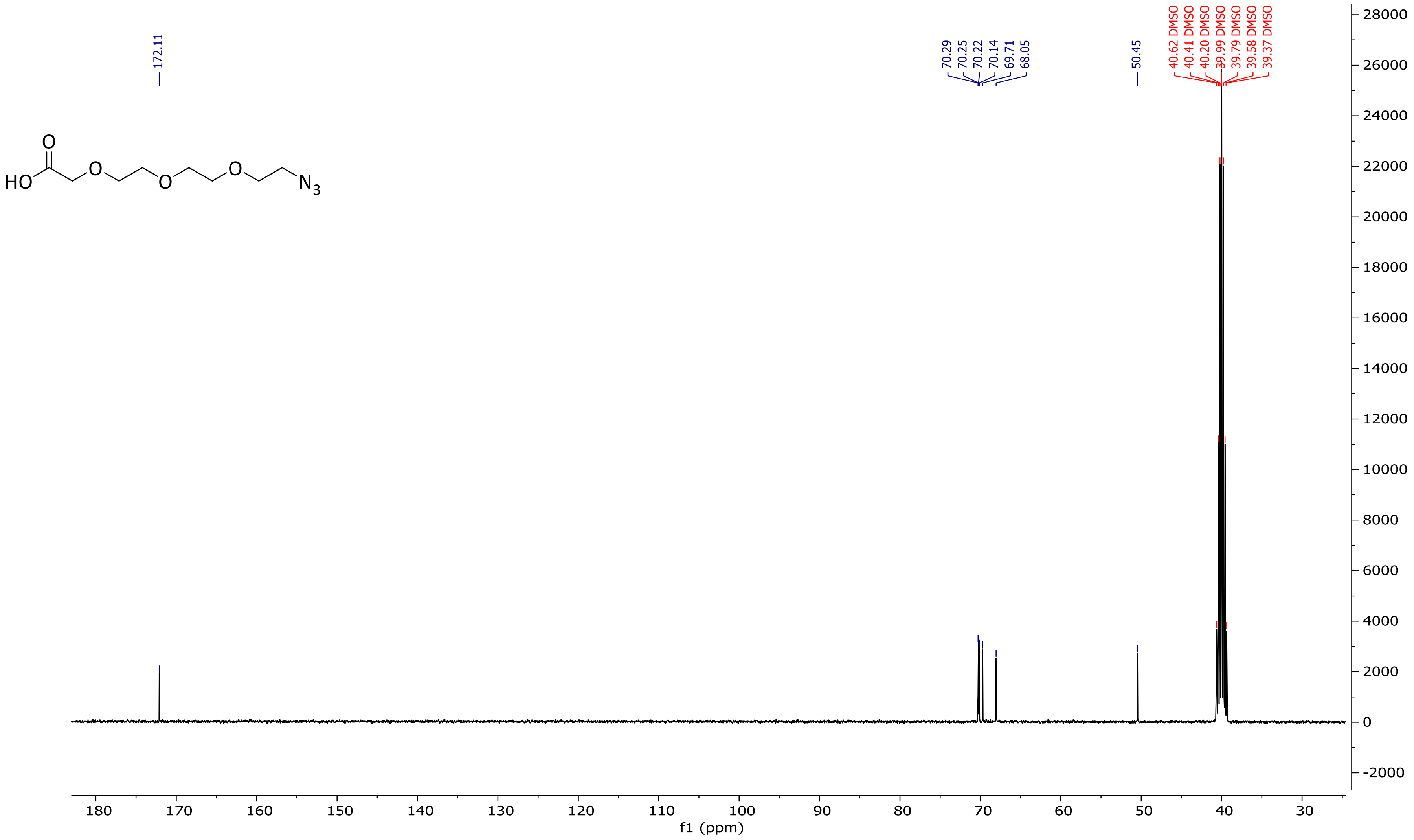

Figure S65. ^{13}C NMR (101 MHz) spectrum of 2-(2-(2-(2-azidoethoxy)ethoxy)ethoxy)acetic acid (**30**) in DMSO-d_6

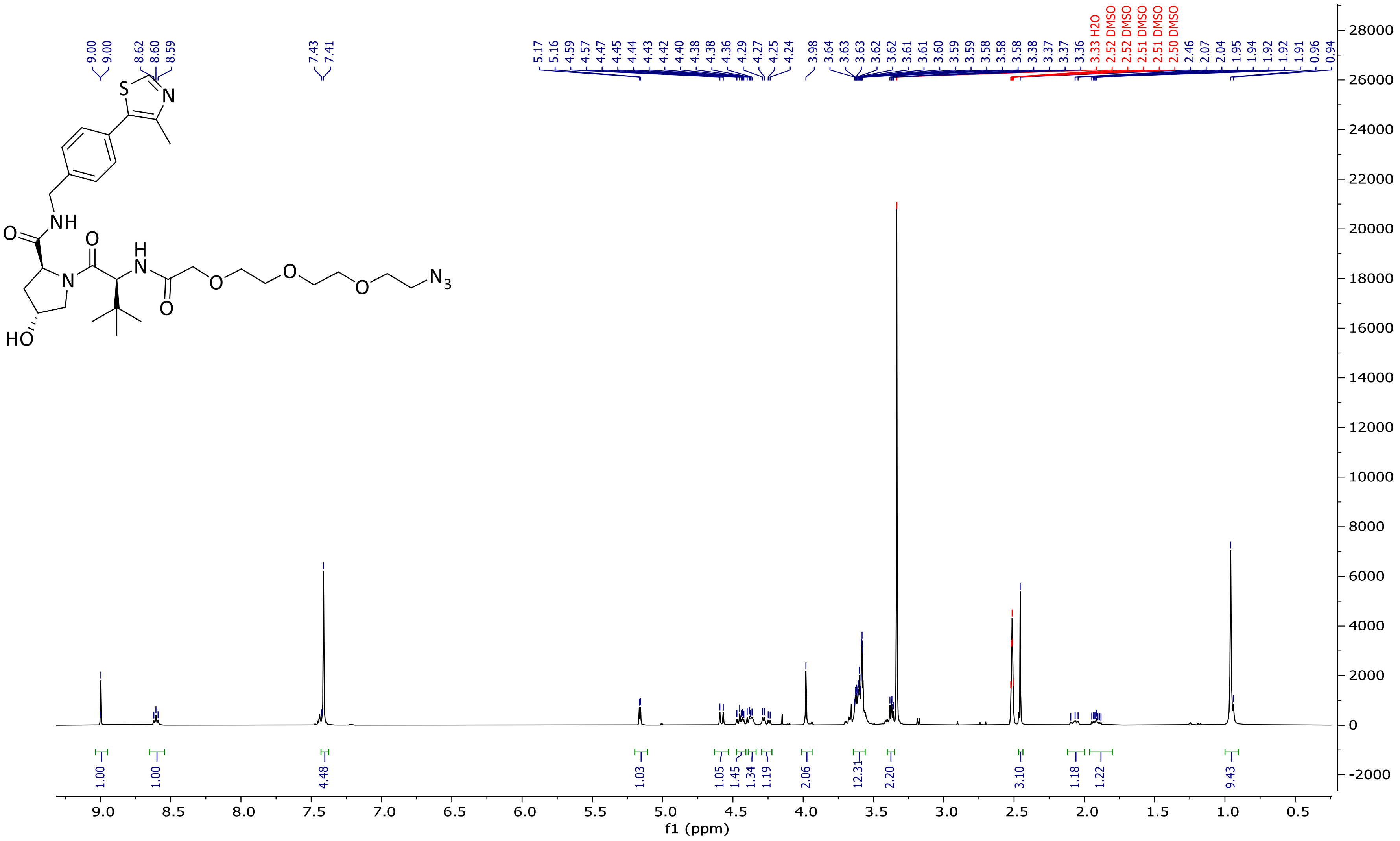

Figure S66. ^{1}H NMR (400 MHz) spectrum of (2S)-1-(14-azido-2-(*tert*-butyl)-4-oxo-6,9,12-trioxa-3-azatetradecanoyl)-4-hydroxy-*N*-(4-(4-methylthiazol-5-yl)benzyl)pyrrolidine-2-carboxamide (**31**) in DMSO-d_6

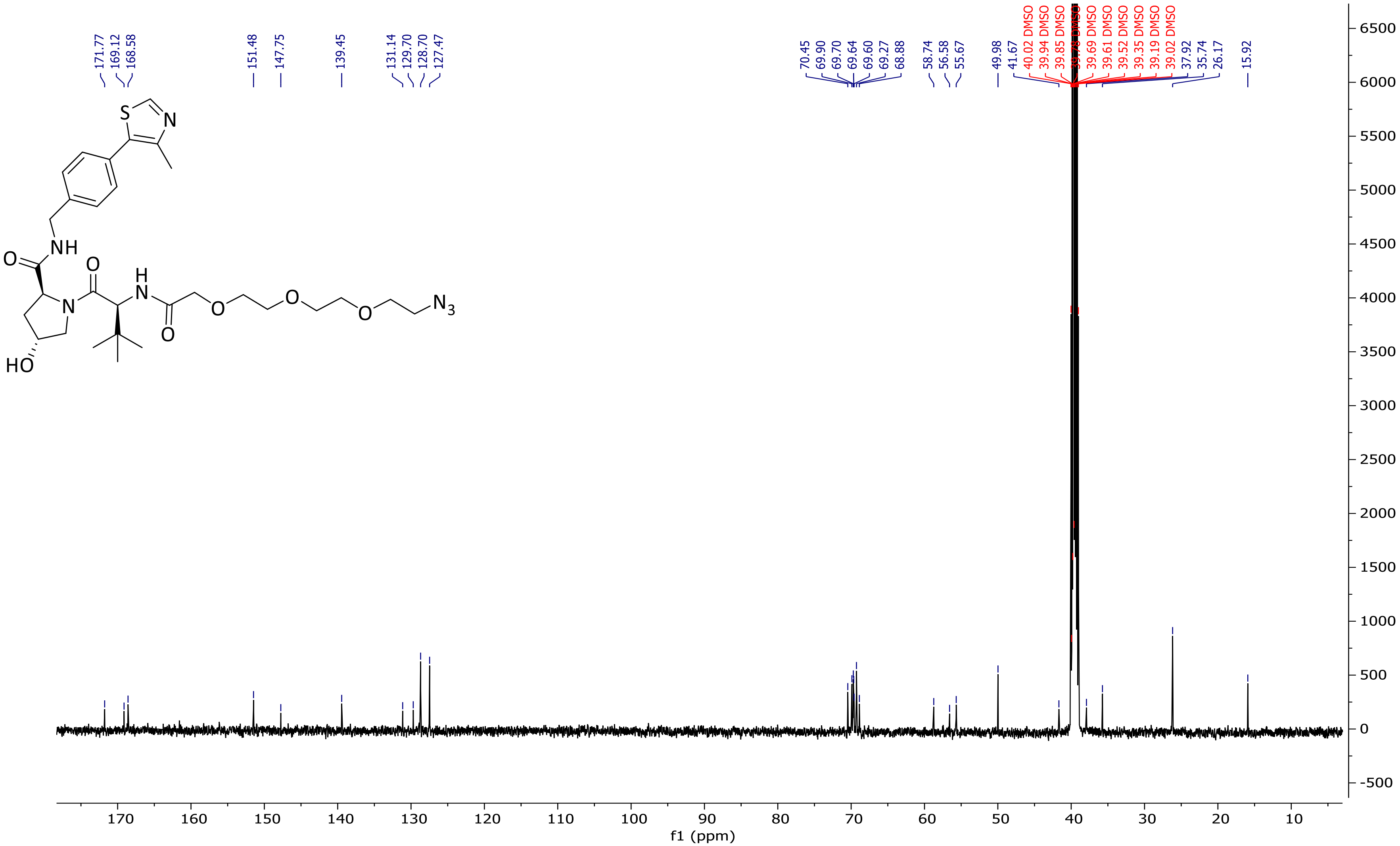

Figure S67. ^{13}C NMR (126 MHz) spectrum of (2S)-1-(14-azido-2-(*tert*-butyl)-4-oxo-6,9,12-trioxa-3-azatetradecanoyl)-4-hydroxy-*N*-(4-(4-methylthiazol-5-yl)benzyl)pyrrolidine-2-carboxamide (**31**) in DMSO-d_6

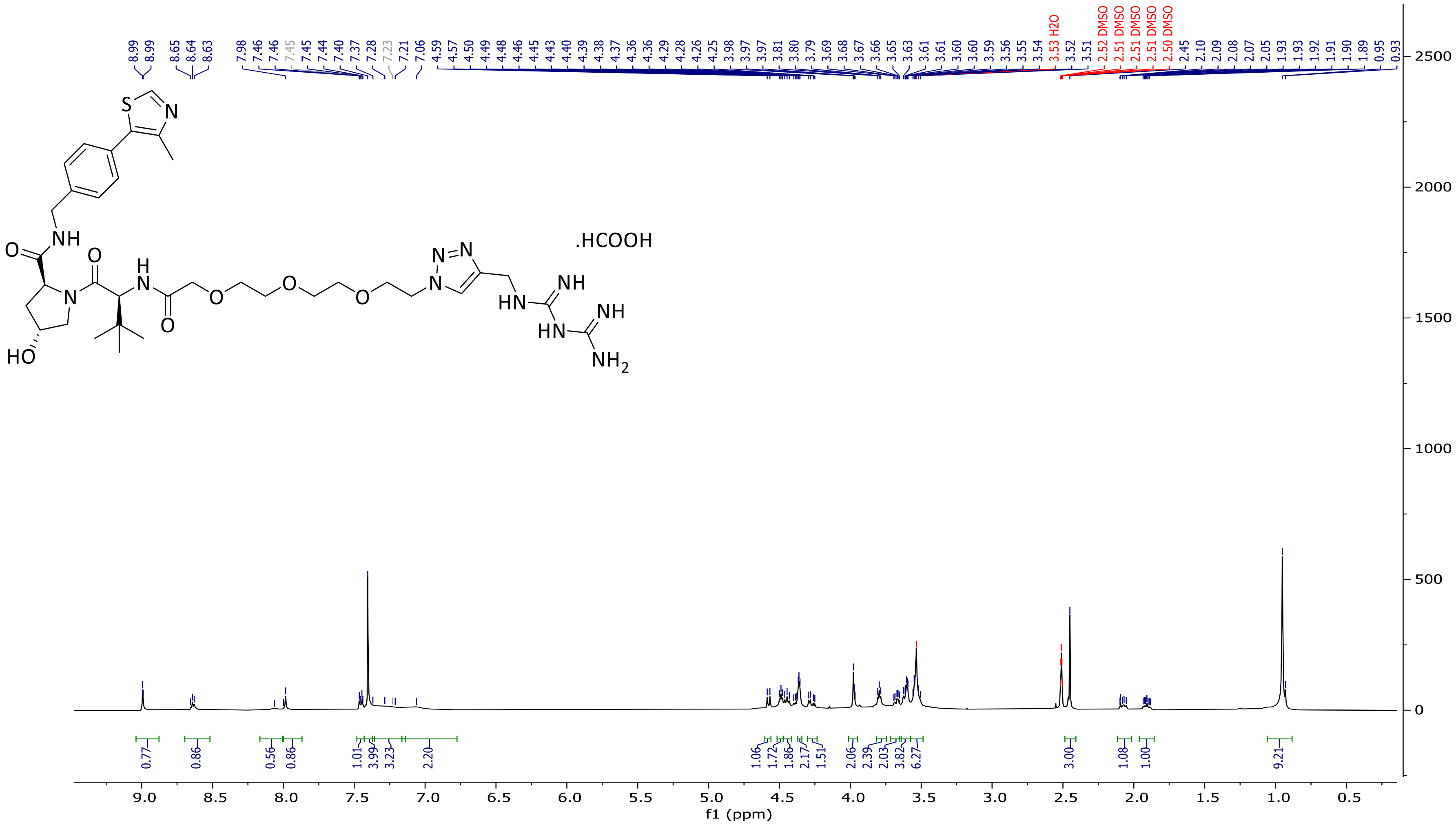

Figure S68. ^{1}H NMR (500 MHz) spectrum of (2S)-1-(2-(*tert*-butyl)-14-(4-((3-biguanide methyl)-1H-1,2,3-triazol-1-yl)-4-oxo-6,9,12-trioxa-3-azatetradecanoyl)-4-hydroxy-*N*-(4-(4-methylthiazol-5-yl)benzyl)pyrrolidine-2-carboxamide formate (**32**) in DMSO-d_6

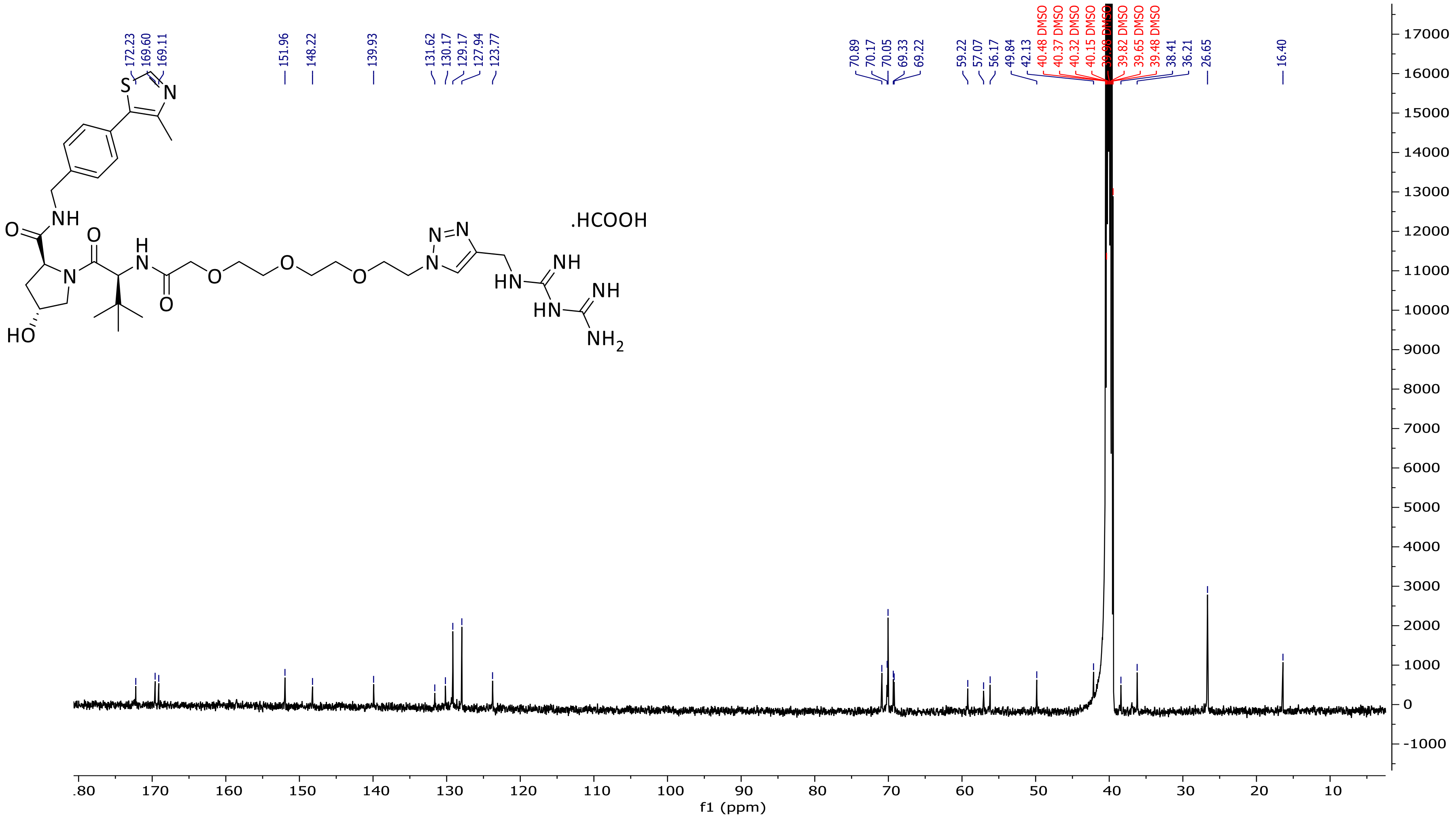

Figure S69. ^{13}C NMR (126 MHz) spectrum of (2S)-1-(2-(*tert*-butyl)-14-(4-((3-biguanide methyl)-1H-1,2,3-triazol-1-yl)-4-oxo-6,9,12-trioxa-3-azatetradecanoyl)-4-hydroxy-*N*-(4-(4-methylthiazol-5-yl)benzyl)pyrrolidine-2-carboxamide formate (**32**) in DMSO-d^6

2. Statistical analyses

Statistical analysis - Effect of Biguanide-Protacs on KP4 cells vs Metformin					
**Dunnett's multiple comparisons test**	**Mean Diff,**	**95,00% CI of diff,**	**Significant?**	**Summary**	**Adjusted P Value**
Metformin vs. 11	0,5097	0,1323 to 0,8872	Yes	**	0,0077
Metformin vs. 12	0,696	0,3185 to 1,073	Yes	***	0,0006
Metformin vs. 16	0,1095	-0,3040 to 0,5230	No	ns	0,9421
Metformin vs. 20	0,2189	-0,1946 to 0,6323	No	ns	0,4749
Metformin vs. 28	0,6405	0,2270 to 1,054	Yes	**	0,0028
Metformin vs. 32	0,1741	-0,2394 to 0,5876	No	ns	0,6858

Statistical analysis - Effect of Biguanide-Protacs and their controls on P-AMPK/AMPK levels					
**Dunnett's multiple comparisons test**	**Mean Diff,**	**95,00% CI of diff,**	**Significant?**	**Summary**	**Adjusted P Value**
DMSO vs. Metformine	-0,497	-1,221 to 0,2271	No	ns	0,2486
DMSO vs. 8	-0,2305	-0,9546 to 0,4935	No	ns	0,897
DMSO vs. 27	0,09976	-0,6243 to 0,8238	No	ns	0,9994
DMSO vs. 11	-0,9423	-1,666 to -0,2182	Yes	*	0,0101
DMSO vs. 12	-0,7377	-1,462 to -0,01363	Yes	*	0,0453
DMSO vs. 16	-0,008771	-0,7328 to 0,7153	No	ns	>0,9999
DMSO vs. 20	0,2863	-0,4378 to 1,010	No	ns	0,7633
DMSO vs. 29	0,2648	-0,4592 to 0,9889	No	ns	0,8196
DMSO vs. 32	-0,002598	-0,7267 to 0,7215	No	ns	>0,9999
DMSO vs. 34	0,3365	-0,3876 to 1,061	No	ns	0,6211

Statistical analysis - Effect of Compound 12 on OXPHOS protein levels						
**Protein CV**	**Dunnett's multiple comparisons test**	**Mean Diff,**	**95,00% CI of diff,**	**Significant?**	**Summary**	**Adjusted P Value**
	0 µM vs. 5 µM	-0,09435	-0,5344 to 0,3457	No	ns	0,912
	0 µM vs. 50 µM	0,2077	-0,2324 to 0,6478	No	ns	0,4382
	0 µM vs. 100 µM	0,1656	-0,2745 to 0,6057	No	ns	0,614
	0 µM vs. 250 µM	-0,1333	-0,5734 to 0,3068	No	ns	0,7607
	0 µM vs. 500 µM	0,2826	-0,1575 to 0,7227	No	ns	0,2209
**Protein CIII**	**Dunnett's multiple comparisons test**	**Mean Diff,**	**95,00% CI of diff,**	**Significant?**	**Summary**	**Adjusted P Value**
	0 µM vs. 5 µM	-0,03307	-0,7557 to 0,6896	No	ns	0,9998
	0 µM vs. 50 µM	0,05801	-0,6647 to 0,7807	No	ns	0,9984
	0 µM vs. 100 µM	0,1283	-0,5944 to 0,8509	No	ns	0,9545
	0 µM vs. 250 µM	0,2372	-0,4855 to 0,9599	No	ns	0,7104
	0 µM vs. 500 µM	0,353	-0,3696 to 1,076	No	ns	0,4114
**Protein CII**	**Dunnett's multiple comparisons test**	**Mean Diff,**	**95,00% CI of diff,**	**Significant?**	**Summary**	**Adjusted P Value**
	0 µM vs. 5 µM	0,2373	-0,2822 to 0,7569	No	ns	0,4638
	0 µM vs. 50 µM	0,3605	-0,1590 to 0,8801	No	ns	0,1777
	0 µM vs. 100 µM	0,3192	-0,2004 to 0,8387	No	ns	0,2481
	0 µM vs. 250 µM	0,3556	-0,1639 to 0,8752	No	ns	0,185
	0 µM vs. 500 µM	0,4292	-0,09035 to 0,9487	No	ns	0,1019
**Protein CIV**	**Dunnett's multiple comparisons test**	**Mean Diff,**	**95,00% CI of diff,**	**Significant?**	**Summary**	**Adjusted P Value**
	0 µM vs. 5 µM	-0,173	-0,9017 to 0,5556	No	ns	0,8782
	0 µM vs. 50 µM	-0,2742	-1,003 to 0,4545	No	ns	0,614
	0 µM vs. 100 µM	0,2913	-0,4374 to 1,020	No	ns	0,5681
	0 µM vs. 250 µM	0,5247	-0,2040 to 1,253	No	ns	0,1592
	0 µM vs. 500 µM	0,7161	-0,01257 to 1,445	Yes	*	0,0536
**Protein CI**	**Dunnett's multiple comparisons test**	**Mean Diff,**	**95,00% CI of diff,**	**Significant?**	**Summary**	**Adjusted P Value**
	0 µM vs. 5 µM	0,3097	-0,3836 to 1,003	No	ns	0,4813
	0 µM vs. 50 µM	0,1157	-0,5776 to 0,8089	No	ns	0,9639
	0 µM vs. 100 µM	0,6122	-0,08102 to 1,305	No	ns	0,0804
	0 µM vs. 250 µM	0,7532	0,05996 to 1,446	Yes	*	0,0356
	0 µM vs. 500 µM	0,8741	0,1808 to 1,567	Yes	*	0,0185

	Effect of compounds on pAMPK/AMPK levels - Band intensity						
	N=1			N=2			
Band Label	P AMPK / GAPDH	AMPK / GADPH	PAMPK / AMPK	P AMPK / GAPDH	AMPK / GADPH	PAMPK / AMPK	Average
DMSO	1	1	1	1	1	1	1
Metformine	1.731945166	1.141410005	1.517373388	1.550065849	1.049749406	1.476605598	1.49698949
$CRBN_L$-OH	1.124742308	1.249150538	0.900405735	1.399626806	0.896819637	1.560655842	1.23053078
VHL_L-NH_2	0.904309081	1.103940187	0.819164926	1.012434593	1.031713591	0.981313615	0.90023927
**11**	1.303587837	0.677321931	1.924620742	1.333305828	0.680265883	1.959977503	1.94229912
**12**	1.321691701	0.700051297	1.887992647	2.466249248	1.55365013	1.587390366	1.73769150
**16**	0.695500895	0.621730703	1.118652966	1.221099248	1.358453	0.898889581	1.00877127
**20**	0.794983365	0.98918833	0.803672406	0.745687923	1.195375995	0.623810355	0.71374138
**29**	0.97498053	1.14413219	0.85215724	0.968255338	1.566322019	0.618171312	0.73516427
**32**	0.832376976	1.19766232	0.695001389	1.786401154	1.363461619	1.310195409	1.00259839
**34**	1.040116696	1.370792003	0.758770619	0.733959399	1.291602813	0.568254724	0.66351267

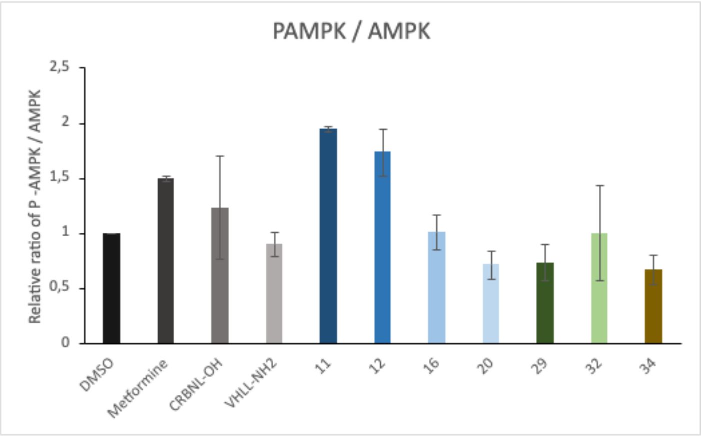

Supplement: Supplementary file 1 [file molecules-29-05329-s001.zip › molecules-3265032-supplementary.pdf]
